# Chemical Constituents from Soft Coral *Clavularia* spp. Demonstrate Antiproliferative Effects on Oral Cancer Cells

**DOI:** 10.3390/md21100529

**Published:** 2023-10-08

**Authors:** Ming-Ya Cheng, Ya-Ting Chuang, Hsueh-Wei Chang, Zheng-Yu Lin, Ching-Yeu Chen, Yuan-Bin Cheng

**Affiliations:** 1 Department of Marine Biotechnology and Resources, National Sun Yat-Sen University, Kaohsiung 80424, Taiwan; mina78976@gmail.com (M.-Y.C.); m105020014@nsysu.edu.tw (Z.-Y.L.); 2 Department of Biomedical Science and Environmental Biology, PhD Program in Life Sciences, College of Life Science, Kaohsiung Medical University, Kaohsiung 80708, Taiwan; ashleybox26@gmail.com (Y.-T.C.); changhw@kmu.edu.tw (H.-W.C.); 3 Center for Cancer Research, Kaohsiung Medical University, Kaohsiung 80708, Taiwan; 4 Department of Medical Research, Kaohsiung Medical University Hospital, Kaohsiung 80708, Taiwan; 5 Department of Physical Therapy, Tzu-Hui Institute of Technology, Pingtung 92641, Taiwan; chingyeu1971@yahoo.com.tw; 6 Graduate Institute of Natural Products, College of Pharmacy, Kaohsiung Medical University, Kaohsiung 80708, Taiwan

**Keywords:** eudensamane-type sesquiterpene lactone, dolabellane, *Clavularia* spp., cytotoxicity

## Abstract

Five new eudensamane-type sesquiterpene lactones, clasamanes A–E (**1**–**5**), three new dolabellane-type diterpenes, clabellanes A–C (**6**–**8**), and fifteen known compounds (**9**–**23**) were isolated from the ethanolic extract of Taiwanese soft coral *Clavularia* spp. The structures of all undescribed components (**1**–**8**) were determined by analysis of IR, mass, NMR, and UV spectroscopic data. The absolute configuration of new compounds was determined by using circular dichroism and DP4+ calculations. The cytotoxic activities of all isolated marine natural products were evaluated. Compound **7** showed a significant cytotoxic effect against oral cancer cell line (Ca9-22) with an IC_50_ value of 7.26 ± 0.17 μg/mL.

## 1. Introduction

In south–central Asia, people are susceptible to oral cancers because of the usage of areca nuts as chewing gum. Taiwan has one of the world’s highest incidences of oral cancer, which ranks fourth in the cause of cancer death among male Taiwanese [1]. In Taiwan, about 3000 deaths yearly are due to oral cancers. The treatments for oral cancer are usually combined surgery and chemotherapy; however, chemotherapy drugs sometimes produce adverse effects [2]. It is necessary to discover new anti-oral cancer drugs.

Marine sessile animals like sponges, soft corals, tunicates, and zoanthids are known to produce diverse secondary metabolites. Octacoral is one of the most abundant sources of bioactive marine natural products (MNPs) with unique backbones. Since 1977, the soft corals of the genus *Clavularia* have been found to have different kinds of MNPs, such as diterpenoids [3,4,5], sesquiterpenoids [6], prostanoids [7,8], and steroids [9]. Those MNPs usually demonstrate considerable cytotoxic effects against several cancer cell lines. For example, dolabellane-type diterpenes could significantly inhibit P-388 leukemia cells with an ED_50_ value of 0.052 μg/mL [4]. Eudensamane-type sesquiterpene lactones were found to inhibit the growth of cancer cell lines. In our previous study on Taiwanese marine invertebrates, the methanol extract of *Clavularia inflata* exerts an apoptotic effect and DNA damage to oral cancer cells [10]. These findings propel us to conduct the natural product investigation of this coral extract.

## 2. Results

**Figure 1:**
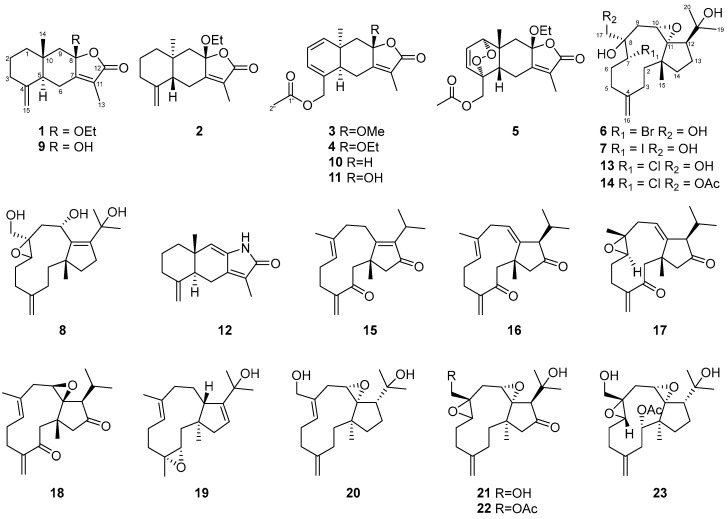
Structures of MNPs **1**–**23** isolated from *Clavularia* spp.

In this contribution, we describe the isolation, structural determination and cytotoxic evaluation from the bioactive coral extract of *Clavularia* spp. In total, eight new (**1**–**8**) and fifteen known compounds (**9**–**23**) were isolated from repeated column chromatography (Figure 1). Those known compounds were identified as atractylenolides III (**9**) [11], tubipolide A (**10**) [12], tubipolide C (**11**) [12], atractylenolactam (**12**) [13], clavinflol B (**13**) [5], clavinflol B monoacetate (**14**) [5], (1*R*,12*R*)-dolabella-4(16),7,10-triene-3,13-dione (**15**) [4], (1R*)-dolabella-4(16),7,11(12)-triene-3,13-dione (**16**) [4], (1R*,7R*,8S*,-12R*)-dolabella-4(16),10-diene-7,8-epoxy-3,13-dione (**17**) [4], (1R*,10R*,11S*,12R*)-dolabella-4(16),7-diene-10,11-epoxy-3,13-dione (**18**) [4], 2-((*E*)-(1*S*,3*R*,5*R*,12*S*)-1,5,9-trimethyl-4-oxa-tricyclo [10.3.0.0^3,5^]pentadeca-8,13-dien-13-yl)-propan-2-ol (**19**) [14], 2-((*E*)-(1*R*,3*R*,12*S*,15*S*)-5-hydroxymethyl-12-methyl-9-methylene-2-oxa-tricyclo [10.3.0.0^1,3^]pentadec-5-en-15-yl)-propan-2-ol (**20**) [15], stolonidiol (**21**) [16], stolonidiol monoacetate (**22**) [16], and clavinflol A (**23**) [5].

**Figure 2:**
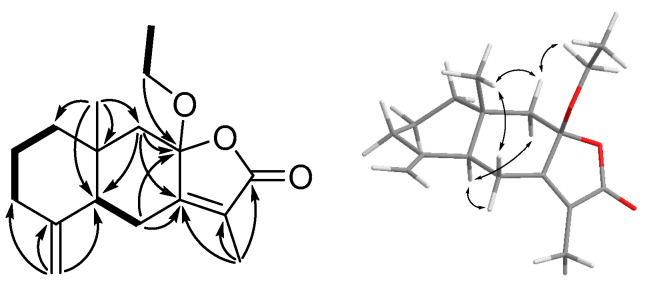
Key COSY (bold), HMBC (arrow), and NOESY (double arrow) correlations of **1**.

Clasamane A (**1**) was isolated as a colorless oil, and the molecular formula, C_17_H_24_O_3_ (Δ = 6), was assigned from its HRESIMS data (*m*/*z* 299.1616 [M + Na]^+^, which was calculated for 299.1618). The UV maximum absorptions at λ_max_ 218 and 282 nm implied that compound **1** belongs to eudensamane-type sesquiterpene lactone [11], whereas the IR absorptions indicated the presence of lactone functionality (1751 cm^−1^). The ^1^H NMR spectrum (Table 1) displayed the signals for two methyls (δ 1.86 (s) (H_3_-13) and δ 0.99 (s) (H_3_-14)), an ethoxy group (δ 3.46 (m), δ 3.28 (m) (H_2_-1′) and δ 1.18 (t, *J* = 7.0) (H_3_-2′)), and an exomethylene (δ 4.86 (s) and δ 4.58 (s) (H_2_-15)). The ^13^C NMR and DEPT spectra (Table 2) displayed 17 carbons, which were assigned to one ester carbonyl (δ 171.9 (C-12)), three sp^2^ non-protonated carbons (δ 159.9 (C-7), δ 148.7 (C-4), and δ 123.8 (C-11)), one exomethylene (δ 106.7 (C-15)), two quaternary carbons (δ 106.2 (C-8), and δ 36.8 (C-10)), six methylenes (δ 58.7 (C-1′), δ 50.2 (C-9), δ 41.4 (C-1), δ 36.0 (C-3), δ 25.0 (C-6), and 22.3 (C-2)), one methine (δ 51.8 (C-5)), and three methyls (δ 16.4 (C-14), δ 15.2 (C-2′), and δ 8.3 (C-13)). The aforementioned NMR data are similar to those of atractylenolides III (**9**), except for an additional ethoxy group that was found in the ^1^H and ^13^C spectra. The planar structure of **1** was established by the COSY and HMBC experimental data (Figure 2). In the COSY spectrum, two proto sequences of H_2_-1 (δ 1.56 and δ 1.23)/H_2_-2 (δ 1.63 and δ 1.48)/H_2_-3 (δ 2.36 and δ 1.96) and H-5 (δ 1.82)/H_2_-6 (δ 2.62 and δ 2.27) were observed. These two proton sequences and the HMBC correlations from H_3_-14 to C-1, C-5, C-9, and C-10; from H_2_-15 to C-3, C-4, and C-5; from H_2_-6 to C-7 and C-8; from H_3_-13 to C-7, C-11, and C-12; and from H_2_-9 (δ 2.36 and δ 1.41) to C-8 and C-7 can be used to construct the carbon skeleton of eudensamane-type sesquiterpene lactone. The attachment of an ethoxy group at C-8 was confirmed by virtue of the HMBC correlations from H_2_-1′ to C-8. The relative configuration of **1** was determined by the NOESY correlations (Figure 2). NOE cross-peaks of H_3_-14/H-9*β* (δ 2.36)/H-1′ (δ 3.46) suggested these protons are on the *β*-orientation. The NOESY correlations of H-6*α* (δ 2.27)/H-5/H-9*α* (δ 1.41) suggested they are α-oriented. The absence of NOESY correlation between H_3_-14 and H-5 indicated the opposite side of these protons and a *trans*-decalin moiety in **1**. The absolute configuration of **1** was determined to be (5*S*,8*S*,10*R*) by comparing the experimental ECD and NMR data of **1** with that of **9** (Figure S1). On the basis of the above spectroscopic data analysis, the structure of **1** was determined as shown.

Clasamane B (**2**) was an isomer of **1** because it possessed the same molecular formula as **1** and similar NMR spectrometric data. The most noticeable variations between **2** and **1** were the carbon chemical shifts of C-5 (δ 41.6 for **2** and δ 51.8 for **1**) and C-14 (δ 21.0 for **2** and δ 16.4 for **1**), which implied the configuration of these two positions might change. The absence of NOESY correlation (Figure S2) between H_3_-14 (δ 0.64) and H-5 (δ 2.74) indicated the *trans* conformation of the decalin moiety, like that of **1**. The configuration of C-8 was defined to be *S* due to the similar NMR data of α-methyl-α,*β*-unsaturated-*γ*-hydroxy-*γ*-lactone moiety between **2** and **1**. The NOESY correlations of H-6*β* (δ 2.44)/H-5 (δ 2.74)/H-9*β* (δ 2.22) and H-5/H-1′ (δ 3.17) confirmed those protons located on the *β*-face. On the contrary, the NOESY correlations of H-9*α* (δ 1.80)/H_3_-14/H-6*α* (δ 2.64) suggested they are on the *α*-face. Therefore, the configuration of **2** was unambiguously determined.

Clasamane C (**3**) possessed a molecular formula of C_18_H_22_O_5_, which is consistent with its positive sodiated HRESIMS ion at *m*/*z* 341.1362. The ^1^H and ^13^C NMR spectroscopic data of **3** were similar to those of **10**, suggesting they are congeners. Comparison of the NMR spectra between **3** and **10** showed that **3** has an additional methoxy group (δ_H_ 3.07 (s) (H_3_-1′); δ_C_ 50.3 (C-1′)). This methoxy group situated at C-8 was evidenced by the HMBC correlation from H_3_-1′ to C-8 (δ 105.7). The NOESY correlation between H-5 (δ 1.86) and H_3_-14 (δ 1.56) revealed they were on the same face (*α*-orientation) of the molecule. The absolute configuration of **3** was determined by ECD data analysis. Due to the consistency of ECD curves between **3** and **11** (Figure S3), the absolute configuration of **3** was defined as 5*R*,8*S*,10*R*.

Clasamane D (**4**) was isolated as a colorless oil and had the molecular formula of C_19_H_24_O_5_ inferred from the sodiated HRESIMS ion at *m*/*z* 355.1518, which is 14 amu more than **3**. The UV, IR and NMR data of **4** were quite similar to those of **3**, except the methoxy group in **3** was replaced by an ethoxy group (δ_H_ 3.38 (m), 3.09 (m) (H_2_-1′); δ_C_ 58.7 (C-1′); δ_H_ 1.14 (t) (H_3_-2′); δ_C_ 15.1 (C-2′)) in **4**. This speculation is consistent with the difference in mass spectrometry between **4** and **3**, and it was confirmed by the HMBC correlation from H_2_-1′ to C-8 (δ 105.7). The similar ECD trends of **4** and **3** suggested these two compounds share the same absolute configuration. Thus, the structure of **4** was determined as shown.

**Figure 3:**
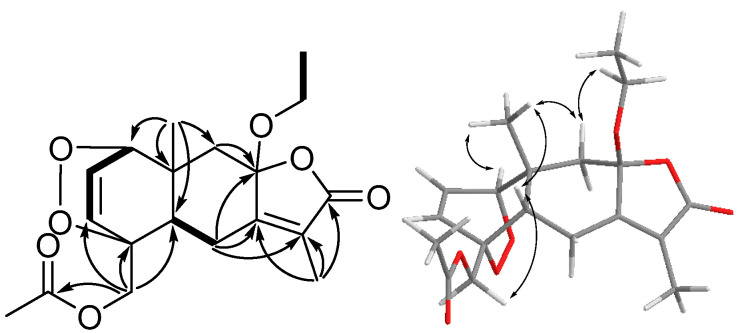
Key COSY (bold), HMBC (arrow), NOESY (double arrow) correlations and ECD spectra of **5**.

Clasamane E (**5**) was a colorless oil, and its HRESIMS data showed a [M + Na]^+^ ion at *m*/*z* 387.1414, suggesting the molecular formula of C_19_H_24_O_7_ with seven indices of hydrogen deficiency. The ^1^H and ^13^C data (Table 1 and Table 2) of **5** revealed typical signals of ethoxy (δ_H_ 3.46 (m), 3.15 (m) (H_2_-1′); δ_C_ 59.1 (C-1′); δ_H_ 1.21 (t) (H_3_-2′); δ_C_ 15.1 (C-2′)) and acetoxy (δ_C_ 170.7 (C-1″); δ_H_ 2.17 (s) (H_3_-2″); δ_C_ 20.8 (C-2″)) groups. A detailed analysis of COSY and HMBC spectra (Figure 3) established the planar structure of **5**. In the COSY spectrum, proton spin systems of H-1 (δ 4.20)/H-2 (δ 6.74)/H-3 (δ 6.39) and H-5 (δ 2.53)/H_2_-6 (δ 2.61 and δ 2.47) were found. These two proton spin systems and the HMBC correlations from H_3_-14 (δ 1.61) to C-1 (δ 79.5), C-5 (δ 42.5), C-9 (δ 43.8), and C-10 (δ 38.7), from H_2_-15 (δ 4.43 and δ 4.37) to C-3 (δ 129.2), C-4 (δ 79.8), and C-5, from H_2_-9 (δ 2.26 and δ 1.40) to C-8 (δ 105.3), from H_2_-6 to C-7 (δ 155.8), C-8, and C-11 (δ 124.2), and from H_3_-13 (δ 1.79) to C-7, C-11, and C-12 (δ 171.0) can be used to assemble the framework of eudensamane-type sesquiterpene lactone. The HMBC correlations from H_2_-1′ to C-8 and from H_2_-15 to C-1″ indicated the connection of ethoxy and acetoxy groups, respectively. The above findings accounted for six of the seven indices of hydrogen deficiency, which implied an additional ring should exist in **5**. Considering the molecular formula, two oxygen atoms were not assigned yet, and the carbon chemical shift of C-1 and C-4 suggested that these two carbons are oxygen bearing. Thus, a peroxide bridge between C-1 and C-4 was allocated. This assignment was also confirmed by the down-field shifted signals of H-2 and H-3 [17]. The *cis*-decalin moiety of **5** was assured by the NOESY correlations between H_3_-14 and H-5, which were assigned on the α-face. In addition, the NOESY correlations of H_2_-15/H-5 and H_3_-14/H-1 revealed the peroxide bridge was on the *β*-face of the molecule. The NOESY correlation of H-9*β* (δ 2.26)/H_2_-1″ indicated the *β*-orientation of the ethoxy group. Therefore, the stereocenters of **5** could be temporarily assigned as 1S*,4R*,5S*,8S*,10R* or 1R*,4S*,5R*,8S*,10S* (Figure S45). The ^1^H and ^13^C data of those two isomers were calculated by Gaussian 16, and the data were applied to DP4+ probability analysis. The analytic result indicated that the 1S*,4R*,5S*,8S*,10R* isomer has 100% possibility, so the configuration of **5** was determined.

Clabellane A (**6**) was obtained as a colorless oil with the molecular formula C_20_H_33_BrO_4_ and four degrees of unsaturation based on the HRESIMS ion at *m*/*z* 439.1455 [M + Na]^+^. The presence of one bromine atom was confirmed by the equal intensity between [M + Na]^+^ and [M + 2 + Na]^+^ in the mass spectrum. The presence of hydroxy functionality was confirmed by the IR absorption at 3432 cm^−1^. The ^1^H NMR data (Table 3) of **6** demonstrated proton signals of three methyls (δ 1.27 (s) (H_3_-20), δ 1.21 (s) (H_3_-19), and δ 0.86 (s) (H_3_-15)), an oxymethylene (δ 3.87 (d, *J* = 11.3), δ 3.65 (d, *J* = 11.3) (H_2_-17)), and an exomethylene (δ 5.03 (s), δ 4.84 (s) (H_2_-16)). The twenty carbon signals of **6** could be clearly separated into one exocyclic C=C double bond (δ 113.8 (C-16) and δ 147.4 (C-4)), one oxymethylene (δ 67.0 (C-17)), three oxygen-bearing quaternary carbons (δ 76.2 (C-11), δ 75.4 (C-18), and δ 75.0 (C-8)), one quaternary carbon (δ 44.6 (C-1)), seven methylenes (δ 42.4 (C-2), δ 38.8 (C-14), δ 35.4 (C-5), δ 33.3 (C-9), δ 29.7 (C-6), δ 27.7 (C-13), and δ 25.1 (C-3)), three methines (δ 63.0 (C-7), δ 54.7 (C-10) and δ 50.2 (C-12)), and three methyls (δ 29.6 (C-19), δ 26.0 (C-20) and δ 24.1 (C-15)) by using ^13^C NMR (Table 2) data together with DEPT-135 and HSQC spectra. Considering the above data and the reported compounds isolated from the genus *Clavularia*, **6** can be deduced as a dollabellane-type diterpenoid.

**Figure 4:**
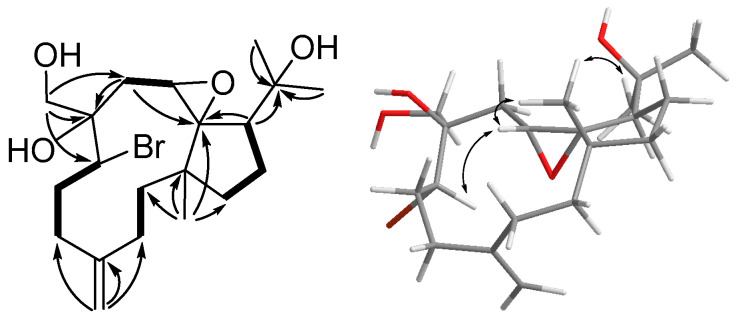
Key COSY (bold), HMBC (arrow), and NOESY (double arrow) correlations of **6**.

The planar structure of **6** was established by COSY and HMBC correlations (Figure 4). Three proton sequences of H_2_-2 (δ 1.96 and δ 1.25)/H_2_-3 (δ 2.11 and δ 1.63), H_2_-5 (δ 2.42 and δ 2.28)/H_2_-6 (δ 2.11 and δ 1.90)/H-7 (δ 4.04), and H_2_-9 (δ 2.25)/H-10 (δ 2.86) were observed from the COSY spectrum. Those findings and the HMBC correlation from H_2_-16 to C-3, C-4, and C-5; from H_2_-17 to C-7, C-8, and C-9; from H_2_-9 to C-8 and C-11; and from H_3_-15 to C-1, C-2, and C-11 could build the cycloundecane moiety (C-1 to C-11) of **6**, and it could confirm the presence of exomethylene (H_2_-16) connecting at C-4, an oxymethylene attaching at C-8, and a methyl (H_3_-15) connecting at C-1. In addition, the COSY correlations of H-12 (δ 2.21)/H_2_-13 (δ 1.90 and δ 1.63)/H_2_-14 (δ 1.76) together with the HMBC correlations from H-12 to C-11 and from H_3_-15 to C-1, C-11, and C-14 could establish the cyclopentane ring of **6**. The HMBC correlations from H-12 and the geminal methyls (H_3_-19 and H_3_-20) to C-3 constructed the isopropyl alcohol group. The planar structure of **6** was found to be similar to that of clavinflol B (**13**) except that the chlorine atom in the molecular formula (C_20_H_33_BrO_4_) of **13** was replaced by a bromine atom in **6**. The halogen atom of **6** was allocated at C-7, the same as that of **13**, due to the downfield shifted carbon chemical shift at this position (δ 66.2 in **13** and δ 63.0 in **6**). The relative configuration of **6** was determined through the NOESY spectrum (Figure 4). The bromine atom was assigned on the *α*-orientation to avoid steric interaction, while the NOESY correlations of H-7/H-10/H_3_-15/H_3_-19 indicated those protons were on the *β*–face of the molecule. On the other hand, the NOESY correlation between H_2_-17 and H_2_-9 implied that the hydroxy group should be *β*-oriented. Therefore, the configuration of **6** could be defined as 1R*,7R*,10S*,12R*. The configuration of C-8 was deduced to be 8*R* by the DP4+ probability measurement. The 1R*,7R*,8R*,10S*,12R* isomer showed an overwhelming possibility (100%) compared with the 1R*,7R*,8S*,10S*,12R* isomer (0%), which confirmed the configuration of **6**.

Clabellane B (**7**) was found to possess the molecular formula C_20_H_33_IO_4_ according to the sodium adduct ion at *m*/*z* 487.1318 (calcd for 487.1316) in the HRESIMS. The UV, IR, and NMR data (Figure 2 and Figure 3) were quite similar to that of **6**, indicating they are close analogs. The major difference between **7** and **6** was found in the molecular formula; the bromine atom in **6** was replaced by an iodine atom in 7. The heavy atom effect of C-7 (δ 46.0 in **7** and δ 63.0 in **6**) revealed that the bromine atom at C-7 in **6** was replaced by an iodine atom in **7**. Since the specific rotation, ECD, and NOESY data of **7** also resembled those of **6**, and the stereochemistry of **7** was thus assigned identically.

**Figure 5:**
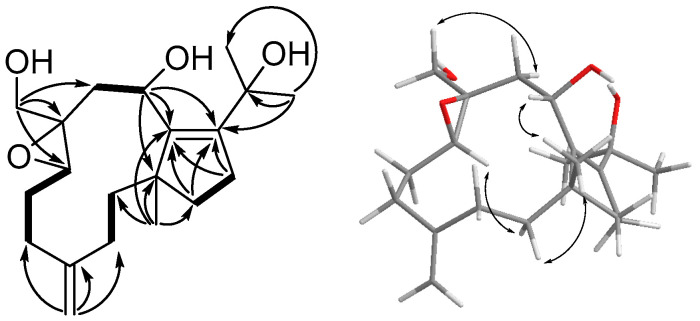
Key COSY (bold), HMBC (arrow), and NOESY (double arrow) correlations of **8**.

Clabellane C (**8**) has the molecular formula C_20_H_32_O_4_ (IHD = 5), as deduced from HRESIMS and NMR spectrometric data. The IR spectrum of **8** indicated the presence of hydroxy (3378 cm^−1^) and exomethylene (1643 cm^−1^) functionalities. The ^1^H and ^13^C NMR data of **8** were analogous to those of **6**, suggesting it is also a dolabellane-type diterpenoid. Four proton sequences of H_2_-2 (δ 1.74 and δ 1.32)/H_2_-3 (δ 2.04 and δ 1.79), H_2_-5 (δ 2.46 and δ 2.27)/H_2_-6 (δ 1.96 and δ 1.49)/H-7 (δ 3.13), H_2_-9 (δ 2.58 and δ 1.96)/H-10 (δ 4.37), and H_2_-13 (δ 2.35 and δ 2.20)/H_2_-14 (δ 1.74 and δ 1.43) were observed by the cross-peaks in the COSY spectrum. Moreover, the above finding and the HMBC correlations from H_2_-16 (δ 4.71 and δ 4.67) to C-3 (δ 29.9), C-4 (δ 150.3), and C-5 (δ 28.7); from H_2_-17 (δ 3.98 and δ 3.32) to C-7 (δ 60.2), C-8 (δ 63.8), and C-9 (δ 37.8); from H-10 to C-1 (δ 53.1), C-11 (δ 141.2), and C-12 (δ 144.7); from H_3_-15 (δ 1.05) to C-1 (δ 53.1), C-2 (δ 35.7), C-11, and C-14 (δ 32.7); and from H_3_-19 (δ 1.35) to C-12, C-18 (δ 72.3), and C-20 (δ 29.7) could establish the planar structure of **8**. Three hydroxy groups allocated at C-10 (δ 65.0), C-17 (δ 68.6), and C-18 were assured by virtue of their downfield shifted carbon chemical shifts. The aforementioned data accounted for four of the five indices of hydrogen deficiency, suggesting an additional ring remained in **8**. An epoxy group was assigned to C-7 and C-8 by their downfield shifted carbon chemical shifts. Therefore, the planar structure was established. The relative configuration of **8** was determined by interpretation of NOESY data (Figure 5) and DP4+ probability analysis. The NOESY correlation of H-10/H_3_-15/H-2*β* (δ 1.74) indicated the β-orientation of these protons. The presence of NOESY cross-peaks between H-7 and H-2*α* (δ 1.32) indicated they are *α*-orientated. The configuration assignment was confirmed by the 100% possibility of DP4+ analysis. On the basis of the data described above, the configuration of **8** was established to be 1S*,7S*,8R*,10S*.

In our earlier investigation, we found that the methanol extract of *Clavularia* spp. had an apoptotic effect on oral cancer cells [10]. Hence, most of the isolated compounds were evaluated in vitro for their antiproliferative effect against oral cancer cells (Ca9-22) using cellular ATP assay. As shown in Table S22, new iodinated dolabellane **7** exhibited strong cytotoxic effects with an IC_50_ value of 15.7 μM, while the eudensamane-type sesquiterpenes were less active. It is noted that the cytotoxic effect of compound **15** (IC_50_ = 24.9 μM) was seven times higher than that of **16** (IC_50_ = 166.7 μM), which implied the position of the C=C bond might change the bioactivity dramatically. For the halogenated dolabellanes **6**, **7**, and **13**, the iodinated one showed the best cytotoxic activity and the chlorinated one was the weakest. Moreover, clasamane E (**5**) having a peroxide bridge showed a relatively good cytotoxic activity against the Ca9-22 cell among all isolated eudensamane-type sesquiterpene lactones.

## 3. Materials and Methods

### 3.1. General

Merck KGaA (Darmstadt, Germany) cellite 545 (0.02–0.1 mm) and silica gel 60 (0.015–0.040 mm) were used for dry sample and flash column chromatography, respectively. Phenomenex (Torrance, CA, USA) C_18_, phenyl-hexyl, and biphenyl columns were used for high-performance liquid chromatography (HPLC). The Shimadzu (Kyoto, Japan) HPLC instrument consisted of an LC-40D solvent delivery module, DGU-405 degassing unit, CBM-40 system controller, CTO-40S column oven, SPD-M40 photo diode array detector, and FRC-10A fraction collector. A Jasco (Tokyo, Japan) V-650 spectrophotometer was used for measuring UV data. A Jasco FT/IR-4X spectrophotometer was chosen for measuring IR data. A Jasco J-815 CD spectrometer was used for recording the circular dichroism data. Specific optical rotation was measured by a Jasco P-2000 polarimeter. NMR spectra were obtained from Varian (Palo Alto, CA, USA) Mercury Plus 400 MHz and VNMRS 600 MHz FT-NMR spectrometers. A Bruker (Bremen, Germany) APEX II spectrometer was used for detecting HRSIMS.

### 3.2. Animal Material

The coral materials were collected in May 2021 off the coast of Green Island, Taiwan. Coral specimens were identified as *Clavularia* spp. by Dr. Yuan-Bin Cheng. A voucher specimen (code: CI2021) was given, and the specimens were deposited at the Department of Marine Biotechnology and Resources, National Sun Yat-sen University, Kaohsiung, Taiwan. It is noted that the coral materials were previously identified as Clavularia inflata [10]. However, the materials contained more than one species and can only be recognized as *Clavularia* spp.

### 3.3. Extraction and Isolation

Coral materials were lyophilized and immersed in EtOH at room temperature for three days (thrice) to provide an EtOH extract (124.6 g). This extract was partitioned between EtOAc and H_2_O. The EtOAc soluble extract (34.8 g) was further partitioned with hexanes and 75% MeOH. The 75% MeOH layer (11.1 g) was first separated by a silica gel flash column and stepwise eluted with hexanes/EtOAc/MeOH (8/1/0 to 0/0/1) to give nine fractions (A–I). Further silica gel column chromatography of fraction B (622.8 mg), stepwise eluting from hexanes/acetone (40/1) to pure acetone, yielded nine subfractions (B1–B9). Subfraction B2 (55.8 mg) was purified by RP-HPLC (C_18_ column) using 80% MeOH as eluent to give compounds **1** (3.3 mg) and **2** (1.9 mg). Subfraction B6 (373.7 mg) was isolated by a silica gel open column stepwise eluted with hexanes/EtOAc/MeOH (100/10/1 to 0/0/1) to give fractions B6A–B6D and compound **10** (4.2 mg). Fraction B6B (22.2 mg) was separated by RP-HPLC (phenyl hexyl column) eluting with 80% MeOH to yield compound **15** (8.0 mg). Fraction B6C (307.1 mg) was repurified by RP-HPLC (C_18_ column) eluting with MeOH/H_2_O (3/2 to 0/1), and compounds **17** (7.9 mg) and **18** (3.7 mg) were obtained. Fraction C (494.7 mg) was fractionated over a silica gel open column stepwise eluted with hexanes/CH_2_Cl_2_/acetone (80/20/0 to 0/0/1) to afford subfractions (C1–C7). Subfraction C4 (104.4 mg) was repurified by a silica gel open column and stepwise eluted by hexanes/EtOAC (15/1 to 0/1) to give fractions C4A–C4F. The RP-HPLC (phenyl hexyl column) separation of fraction C4D (29.9 mg) eluting with 65% MeOH produced compounds **3** (0.6 mg), **4** (5.8 mg), and **5** (1.0 mg). Subfraction C5 (95.0 mg) was applied to a silica gel open column stepwise eluted by hexanes/acetone (15/1 to 0/1) to give five fractions (C5A–C5E). Fraction C5A (18.1 mg) was purified by RP-HPLC (phenyl hexyl column) eluting with 75% MeOH to give compound **19** (5.6 mg). Fraction C5B (20.4 mg) was successively isolated by RP-HPLC (phenyl hexyl column) eluting with 75% MeOH to yield compound **16** (6.0 mg). Subfraction C6 (136.1 mg) was chromatographed on a silica gel column (stepwise eluted by hexanes/acetone/MeOH 6/1/0 to 0/0/1) to obtain fractions C6A–C6C. Fraction C6B (95.0 mg) was repeatedly purified by RP-HPLC (C_18_ column) with decreasing polarity of MeOH to yield compound **9** (1.6 mg). Fraction D (2.7 g) was further fractionalized into six subfractions (D1–D6) by using a silica gel column stepwise eluted with hexanes/EtOAc/MeOH (100/10/1 to 0/0/1). Subfraction D3 (702.8 mg) was isolated by a silica gel column stepwise eluted with CH_2_Cl_2_/acetone/MeOH (120/1/0 to 0/0/1), and a diterpenoid-enriched fraction D3A was obtained. Fraction D3A (278.1 mg) was repurified by a silica gel column stepwise eluted with hexanes/acetone/MeOH (20/1/0 to 0/0/1) to afford compound **22** (100.6 mg). Subfraction D4 (986.2 mg) was fractionated by a silica gel column with a gradient of CH_2_Cl_2_/acetone/MeOH (120/1/0 to 0/0/1) to give six fractions (D4A–D4F). Fraction D4F (395.2 mg) was subjected to a C_18_ column stepwise eluted with MeOH/H_2_O (20/80 to 1/0) to yield eight fractions D4F1–D4F8. Compound **11** (4.1 mg) was purified from fraction D4F4 (11.0 mg) by RP-HPLC (phenyl hexyl column) eluted with MeCN/H_2_O (45/55). Fraction D4F7 (99.8 mg) was repurified by a PR-HPLC (C_18_ column) with a gradient of MeCN/H_2_O (40/60 to 55/45) to afford compounds **12** (1.1 mg) and **14** (17.1 mg). Subfraction D5 (731.5 mg) was separated by a silica gel column and eluted with a gradient of hexanes/CH_2_Cl_2_/MeOH (80/10/1 to 0/0/1) to yield fraction D5A (382.4 mg). Fraction D5A was further separated by another silica gel column stepwise eluted with hexanes/acetone/MeOH (10/1/0 to 0/0/1), and the fraction D5A3 was obtained. Fraction D5A3 (89.9 mg) was then subjected to a C_18_ column stepwise eluted with MeOH/H_2_O (3/7 to 0/1) to give fraction D5A3F. Compound **20** (0.6 mg) was finally isolated from fraction D5A3F (22.3 mg) by RP-HPLC (biphenyl column) with a gradient of MeCN/H_2_O (40/60 to 55/45). Subfraction E (1.1 g) was divided into seven fractions (E1–E7) by a silica gel column stepwise eluted with hexanes/CH_2_Cl_2_/MeOH (15/1/0 to 0/0/1). Fraction E2 (308.3 mg) was separated by another silica gel column stepwise eluted with hexanes/acetone/MeOH (15/1/0 to 0/0/1), and eight fractions (E2A–E2H) were produced. Fraction E2E (82.8 mg) was applied to RP-HPLC (C_18_ column) eluted with MeCN/H_2_O using a gradient from 25/75 to 60/40, resulting in the isolation of **7** (4.1 mg), **21** (43.0 mg) and a subfraction E2E10. Compounds **6** (0.8 mg) and **8** (0.9 mg) were purified from subfraction E2E10 (4.3 mg) by RP-HPLC (biphenyl column) using MeCN/H_2_O (45/55) as eluent. Fraction E2F (73.3 mg) was separated by RP-HPLC (C_18_ column) eluted with MeCN/H_2_O (55/45) to yield compound **13** (26.2 mg) and the subfraction E2F4. Subfraction E2F4 (5.4 mg) was repurified by RP-HPLC (biphenyl column) eluted with MeCN/H_2_O (35/65) to give compound **23** (1.4 mg).

### 3.4. Spectroscopic Data

Clasamane A (**1**): colorless oil; ${\left[\alpha \right]}_{D}^{25}$ +62 (c 0.05, MeOH); UV λmax (log ε) 282 (3.58), 218 (2.91) nm; IR (neat) v_max_ 2926, 1751, 1445, 1386, 1311, 1293, 1236, 1174, 1082, 1035 cm^−1^; ECD λmax(Δε) 262 (+1.62), 225 (+161) nm; ^1^H and ^13^C NMR data are presented in Table 1; HRESIMS *m*/*z* 299.1616 [M + Na]^+^ (calcd for C_17_H_24_NaO_3_, 299.1618).

Clasamane B (**2**): colorless oil; ${\left[\alpha \right]}_{D}^{25}$ −9 (c 0.05, MeOH); IR (neat) v_max_ 2925, 1752, 1552, 1441, 1383, 1294, 1235, 1168, 1114, 1043 cm^−1^; ECD λmax(Δε) 248 (−0.60), 214 (−1.26) nm; ^1^H and ^13^C NMR data are presented in Table 1 and Table 2; HRESIMS *m*/*z* 299.1616 [M + Na]^+^ (calcd for C_17_H_24_NaO_3_, 299.1618).

Clasamane C (**3**): colorless oil; ${\left[\alpha \right]}_{D}^{25}$ −315 (c 0.04, MeOH); UV λmax (log ε) 282 (3.24), 214 (3.74) nm; IR (neat) v_max_ 2925, 2858, 1758, 1448, 1373, 1228, 1175, 1125, 1083, 1025 cm^−1^; ECD λmax(Δε) 274 (−4.58), 234 (+1.60), 209 (−1.59) nm; ^1^H and ^13^C NMR data are presented in Table 1 and Table 2; HRESIMS *m*/*z* 341.1362 [M + Na]^+^ (calcd for C_18_H_22_NaO_5_, 341.1359).

Clasamane D (**4**): colorless oil; ${\left[\alpha \right]}_{D}^{25}$ −263 (c 0.05, MeOH); UV λmax (log ε) 277 (3.55), 216 (3.95) nm; IR (neat) v_max_ 2975, 2928, 1763, 1443, 1378, 1320, 1233, 1175, 1088, 1026 cm^−1^; ECD λmax(Δε) 274 (−10.61), 234 (+3.04), 211 (−3.36) nm; ^1^H and ^13^C NMR data are presented in Table 1 and Table 2; HRESIMS *m*/*z* 355.1518 [M + Na]^+^ (calcd for C_19_H_24_NaO_5_, 355.1516).

Clasamane E (**5**): colorless oil; ${\left[\alpha \right]}_{D}^{25}$ +14 (c 0.05, MeOH); UV λmax (log ε) 217 (3.72) nm; IR (neat) v_max_ 2928, 1758, 1448, 1378, 1295, 1237, 1171, 1113, 1042 cm^−1^; ECD λmax(Δε) 290 (−0.08), 246 (+1.24), 207 (−1.81) nm; ^1^H and ^13^C NMR data are presented in Table 1 and Table 2; HRESIMS *m*/*z* 387.1414 [M + Na]^+^ (calcd for C_19_H_24_NaO_7_, 387.1414).

Clabellane A (**6**): colorless oil; ${\left[\alpha \right]}_{D}^{25}$ +36 (c 0.05, MeOH); UV λmax (log ε) 204 (3.85) nm; IR (neat) v_max_ 3432, 2966, 1725, 1646, 1447, 1383, 1252, 1169, 1131 cm^−1^; ECD λmax(Δε) 204 (+1.38) nm; ^1^H and ^13^C NMR data are presented in Table 2 and Table 3; HRESIMS *m*/*z* 439.1455 [M + Na]^+^ (calcd for C_20_H_33_BrNaO_4_, 439.1454).

Clabellane B (**7**): colorless oil; ${\left[\alpha \right]}_{D}^{25}$ +5 (c 0.05, MeOH); UV λmax (log ε) 204 (3.97) nm; IR (neat) v_max_ 3412, 2963, 2930, 1726, 1645, 1451, 1381, 1243, 1171, 1126, 1045 cm^−1^; ECD λmax(Δε) 208 (+1.68) nm; ^1^H and ^13^C NMR data are presented in Table 2 and Table 3; HRESIMS *m*/*z* 487.1318 [M + Na]^+^ (calcd for C_20_H_33_INaO_4_, 487.1316).

Clabellane C (**8**): colorless oil; ${\left[\alpha \right]}_{D}^{25}$ +50 (c 0.05, MeOH); UV λmax (log ε) 206 (3.85) nm; IR (neat) v_max_ 3412, 2963, 2930, 1726, 1645, 1451, 1381, 1243, 1171, 1126, 1045 cm^−1^; ECD λmax(Δε) 203 (−2.81) nm; ^1^H and ^13^C NMR data are presented in Table 2 and Table 3; HRESIMS *m*/*z* 359.2193 [M + Na]^+^ (calcd for C_20_H_32_NaO_4_, 359.2193).

### 3.5. Cytotoxicity Assays

The cell viability (IC_50_) at 72 h of oral cancer Ca9-22 cells (HSRRB, Ibaraki, Osaka, Japan) [18] was assessed by an ATP detection kit (PerkinElmer Life Sciences, Boston, MA, USA) [19,20] and measured by a luminometer (Berthold Technologies GmbH & Co., Bad Wildbad, Germany). The data are provided as means ± SD in three independent experiments.

## 4. Conclusions

Although the natural product investigation of octacoral *Clavularia* spp. started last century [3], new marine natural products have been successively identified from this genus to date [5,21,22]. In the current study, 23 marine natural products, including eight new compounds, were identified. In view of chemical structure, the isolates can be divided into eudensamane-type sesquiterpene lactones (**1**–**5** and **9**–**12**) and dolabellane-type diterpenes (**6**–**8** and **13**–**22**). In terms of bioactivity, the dolabellane-type diterpenes demonstrate better cytotoxic activity than eudensamane-type sesquiterpene lactones. Our findings support our previous investigation and prove that the marine soft coral of the genus *Clavularia* is a rich source for the identification of cytotoxic compounds.

## Figures and Tables

**Table 1:** ^1^H NMR (600 MHz) spectroscopic data of compounds **1**–**5** (*δ* in ppm, *J* value in Hz) *^a^*.

No.	1	2	3	4 *^b^*	5
1	1.23, dd (12.9, 5.9)	1.48, m	5.78, d (9.6)	5.83, d (3.1)	4.20, d (6.0)
	1.56, d (12.9)	1.63, m			
2	1.63, m	1.48, m	5.85, d (9.6, 5.2)	5.83, d (3.1)	6.74, dd (8.4, 6.0)
		1.63, m			
3	1.96, m	2.06, m	5.95, d (5.2)	5.95, m	6.39, d (8.4)
	2.36, d (13.7)	2.38, m			
5	1.82, dd (12.8, 1.3)	2.74, m	1.86, dd (13.0, 4.2)	1.85, dd (12.5, 4.2)	2.53, dd (10.8, 2.9)
6	2.27, t (12.8)	2.44, m	2.17, t (13.0)	2.18, t (12.5)	2.61, m
	2.62, dd (12.8, 3.2)	2.64, dd (11.8, 8.2)	2.68, dd (13.0, 4.2)	2.67, dd (12.5, 4.2)	2.47, dd (16.4, 2.9)
9	1.41, d (13.7)	1.80, d (14.5)	1.55, d (14.2)	1.52, d (13.8)	1.40, d (13.9)
	2.36, d (13.7)	2.22, d (14.5)	2.52, d (14.2)	2.53, d (13.8)	2.26, d (13.9)
13	1.86, s	1.88, s	1.88, s	1.86, s	1.79, s
14	0.99, s	0.64, s	0.97, s	0.96, s	1.61, s
15	4.58, s	4.64, s	4.60, d (13.1)	4.59, d (13.0)	4.37, d (13.1)
		4.89, s	4.67, d (13.1)	4.66, d (13.0)	4.43, d (13.1)
1′	3.28, m	3.17, m	3.07, s	3.09, m	3.15, m
	3.46, m	3.41, m		3.38, m	3.46, m
2′	1.18, t (7.0)	1.18, t (7.0)		1.14, t (7.0)	1.21, t (7.0)
2″			2.11, s	2.10, s	2.17, s

*^a^* Measured in CDCl3. *^b^* Measured at 400 MHz in CDCl3.

**Table 2:** ^13^C NMR (150 MHz) spectroscopic data of compounds **1**–**8** (*δ* in ppm) *^a^*.

No.	1	2	3	4 *^b^*	5	6	7 *^b^*	8
1	41.4 CH_2_	42.6 CH_2_	137.4 CH	137.6 CH	79.5 CH	44.6 C	44.6 C	53.1 C
2	22.3 CH_2_	23.2 CH_2_	120.2 CH	119.9 CH	135.8 CH	42.4 CH_2_	42.3 CH_2_	35.7 CH_2_
3	36.0 CH_2_	36.5 CH_2_	121.7 CH	121.7 CH	129.2 CH	25.1 CH_2_	25.0 CH_2_	29.9 CH2
4	148.7 C	148.6 C	133.6 C	133.5 C	79.8 C	147.4 C	147.1 C	150.3 C
5	51.8 CH	41.6 CH	44.9 CH	44.9 CH	42.5 CH	35.4 CH_2_	36.4 CH_2_	28.7 CH_2_
6	25.0 CH_2_	24.2 CH_2_	25.7 CH_2_	25.8 CH_2_	22.8 CH_2_	29.7 CH_2_	30.9 CH_2_	22.3 CH_2_
7	159.9 C	157.4 C	156.8 C	157.2 C	155.8 C	63.0 CH	46.0 CH	60.2 CH
8	106.2 C	106.9 C	105.7 C	105.7 C	105.3 C	75.0 C	75.1 C	63.8 C
9	50.2 CH_2_	50.7 CH_2_	48.6 CH_2_	48.8 CH_2_	43.8 CH_2_	33.3 CH_2_	32.1 CH_2_	37.8 CH_2_
10	36.8 C	34.9 C	36.5 C	36.5 C	38.7 C	54.7 CH	54.7 CH	65.0 CH
11	123.8 C	125.5 C	123.8 C	123.5 C	124.2 C	76.2 C	76.1 C	141.2 C
12	171.9 C	171.9 C	171.5 C	171.6 C	171.1 C	50.2 CH	50.2 CH	144.7 C
13	8.3 CH_3_	8.3 CH_3_	8.3 CH_3_	8.3 CH_3_	8.3 CH_3_	27.7 CH_2_	27.6 CH_2_	32.8 CH_2_
14	16.4 CH_3_	21.0 CH_3_	25.1 CH_3_	25.1 CH_3_	29.2 CH_3_	38.8 CH_2_	38.8 CH_2_	32.7 CH_2_
15	106.7 CH_2_	107.4 CH_2_	66.3 CH_2_	66.3 CH_2_	62.8 CH_2_	24.1 CH_3_	24.1 CH_3_	26.8 CH3
16						113.8 CH_2_	113.7 CH_2_	111.1 CH_2_
17						67.0 CH_2_	69.5 CH_2_	68.6 CH_2_
18						75.4 C	75.2 C	72.3 C
19						29.6 CH_3_	29.5 CH_3_	31.0 CH_3_
20						26.0 CH_3_	26.0 CH_3_	29.7 CH_3_
1′	58.7 CH_2_	58.8 CH_2_	50.3 CH_3_	50.3 CH_3_	59.1 CH_3_			
2′	15.2 CH_3_	15.3 CH_3_			15.1 CH_3_			
1″			170.8 C	170.8 C	170.7 C			
2″			20.8 CH_3_	20.8 CH_3_	20.8 CH_3_			

*^a^* Measured at 150 MHz in CDCl_3_. *^b^* Measured at 100 MHz in CDCl_3_.

**Table 3:** ^1^H NMR (600 MHz) spectroscopic data of compounds **6**–**8** (*δ* in ppm, *J* value in Hz) *^a^*.

No.	6	7 *^b^*	8
2	1.96, m	1.95, m	1.74, m
	1.25, m	1.24, m	1.32, dd (7.3, 2.0)
3	2.11, m	2.10, m	2.04, m
	1.63, m	1.56, m	1.79, d (8.8)
5	2.42, td (8.9, 4.3)	2.37, m	2.46, m
	2.28, m	2.25, m	2.27, m
6	1.90, m	1.87, m	1.96, m
	2.11, m		1.49, m
7	4.04, d (11.8)	4.02, dd (7.6, 6.2)	3.13, t (6.7)
9	2.25, d (3.7)	2.22, m	2.58, dd (16.0, 8.0)
		2.32, m	1.96, m
10	2.89, d (6.1)	2.89, d (6.1)	4.37, d (8.0)
12	2.21, d (10.3)	2.22, m	
13	1.90, m	1.90, m	2.35, m
	1.63, m	1.61, m	2.20, ddd (10.5, 6.2, 1.4)
14	1.76, m	1.76, m	1.43, ddd (12.4, 8.3, 2.0)
			1.74, m
15	0.86, s	0.85, s	1.05, s
16	4.84, s	4.82, s	4.67, s
	5.03, s	5.07, s	4.71, s
17	3.87, d (11.3)	3.87, d (11.4)	3.98, d (11.7)
	3.65, d (11.3)	3.67, d (11.4)	3.32, d (11.7)
19	1.21, s	1.22, s	1.35, s
20	1.27, s	1.27, s	1.38, s

*^a^* Measured in CDCl_3_ *^b^* Measured at 400 MHz in CDCl_3_.

## Data Availability

Data are available in a publicly accessible repository.

## Supplementary Materials

The following supporting information can be downloaded at: https://www.mdpi.com/article/10.3390/md21100529/s1, Figure S1: Experimental ECD spectra of **1** and **9**; Figure S2: NOESY (double arrow) correlations of **2**; Figure S3: Experimental ECD spectra of **3** and **11**; Figures S4–S9: 1D and 2D NMR spectra of **1**; Figure S10: HRESIMS spectrum of **1**; Figure S11: UV spectrum of **1**; Figure S12: IR spectrum of **1**; Figures S13–S18: 1D and 2D NMR spectra of **2**; Figure S19: HRESIMS spectrum of **2**; Figure S20: UV spectrum of **2**; Figure S21: IR spectrum of **2**; Figures S22–S27: 1D and 2D NMR spectra of **3**; Figure S28: HRESIMS spectrum of **3**; Figure S29: UV spectrum of **3**; Figure S30: IR spectrum of **3**; Figures S31–S36: 1D and 2D NMR spectra of **4**; Figure S37: HRESIMS spectrum of **4**; Figure S38: UV spectrum of **4**; Figure S39: IR spectrum of **4**; Figures S40–S45: 1D and 2D NMR spectra of **5**; Figure S46: HRESIMS spectrum of **5**; Figure S47: UV spectrum of **5**; Figure S48: IR spectrum of **5**; Figures S49–S54: 1D and 2D NMR spectra of **6**; Figure S55: HRESIMS spectrum of **6**; Figure S56: UV spectrum of **6**; Figure S57: IR spectrum of **6**; Figures S58–S63: 1D and 2D NMR spectra of **7**; Figure S64: HRESIMS spectrum of **7**; Figure S65: UV spectrum of **7**; Figure S66: IR spectrum of **7**; Figures S67–S72: 1D and 2D NMR spectra of **8**; Figure S73: HRESIMS spectrum of **8**; Figure S74: UV spectrum of **8**; Figure S75: IR spectrum of **8**; Table S1: Energy analyses of 1*R*,4*S*,5*R*,8*S*,10*S*-**5** (seven conformers); Table S2: Cartesian coordinates of the low-energy re-optimized conformers of 1*R*,4*S*,5*R*,8*S*,10*S*-**5** calculated at B3LYP/6-31G(d,p) level of theory; Table S3: Energy analyses of 1*S*,4*R*,5*S*,8*S*,10*R* -**5** (eight conformers); Table S4: Cartesian coordinates of the low-energy re-optimized conformers of 1*S*,4*R*,5*S*,8*S*,10*R*-**5** calculated at B3LYP/6-31G(d,p) level of theory; Table S5: Experimental and calculated ^1^H NMR data for compound **5**; Experimental and calculated ^13^C NMR data for compound **5**; Table S7: DP4+ analyses of calculated and experimental NMR chemical shifts of 5 (unscaled). Isomer 1: 1*R*,4*S*,5*R*,8*S*,10*S*-**5**; Isomer 2: 1*S*,4*R*,5*S*,8*S*,10*R*-**5**; Table S8: Energy analyses of 1*R*,7*R*,8*R*,10*S*,12*R*-**6** (six conformers); Table S9: Cartesian coordinates of the low-energy re-optimized conformers of 1*R*,7*R*,8*R*,10*S*,12*R*-**6** calculated at B3LYP/6-31G(d,p) level of theory; Table S10: Energy analyses of 1*R*,7*R*,8*S*,10*S*,12*R*-**6** (eight conformers); Table S11: Cartesian coordinates of the low-energy re-optimized conformers of 1*R*,7*R*,8*S*,10*S*,12*R*-**6** calculated at B3LYP/6-31G(d,p) level of theory; Table S12: Experimental and calculated ^1^H NMR data for compound **6**; Table S13: Experimental and calculated ^13^C NMR data for compound **6**; Table S14: DP4+ analyses of calculated and experimental NMR chemical shifts of **6** (unscaled). Isomer 1: 1*R*,7*R*,8*S*,10*R*,12*R*-**6**; Isomer 2: 1*R*,7*R*,8*S*,10*S*,12*R*-**6**; Table S15: Energy analyses of 1*R*,7*R*,8*S*,10*R*-**8** (four conformers); Table S16: Cartesian coordinates of the low-energy re-optimized conformers of 1*R*,7*R*,8*S*,10*R*-**8** calculated at B3LYP/6-31G(d,p) level of theory; Table S17: Energy analyses of 1*S*,7*S*,8*R*,10*S*-8 (four conformers); Table S18: Cartesian coordinates of the low-energy re-optimized conformers of 1*S*,7*S*,8*R*,10*S*-**8** calculated at B3LYP/6-31G(d,p) level of theory; Table S19: Experimental and calculated ^1^H NMR data for compound **8**; Table S20: Experimental and calculated ^13^C NMR data for compound **8**; Table S21: DP4+ analyses of calculated and experimental NMR chemical shifts of **8** (unscaled). Isomer 1: 1*R*,7*R*,8*S*,10*R*-**8**; Isomer 2: 1*S*,7*S*,8*R*,10*S*-**8**; Table S22: Cytotoxicity of active compounds against Human Oral Cancer (Ca9-22).
