# Supplementary material for: Chemical Constituents from Soft Coral Clavularia spp. Demonstrate Antiproliferative Effects on Oral Cancer Cells
Source: Mar Drugs. 2023 Oct 8;21(10):529. doi: 10.3390/md21100529 (PMC10608145; doi:10.3390/md21100529)
Supplement: Supplementary file 1 [file marinedrugs-21-00529-s001.zip › marinedrugs-2639781-supplementary.pdf]

Supporting Information

Chemical Constituents from Soft Coral *Clavularia* spp. Demonstrate Antiproliferative Effects on Oral Cancer Cells

Ming-Ya Cheng, Ya-Ting Chuang, Hsueh-Wei Chang, Zheng-Yu Lin, Ching-Yeu Chen, and Yuan-Bin Cheng*

Corresponding Author

Yuan-Bin Cheng − Department of Marine Biotechnology and Resources, National Sun Yat-sen University, Kaohsiung 80424, Taiwan; orcid.org/0000-0001-6581-1320; E-mail: jmb@mail.nsysu.edu.tw; Tel: +886-7-5252-000-5212

Authors

Ming-Ya Cheng − Department of Marine Biotechnology and Resources, National Sun Yat-sen University, Kaohsiung 80424, Taiwan

Ya-Ting Chuang − Department of Biomedical Science and Environmental Biology, PhD program of Life Science, College of Life Science, Kaohsiung Medical University, Kaohsiung 80708, Taiwan

Hsueh-Wei Chang − Department of Biomedical Science and Environmental Biology, PhD program of Life Science, College of Life Science, Kaohsiung Medical University, Kaohsiung 80708, Taiwan; Center for Cancer Research, Kaohsiung Medical University, Kaohsiung 80708, Taiwan; Department of Medical Research, Kaohsiung Medical University Hospital, Kaohsiung 80708, Taiwan

Zheng-Yu Lin − Department of Marine Biotechnology and Resources, National Sun Yat-sen University, Kaohsiung 80424, Taiwan

Yu-Chi Lin − Department of Physical Therapy, Tzu-Hui Institute of Technology, Pingtung 92641, Taiwan

Content

Table S1 Energy analyses of 1*R*,4*S*,5*R*,8*S*,10*S*-**5** (seven conformers) 6

Table S2 Cartesian coordinates of the low-energy re-optimized conformers of 1*R*,4*S*,5*R*,8*S*,10*S*-**5** calculated at B3LYP/6-31G(d,p) level of theory. 8

Table S3 Energy analyses of 1*S*,4*R*,5*S*,8*S*-10*R*-**5** (eight conformers) 15

Table S4 Cartesian coordinates of the low-energy re-optimized conformers of 1*S*,4*R*,5*S*,8*S*,10*R*-**5** calculated at B3LYP/6-31G(d,p) level of theory. 17

Table S5 Experimental and calculated ^{1}H NMR data for compound **5**. 24

Table S6 Experimental and calculated ^{13}C NMR data for compound **5**. 25

Table S7 DP4+ analyses of calculated and experimental NMR chemical shifts of **5** (unscaled). Isomer 1: 1*R*,4*S*,5*R*,8*S*,10*S*-**5**; Isomer 2: 1*S*,4*R*,5*S*,8*S*,10*R*-**5** 26

Table S8 Energy analyses of 1*R*,7*R*,8*R*,10*S*,12*R*-**6** (six conformers) 27

Table S9 Cartesian coordinates of the low-energy re-optimized conformers of 1*R*,7*R*,8*R*,10*S*,12*R*-**6** calculated at B3LYP/6-31G(d,p) level of theory 29

Table S10 Energy analyses of 1*R*,7*R*,8*S*,10*S*,12*R*-**6** (eight conformers) 35

Table S11 Cartesian coordinates of the low-energy re-optimized conformers of 1*R*,7*R*,8*S*,10*S*,12*R*-**6** calculated at B3LYP/6-31G(d,p) level of theory. 37

Table S12 Experimental and calculated ^{1}H NMR data for compound **6** 45

Table S13 Experimental and calculated ^{13}C NMR data for compound **6** 46

Table S14 DP4+ analyses of calculated and experimental NMR chemical shifts of **6** (unscaled). Isomer 1: 1*R*,7*R*,8*S*,10*R*,12*R*-**6**; Isomer 2: 1*R*,7*R*,8*S*,10*S*,12*R*-**6** 47

Table S15 Energy analyses of 1*R*,7*R*,8*S*,10*R*-**8** (four conformers) 48

Table S16 Cartesian coordinates of the low-energy re-optimized conformers of 1*R*,7*R*,8*S*,10*R*-**8** calculated at B3LYP/6-31G(d,p) level of theory 49

Table S17 Energy analyses of 1*S*,7*S*,8*R*,10*S*-**8** (four conformers) 53

Table S18 Cartesian coordinates of the low-energy re-optimized conformers of 1*S*,7*S*,8*R*,10*S*-**8** calculated at B3LYP/6-31G(d,p) level of theory 54

Table S19 Experimental and calculated ^{1}H NMR data for compound **8** 58

Table S20 Experimental and calculated ^{13}C NMR data for compound **8** 59

Table S21 DP4+ analyses of calculated and experimental NMR chemical shifts of **8** (unscaled). Isomer 1: 1*R*,7*R*,8*S*,10*R*-**8**; Isomer 2: 1*S*,7*S*,8*R*,10*S*-**8** 60

Table S22 Cytotoxicity of active compounds against Human Oral Cancer (Ca9-22)....61

Figure S1 Experimental ECD spectra of **1** and **9** 62
Figure S2 NOESY (double arrow) correlations of **2**....62
Figure S3 Experimental ECD spectra of **3** and **11**....62
Figure S4 ^{1}H NMR spectrum of **1** (600 MHz, $CDCl_3$) 63
Figure S5 ^{13}C NMR spectrum of **1** (125 MHz, $CDCl_3$) 63
Figure S6 COSY spectrum of **1**....64
Figure S7 HSQC spectrum of **1** 64
Figure S8 HMBC spectrum of **1** 65
Figure S9 NOESY spectrum of **1**....65
Figure S10 HRESIMS spectrum of **1**....66
Figure S11 UV spectrum of **1**....67
Figure S12 IR spectrum of **1** 67
Figure S13 ^{1}H NMR spectrum of **2** (600 MHz, $CDCl_3$) 68
Figure S14 ^{13}C NMR spectrum of **2** (125 MHz, $CDCl_3$) 68
Figure S15 COSY spectrum of **2**....69
Figure S16 HSQC spectrum of **2** 69
Figure S17 HMBC spectrum of **2** 70
Figure S18 NOESY spectrum of **2**....70
Figure S19 HRESIMS spectrum of **2**....71
Figure S20 UV spectrum of **2** 72
Figure S21 IR spectrum of **2**....72
Figure S22 ^{1}H NMR spectrum of **3** (600 MHz, $CDCl_3$) 73
Figure S23 ^{13}C NMR spectrum of **3** (125 MHz, $CDCl_3$)....73
Figure S24 COSY spectrum of **3** 74

Figure S25 HSQC spectrum of **3** 74
Figure S26 HMBC spectrum of **3** 75
Figure S27 NOESY spectrum of **3** 75
Figure S28 HRESIMS spectrum of **3** 76
Figure S29 UV spectrum of **3** 77
Figure S30 IR spectrum of **3** 77
Figure S31 ^{1}H NMR spectrum of **4** (400 MHz, $CDCl_3$) 78
Figure S32 ^{13}C NMR spectrum of **4** (100 MHz, $CDCl_3$) 78
Figure S33 COSY spectrum of **4** 79
Figure S34 HSQC spectrum of **4** 79
Figure S35 HMBC spectrum of **4** 80
Figure S36 NOESY spectrum of **4** 80
Figure S37 HRESIMS spectrum of **4** 81
Figure S38 UV spectrum of **4** 82
Figure S39 IR spectrum of **4** 82
Figure S40 ^{1}H NMR spectrum of **5** (600 MHz, $CDCl_3$) 83
Figure S41 ^{13}C NMR spectrum of **5** (125 MHz, $CDCl_3$) 83
Figure S42 COSY spectrum of **5** 84
Figure S43 HSQC spectrum of **5** 84
Figure S44 HMBC spectrum of **5** 85
Figure S45 NOESY spectrum of **5** 85
Figure S46 HRESIMS spectrum of **5** 86
Figure S47 UV spectrum of **5** 87
Figure S48 IR spectrum of **5** 87
Figure S49 ^{1}H NMR spectrum of **6** (600 MHz, $CDCl_3$) 88
Figure S50 ^{13}C NMR spectrum of **6** (125 MHz, $CDCl_3$) 88

Figure S51 COSY spectrum of **6** 89
Figure S52 HSQC spectrum of **6** 89
Figure S53 HMBC spectrum of **6** 90
Figure S54 NOESY spectrum of **6** 90
Figure S55 HRESIMS spectrum of **6** 91
Figure S56 UV spectrum of **6** 92
Figure S57 IR spectrum of **6** 92
Figure S58 ^{1}H NMR spectrum of **7** (400 MHz, $CDCl_3$) 93
Figure S59 ^{13}C NMR spectrum of **7** (100 MHz, $CDCl_3$) 93
Figure S60 COSY spectrum of **7** 94
Figure S61 HSQC spectrum of **7** 94
Figure S62 HMBC spectrum of **7** 95
Figure S63 NOESY spectrum of **7** 95
Figure S64 HRESIMS spectrum of **7** 96
Figure S65 UV spectrum of **7** 97
Figure S66 IR spectrum of **7** 97
Figure S67. ^{1}H NMR spectrum of **8** (600 MHz, $CDCl_3$) 98
Figure S68 ^{13}C NMR spectrum of **8** (125 MHz, $CDCl_3$) 98
Figure S69 COSY spectrum of **8** 99
Figure S70 HSQC spectrum of **8** 99
Figure S71 HMBC spectrum of **8** 100
Figure S72 NOESY spectrum of **8** 100
Figure S73 HRESIMS spectrum of **8** 101
Figure S74 UV spectrum of **8** 102
Figure S75 IR spectrum of **8** 102

Table S1 Energy analyses of 1*R*,4*S*,5*R*,8*S*,10*S*-**5** (seven conformers)

NO.	3D conformers B3LYP/6-31G(d,p)	E (Hartree)	ΔE (KJ/mol)	Boltzmann distribution
1*R*,4*S*,5*R*,8*S*,10*S*-**5** (conformer 1)	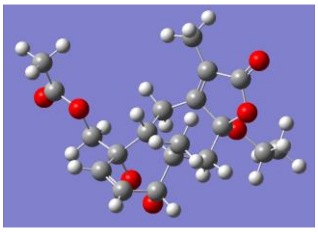	-1264.568384	2.31831548	27.52%
1*R*,4*S*,5*R*,8*S*,10*S*-**5** (conformer 2)	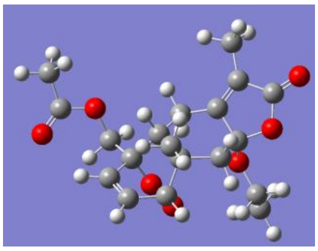	-1264.563209	15.905272	0.11%
1*R*,4*S*,5*R*,8*S*,10*S*-**5** (conformer 3)	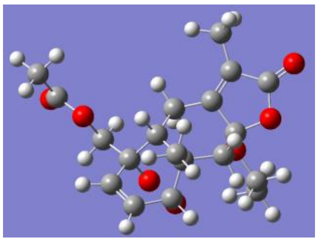	-1264.563995	13.84162991	0.26%
1*R*,4*S*,5*R*,8*S*,10*S*-**5** (conformer 4)	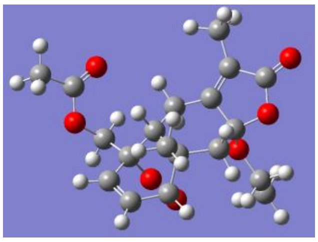	-1264.563230	15.85013653	0.12%

1*R*,4*S*,5*R*,8*S*,10*S*-**5** (conformer 5)	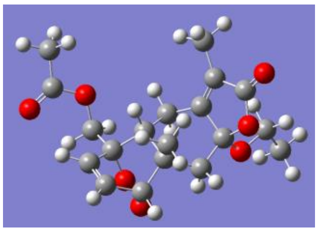	-1264.569267	0	70.12%
1*R*,4*S*,5*R*,8*S*,10*S*-**5** (conformer 6)	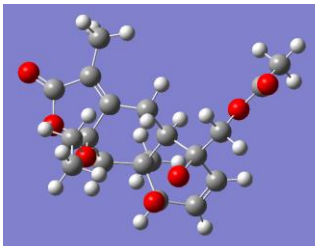	-1264.565610	9.601449275	1.46%
1*R*,4*S*,5*R*,8*S*,10*S*-**5** (conformer 7)	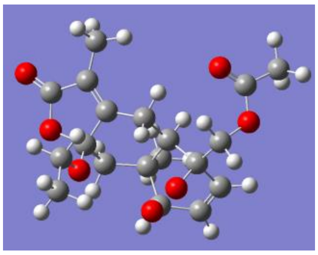	-1264.564414	12.74154589	0.41%

Table S2 Cartesian coordinates of the low-energy re-optimized conformers of 1*R*,4*S*,5*R*,8*S*,10*S*-**5** calculated at B3LYP/6-31G(d,p) level of theory.

1*R*,4*S*,5*R*,8*S*,10*S*-**5** (conformer 1)							
Atomic	Standard Orientation (Å)			Atomic	Standard Orientation (Å)		
Type	X	Y	Z	Type	X	Y	Z
C	-1.729375	-2.5384	1.124891	H	1.483165	-1.928645	-0.484878
C	-2.571663	-1.755794	1.124891	H	2.372164	-1.813036	1.035669
C	-1.929956	-1.093283	-0.072254	H	-0.393706	-0.615566	3.151261
C	-0.744147	-0.211507	0.414901	H	1.210964	-1.375316	3.123082
C	0.299229	-1.161384	1.147177	H	1.017151	0.300801	2.602778
C	-0.362124	-2.57292	1.169931	H	0.073187	3.641043	0.009474
C	-0.125424	0.658086	-0.718521	H	0.939344	3.846636	1.551095
C	1.205834	1.10365	-0.227957	H	1.703988	4.343956	0.04919
C	2.255567	0.021529	-0.12201	H	-2.471358	-0.032336	-1.900929
C	1.650264	-1.298367	0.391621	H	-3.743433	-1.076423	-1.254364
C	1.661357	2.225443	0.343722	H	5.315012	-1.572163	-3.080365
C	2.983775	1.909758	0.956496	H	3.694491	-1.142568	-3.670774
O	3.244063	0.565941	0.776075	H	4.806185	0.130813	-3.128611
C	0.544213	-0.685435	2.590782	H	4.693712	-0.760848	-0.782807
O	3.719596	2.6354	1.576649	H	3.596792	-2.050028	-1.329505
C	1.052575	3.582019	0.493103	H	-5.317629	2.157765	0.972736
C	-2.937602	-0.390301	-0.980193	H	-4.101985	3.228284	0.27355
O	-1.388033	-2.123791	-0.945408	H	-5.715316	3.109203	-0.488944
O	-0.521151	-3.048866	-0.189458				
O	2.822346	-0.111667	-1.405195				
C	4.467857	-0.892328	-2.937288				
C	3.927588	-1.019612	-1.523121				
O	-3.467108	0.718365	-0.230669				
O	-4.85172	1.16875	-1.960908				
C	-4.447491	1.430508	-0.852487				
C	-4.931236	2.556387	0.029004				
H	-1.989983	-3.141636	2.66472				
H	-3.622836	-1.610371	1.354641				
H	-1.145467	0.482678	1.159609				
H	0.30744	-3.320297	1.606886				
H	-0.002582	0.058611	-1.628594				
H	-0.788697	1.498064	-0.944715				

1*R*,4*S*,5*R*,8*S*,10*S*-**5** (conformer 2)							
Atomic	Standard Orientation (Å)			Atomic	Standard Orientation (Å)		
Type	X	Y	Z	Type	X	Y	Z
C	-1.940982	-2.250766	1.51976	H	2.02092	-1.675424	1.53728
C	-2.655418	-1.459736	0.719216	H	1.998282	-0.063443	2.205588
C	-1.798167	-0.716079	-0.280429	H	-1.376412	-0.270153	3.087152
C	-0.750076	0.202735	0.42927	H	0.189795	-0.884569	3.649735
C	0.053576	-0.697111	1.467914	H	0.050592	0.78084	3.068209
C	-0.465577	-2.14807	1.247506	H	0.990942	3.916651	-0.620202
C	0.07681	0.977188	-0.644212	H	2.30814	4.308641	0.51173
C	1.487856	1.198226	-0.245466	H	2.662726	4.089729	-1.195291
C	2.249411	-0.048821	0.119599	H	-1.992257	0.27421	-2.207714
C	1.621101	-0.668764	1.376586	H	-3.413559	-0.687391	-1.72526
C	2.287508	2.266286	-0.135019	H	3.652212	-3.605638	-2.326908
C	3.629406	1.782736	0.295722	H	2.133173	-2.796542	-2.772668
O	3.578436	0.414938	0.454991	H	3.682866	-1.949488	-2.974806
C	-0.299059	-0.244926	2.903435	H	3.99003	-1.924199	-0.480886
O	4.627574	2.425207	0.509554	H	2.446955	-2.803701	-0.296938
C	2.038339	3.720432	-0.372998	H	-6.023641	2.531563	0.17994
C	-2.626328	-0.020398	-1.371215	H	-4.365125	2.904503	0.740303
O	-1.052906	-1.711569	-1.036487	H	-4.827544	3.278276	-0.920587
O	-0.22374	-2.531025	-0.12975				
O	2.290887	-0.856617	-1.016315				
C	3.126214	-2.644554	-2.338318				
C	3.003139	-2.098472	-0.92628				
O	-3.205769	1.196612	-0.862338				
O	-5.235862	0.207311	-0.606229				
C	-4.529224	1.187311	-0.540902				
C	-4.971653	2.562551	-0.104539				
H	-2.352244	-2.932457	2.257818				
H	-3.738247	-1.395356	0.688051				
H	-1.307293	0.945954	1.006954				
H	0.132148	-2.874778	1.805973				
H	0.11963	0.361761	-1.551455				
H	-0.432304	1.91356	-0.891213				

1*R*,4*S*,5*R*,8*S*,10*S*-**5** (conformer 3)							
Atomic	Standard Orientation (Å)			Atomic	Standard Orientation (Å)		
Type	X	Y	Z	Type	X	Y	Z
C	-1.602693	-2.689494	1.493784	H	2.279612	-1.750881	1.230385
C	-2.454084	-1.870525	0.877386	H	2.195207	-0.245598	2.109507
C	-1.768767	-0.930365	-0.086827	H	-1.042589	-0.880062	3.261268
C	-0.734717	-0.014609	0.646863	H	0.617594	-1.414485	3.588018
C	0.242696	-0.960875	1.474384	H	0.286296	0.291599	3.255611
C	-0.175922	-2.414054	1.103653	H	0.589916	3.951906	-0.038942
C	-0.088662	0.968489	-0.378952	H	1.984893	4.314087	1.006439
C	1.335234	1.263973	-0.087978	H	2.172639	4.34858	-0.740387
C	2.226678	0.055812	0.030069	H	-2.276242	0.389769	-1.759513
C	1.784806	-0.774142	1.244541	H	-3.43849	-0.907325	-1.463133
C	2.055445	2.378113	0.091827	H	3.632285	-3.01724	-3.001442
C	3.469083	1.968826	0.324895	H	2.008623	-2.312876	-3.172152
O	3.54283	0.59298	0.29935	H	3.447076	-1.299937	-3.425683
C	0.00466	-0.732439	2.984511	H	4.044094	-1.547084	-0.996648
O	4.430387	2.667476	0.530751	H	2.612703	-2.589068	-0.766772
C	1.669618	3.821774	0.079231	H	-6.000044	2.840727	-0.230176
C	-2.768262	-0.191951	-0.978306	H	-5.667468	1.653482	1.065686
O	-1.028202	-1.745485	-1.036024	H	-4.516957	2.956482	0.762861
O	-0.044994	-2.589983	-0.328716				
O	2.210939	-0.593506	-1.203673				
C	3.029904	-2.118165	-2.830267				
C	3.029683	-1.761737	-1.353774				
O	-3.519953	0.67951	-0.113865				
O	-4.735903	1.332635	-1.90491				
C	-4.501008	1.40222	-0.721409				
C	-5.222	2.27211	0.279721				
H	-1.878629	-3.489231	2.173994				
H	-3.535354	-1.890229	0.973743				
H	-1.292624	0.587989	1.369272				
H	0.532606	-3.147202	1.500706				
H	-0.096782	0.488564	-1.365571				
H	-0.689344	1.881241	-0.440496				

•

1*R*,4*S*,5*R*,8*S*,10*S*-**5** (conformer 4)							
Atomic	Standard Orientation (Å)			Atomic	Standard Orientation (Å)		
Type	X	Y	Z	Type	X	Y	Z
C	-1.783759	-2.6785	1.332878	H	2.07927	-1.645468	1.52729
C	-2.590844	-1.898829	0.61414	H	1.848206	-0.064877	2.229555
C	-1.837493	-0.982855	-0.324262	H	-1.475557	-0.777557	3.044133
C	-0.87051	-0.018225	0.441144	H	0.142484	-1.262188	3.585558
C	0.013504	-0.906765	1.424088	H	-0.17415	0.420619	3.140302
C	-0.333111	-2.382914	1.069209	H	0.496836	3.878863	-0.434452
C	-0.115071	0.881137	-0.588697	H	1.767111	4.359683	0.71774
C	1.267702	1.214651	-0.170199	H	2.142492	4.244123	-0.995016
C	2.148165	0.037838	0.155005	H	-2.268685	0.429337	-1.953502
C	1.567188	-0.688909	1.37645	H	-3.202566	-1.090256	-1.980231
C	1.954698	2.352616	-0.007032	H	3.926775	-3.267415	-2.39818
C	3.333445	1.989914	0.424189	H	2.323592	-2.618762	-2.813061
O	3.41858	0.617595	0.531827	H	3.76923	-1.602487	-3.003863
C	-0.405944	-0.619617	2.884407	H	4.094862	-1.60704	-0.510629
O	4.260541	2.718396	0.678737	H	2.659064	-2.65268	-0.340986
C	1.558444	3.781897	-0.190125	H	-5.187654	3.173104	0.246354
C	-2.776785	-0.313254	-1.340875	H	-6.068302	1.779867	-0.446349
O	-1.010996	-1.835019	-1.166245	H	-5.268862	1.610202	1.116967
O	-0.061306	-2.604459	-0.337937				
O	2.287582	-0.715922	-1.01032				
C	3.298218	-2.370056	-2.381729				
C	3.128925	-1.874682	-0.955822				
O	-3.889804	0.292604	-0.655752				
O	-3.074338	2.386765	-0.998089				
C	-3.930663	1.64901	-0.564991				
C	-5.193324	2.088559	0.13534				
H	-2.106843	-3.460459	2.013114				
H	-3.675093	-1.939614	0.612231				
H	-1.483319	0.64183	1.06431				
H	0.349877	-3.084067	1.558135				
H	-0.018425	0.314881	-1.523932				
H	-0.709144	1.774596	-0.79195				

1*R*,4*S*,5*R*,8*S*,10*S*-**5** (conformer 5)							
Atomic	Standard Orientation (Å)			Atomic	Standard Orientation (Å)		
Type	X	Y	Z	Type	X	Y	Z
C	-2.252963	-2.189028	1.577042	H	1.306929	-2.228109	-0.246135
C	-2.878686	-1.339229	0.760699	H	1.99205	-2.078771	1.370203
C	-1.997245	-0.855261	-0.369223	H	-0.838715	-0.382068	2.996276
C	-0.755751	-0.127839	0.223068	H	0.619359	-1.366123	3.235983
C	0.026168	-1.163437	1.141149	H	0.756706	0.279448	2.610892
C	-0.838383	-2.460191	1.139447	H	0.635308	3.508674	-0.210176
C	0.129451	0.54775	-0.865581	H	1.290222	3.654553	1.438315
C	1.439333	0.834677	-0.220755	H	2.32114	4.002814	0.0599
C	2.289502	-0.382156	0.076117	H	-2.13457	0.032012	-2.342514
C	1.42905	-1.545142	0.596605	H	-3.68296	-0.582378	-1.703986
C	1.960638	1.915393	0.376147	H	5.239754	-0.258158	-3.399287
C	3.134853	1.456819	1.173246	H	4.963616	-1.772653	-2.510935
O	3.255292	0.08989	1.039344	H	3.698735	-1.127776	-3.575043
C	0.143878	-0.626527	2.57979	H	3.44308	0.864714	-2.059111
O	3.86509	2.107591	1.878858	H	4.710636	0.21577	-0.996723
C	1.519474	3.343194	0.41223	H	-5.432031	3.20591	0.144309
C	-2.753404	-0.071442	-1.449773	H	-3.687311	3.276592	0.537089
O	-1.515604	-2.010936	-1.112674	H	-4.224957	3.625477	-1.106874
O	-0.895432	-2.996906	-0.206035				
O	2.956511	-0.898333	-1.053815				
C	4.488809	-0.853617	-2.86849				
C	3.91983	-0.059712	-1.704201				
O	-3.035508	1.269345	-1.003301				
O	-5.186072	0.725382	-0.509563				
C	-4.300286	1.546396	-0.580032				
C	-4.42567	3.007848	-0.225234				
H	-2.711884	-2.699175	2.418265				
H	-3.922117	-1.047883	0.823833				
H	-1.128668	0.675894	0.865905				
H	-0.349162	-3.266069	1.695281				
H	0.273941	-0.142238	-1.705837				
H	-0.364901	1.450318	-1.235667				

1*R*,4*S*,5*R*,8*S*,10*S*-**5** (conformer 6)							
Atomic	Standard Orientation (Å)			Atomic	Standard Orientation (Å)		
Type	X	Y	Z	Type	X	Y	Z
C	1.820945	-2.806476	-1.252528	H	-2.118416	-2.16261	-1.013716
C	2.599499	-1.867662	-0.715499	H	-2.153578	-0.821405	-2.148821
C	1.834352	-0.907211	0.165038	H	1.165386	-1.184195	-3.159469
C	0.725268	-0.149165	-0.636267	H	-0.42795	-1.900857	-3.467727
C	-0.154111	-1.236615	-1.394913	H	-0.267691	-0.149106	-3.278368
C	0.373509	-2.613139	-0.888888	H	-0.785873	3.730066	-0.318684
C	-0.021692	0.828546	0.321472	H	-2.072693	3.90665	-1.535781
C	-1.441967	1.027351	-0.058464	H	-2.44643	4.137183	0.164837
C	-2.274137	-0.230656	-0.131517	H	2.218252	0.593246	1.713645
C	-1.713294	-1.164343	-1.208648	H	3.487625	-0.621356	1.530891
C	-2.180279	2.089468	-0.407331	H	-3.238746	-0.798461	4.270193
C	-3.555157	1.598181	-0.708541	H	-3.499141	-2.129751	3.12123
O	-3.588177	0.23407	-0.530694	H	-1.847574	-1.636613	3.540523
C	0.102381	-1.114795	-2.914738	H	-2.242044	0.596817	2.447469
O	-4.516464	2.232868	-1.067773	H	-3.884164	0.08546	2.002899
C	-1.842469	3.540122	-0.528663	H	5.688775	3.248735	-0.020856
C	2.762343	-0.010752	0.985562	H	5.541773	1.892537	-1.176686
O	1.163482	-1.69845	1.182413	H	4.244425	3.082925	-1.063952
O	0.243675	-2.666335	0.552507				
O	-2.355664	-0.955334	1.056936				
C	-2.862553	-1.270997	3.356139				
C	-2.86927	-0.274412	2.209872				
O	3.443977	0.842398	0.04793				
O	4.547243	1.78865	1.779995				
C	4.336513	1.714522	0.592079				
C	4.998473	2.542427	-0.482693				
H	2.165651	-3.63902	-1.857946				
H	3.679347	-1.807331	-0.811034				
H	1.2269	0.457099	-1.396104				
H	-0.271968	-3.433221	-1.216401				
H	-0.01586	0.385103	1.325331				
H	0.519295	1.7788	0.369176				

1*R*,4*S*,5*R*,8*S*,10*S*-**5** (conformer 7)							
Atomic	Standard Orientation (Å)			Atomic	Standard Orientation (Å)		
Type	X	Y	Z	Type	X	Y	Z
C	2.042624	-2.661251	-1.258445	H	-1.885008	-1.949299	-1.593119
C	2.766755	-1.793745	-0.551542	H	-1.731852	-0.387314	-2.382081
C	1.920116	-0.928884	0.355537	H	1.675755	-0.809383	-2.987074
C	0.872722	-0.073014	-0.435099	H	0.140291	-1.451254	-3.598696
C	0.116794	-1.044735	-1.442511	H	0.277391	0.261331	-3.177511
C	0.566407	-2.480447	-1.031605	H	-0.738403	3.669221	0.372887
C	-0.007414	0.718103	0.582666	H	-1.915233	4.080194	-0.898961
C	-1.380185	0.970583	0.080113	H	-2.441719	3.980141	0.773635
C	-2.16838	-0.249544	-0.331417	H	2.187412	0.527804	1.979091
C	-1.450188	-0.949387	-1.487349	H	3.288592	-0.874626	2.01116
C	-2.093594	2.080388	-0.155756	H	-3.651222	-1.650543	3.724642
C	-3.408802	1.656901	-0.713635	H	-3.767878	-2.725351	2.31347
O	-3.432325	0.283039	-0.801787	H	-2.17958	-2.356431	3.012317
C	0.589755	-0.747944	-2.885563	H	-2.451337	0.055821	2.343539
O	-4.333165	2.347451	-1.067576	H	-4.024454	-0.328458	1.613006
C	-1.768972	3.52661	0.035741	H	4.766527	3.57918	-0.239405
C	2.777143	-0.153952	1.368595	H	5.803605	2.30232	0.461233
O	1.168397	-1.841245	1.202622	H	5.03	2.031265	-1.100671
O	0.281913	-2.683442	0.375896				
O	-2.385583	-1.191278	0.672678				
C	-3.166959	-1.942006	2.786051				
C	-3.039943	-0.740288	1.865666				
O	3.814072	0.57259	0.681507				
O	2.758127	2.558786	1.008751				
C	3.695879	1.923255	0.581109				
C	4.898767	2.503404	-0.121911				
H	2.444632	-3.430453	-1.910615				
H	3.850445	-1.740387	-0.530892				
H	1.42051	0.661449	-1.035309				
H	-0.048129	-3.247839	-1.51087				
H	-0.098637	0.104862	1.488384				
H	0.501658	1.646234	0.851248				

Table S3 Energy analyses of 1*S*,4*R*,5*S*,8*S*,10*R*-**5** (eight conformers)

**NO.**	**3D conformers** B3LYP/6-31G(d,p)	**E (Hartree)**	**ΔE (KJ/mol)**	**Boltzmann distribution**
1*S*,4*R*,5*S*,8*S*,10*R*-**5** (conformer 1)	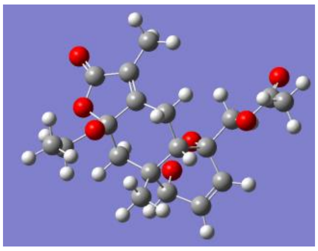	-1264.567929	5.84173493	5.90%
1*S*,4*R*,5*S*,8*S*,10*R*-**5** (conformer 2)	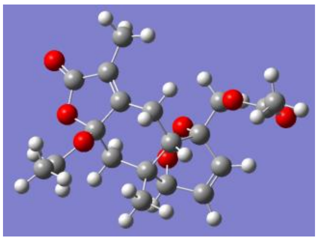	-1264.567006	8.265070363	2.22%
1*S*,4*R*,5*S*,8*S*,10*R*-**5** (conformer 3)	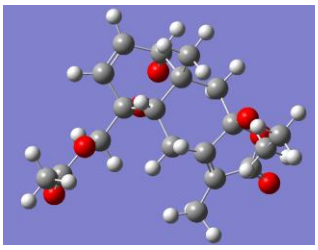	-1264.570154	0	62.27%
1*S*,4*R*,5*S*,8*S*,10*R*-**5** (conformer 4)	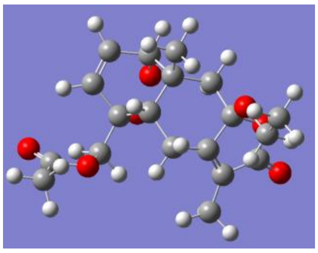	-1264.569214	2.467968914	23.01%

1*S*,4*R*,5*S*,8*S*,10*R*-**5** (conformer 5)	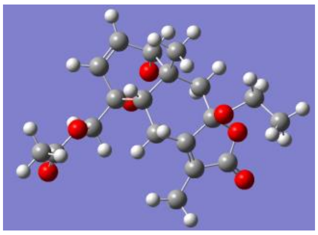	-1264.566969	8.362213821	2.13%
1*S*,4*R*,5*S*,8*S*,10*R*-**5** (conformer 6)	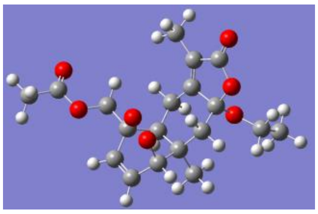	-1264.567201	7.753098088	2.73%
1*S*,4*R*,5*S*,8*S*,10*R*-**5** (conformer 7)	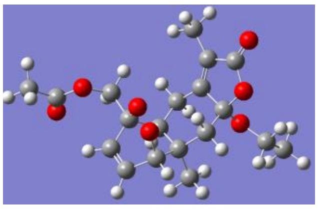	-1264.566017	10.86168872	0.78%
1*S*,4*R*,5*S*,8*S*,10*R*-**5** (conformer 8)	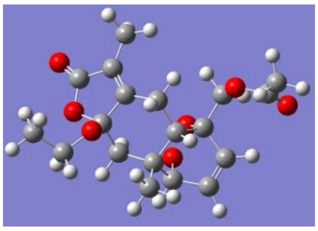	-1264.566217	10.33658895	0.96%

Table S4 Cartesian coordinates of the low-energy re-optimized conformers of 1*S*,4*R*,5*S*,8*S*,10*R*-**5** calculated at B3LYP/6-31G(d,p) level of theory.

1*S*,4*R*,5*S*,8*S*,10*R*-**5** (conformer 1)							
Atomic	Standard Orientation (Å)			Atomic	Standard Orientation (Å)		
Type	X	Y	Z	Type	X	Y	Z
C	1.567642	-3.102684	-0.918507	H	-2.537107	-1.833471	-0.740383
C	2.428696	-2.123244	-0.639176	H	-1.522495	-0.793163	-1.726752
C	1.791175	-0.750418	-0.660481	H	-1.268345	-2.189642	1.873327
C	0.66088	-0.762835	0.418622	H	-1.581119	-3.469604	0.689665
C	-0.452933	-1.767767	-0.099698	H	0.037294	-3.290672	1.393675
C	0.205922	-2.56377	-1.27386	H	0.446375	3.45792	-0.240316
C	0.141716	0.625109	0.901536	H	-0.401333	3.703877	-1.784277
C	-1.035	1.12373	0.145043	H	-1.072729	4.376503	-0.305061
C	-2.194006	0.165047	0.032397	H	3.592767	0.240147	-1.326629
C	-1.72771	-1.094844	-0.698136	H	2.354145	1.355336	-0.742605
C	-1.3257	2.258902	-0.502152	H	-5.354778	-1.183299	3.007925
C	-2.692529	2.115302	-1.073269	H	-3.664946	-1.112314	3.551582
O	-3.169111	0.854625	-0.766577	H	-4.53624	0.386387	3.171539
C	-0.836897	-2.738872	1.030934	H	-4.675986	-0.29376	0.75878
O	-3.330832	2.906957	-1.719501	H	-3.818382	-1.80408	1.136501
C	-0.538271	3.511007	-0.713927	H	5.66958	1.825857	2.58383
C	2.814155	0.379054	-0.571907	H	4.08661	1.201786	3.134585
O	1.189397	-0.512124	-1.965714	H	5.305265	0.075053	2.53571
O	0.400609	-1.676903	-2.402101				
O	-2.696075	-0.052388	1.331323				
C	-4.40124	-0.659493	2.878889				
C	-3.945103	-0.753128	1.432573				
O	3.389855	0.321784	0.7458				
O	4.711702	2.072199	0.196219				
C	4.35684	1.245613	1.003413				
C	4.893738	1.081143	2.404533				
H	1.815608	-4.157564	-0.982944				
H	3.484959	-2.250756	-0.42209				
H	1.120591	-1.217483	1.300757				
H	-0.479613	-3.317987	-1.672541				
H	-0.185897	0.494287	1.941791				
H	0.95318	1.35634	0.927379				

1*S*,4*R*,5*S*,8*S*,10*R*-**5** (conformer 2)							
Atomic	Standard Orientation (Å)			Atomic	Standard Orientation (Å)		
Type	X	Y	Z	Type	X	Y	Z
C	1.98683	-2.515903	-1.264098	H	-2.264523	-1.961677	-0.962804
C	2.686202	-1.452686	-0.863447	H	-1.444631	-0.659118	-1.808806
C	1.834204	-0.208607	-0.718358	H	-0.921243	-2.4151	1.600949
C	0.730699	-0.529571	0.340597	H	-1.040326	-3.577315	0.269664
C	-0.211718	-1.63368	-0.302468	H	0.537882	-3.225352	0.998554
C	0.551853	-2.166087	-1.558436	H	-0.183151	3.647625	0.195572
C	-0.002609	0.689289	0.980375	H	-1.068905	3.93244	-1.321314
C	-1.254458	1.07397	0.280337	H	-1.832054	4.307781	0.217479
C	-2.243211	-0.040677	0.046381	H	3.496417	1.094388	-1.183511
C	-1.586243	-1.111048	-0.82549	H	2.028216	1.948308	-0.642412
C	-1.735549	2.21498	-0.228379	H	-5.102368	-2.232079	2.818627
C	-3.067431	1.91919	-0.822094	H	-3.439812	-1.949801	3.377641
O	-3.327915	0.570476	-0.671945	H	-4.549693	-0.580035	3.173733
C	-0.418792	-2.782262	0.700821	H	-4.608147	-0.982409	0.696079
O	-3.834197	2.667668	-1.373538	H	-3.509773	-2.365392	0.896501
C	-1.165723	3.595243	-0.28267	H	5.803149	0.74392	2.739684
C	2.653939	1.066625	-0.491485	H	4.642436	2.101642	2.817789
O	1.184244	0.085059	-1.990684	H	4.092371	0.46756	3.190803
O	0.586699	-1.1296	-2.570697				
O	-2.687186	-0.488995	1.306565				
C	-4.249248	-1.547318	2.759173				
C	-3.803061	-1.392295	1.315021				
O	3.131106	1.139014	0.866189				
O	5.206747	0.448892	0.249473				
C	4.437143	0.832072	1.100755				
C	4.771709	1.046009	2.556682				
H	2.402724	-3.498041	-1.466631				
H	3.756166	-1.424518	-0.684315				
H	1.264596	-1.006013	1.168216				
H	-0.008979	-2.966946	-2.050314				
H	-0.293251	0.383152	1.994578				
H	0.679695	1.533404	1.102035				

1*S*,4*R*,5*S*,8*S*,10*R*-**5** (conformer 4)							
Atomic	Standard Orientation (Å)			Atomic	Standard Orientation (Å)		
Type	X	Y	Z	Type	X	Y	Z
C	-2.194849	2.68452	-0.592489	H	2.114612	2.438812	-0.379856
C	-2.791239	1.496813	-0.476131	H	1.381202	1.37613	-1.58611
C	-1.832748	0.337314	-0.653321	H	0.796802	2.141593	2.146675
C	-0.737132	0.474163	0.452283	H	0.73398	3.600929	1.156033
C	0.090205	1.787383	0.118495	H	-0.770297	2.905177	1.792018
C	-0.741694	2.549446	-0.96373	H	0.493025	-3.411153	-0.824291
C	0.114648	-0.798737	0.75051	H	1.280507	-3.141085	-2.39654
C	1.372848	-0.878749	-0.036976	H	2.180527	-3.900643	-1.091913
C	2.260469	0.342785	0.029164	H	-3.390312	-0.943843	-1.434277
C	1.495723	1.544022	-0.513912	H	-1.842859	-1.775867	-1.13516
C	1.909608	-1.787759	-0.86128	H	4.55736	-0.451438	3.873674
C	3.191148	-1.225718	-1.373179	H	4.50977	1.182992	3.178006
O	3.37256	0.030425	-0.832579	H	3.059863	0.502348	3.942841
C	0.215025	2.660658	1.379978	H	2.932633	-1.245512	2.133413
O	3.978725	-1.722612	-2.139163	H	4.388657	-0.565655	1.377779
C	1.431937	-3.130389	-1.31069	H	-5.624867	-1.828186	2.444315
C	-2.536209	-1.020456	-0.760483	H	-4.329396	-3.03382	2.186851
O	-1.187925	0.435098	-1.957673	H	-3.942344	-1.49418	2.956461
O	-0.708098	1.804891	-2.206922				
O	2.718237	0.664404	1.31825				
C	3.933749	0.264368	3.327037				
C	3.513381	-0.322731	1.990492				
O	-2.971975	-1.480038	0.534008				
O	-5.115063	-0.849808	0.115347				
C	-4.294011	-1.363146	0.840507				
C	-4.572369	-1.966122	2.195695				
H	-2.697811	3.645022	-0.537652				
H	-3.850603	1.32915	-0.310716				
H	-1.290097	0.668133	1.376153				
H	-0.261711	3.494893	-1.234501				
H	0.400237	-0.736451	1.810051				
H	-0.491524	-1.702223	0.654798				

1*S*,4*R*,5*S*,8*S*,10*R*-**5** (conformer 5)							
Atomic	Standard Orientation (Å)			Atomic	Standard Orientation (Å)		
Type	X	Y	Z	Type	X	Y	Z
C	-1.725006	3.133729	-0.660228	H	2.42928	2.038375	-0.483278
C	-2.545173	2.097504	-0.480554	H	1.485494	1.03908	-1.580388
C	-1.844477	0.760638	-0.596435	H	1.09644	2.124218	2.114151
C	-0.737688	0.736139	0.506522	H	1.375721	3.506221	1.043821
C	0.338613	1.828031	0.095749	H	-0.247616	3.204043	1.693483
C	-0.333153	2.686616	-1.025737	H	-0.307972	-3.404607	-0.479352
C	-0.164526	-0.661929	0.889518	H	0.587316	-3.490987	-2.013488
C	1.05143	-1.043811	0.127161	H	1.254084	-4.245419	-0.572032
C	2.164059	-0.025939	0.118618	H	-3.584108	-0.252149	-1.382889
C	1.654224	1.263121	-0.525549	H	-2.307492	-1.35459	-0.859713
C	1.410836	-2.109487	-0.599212	H	5.882849	0.349331	2.298499
C	2.782553	-1.860195	-1.119252	H	4.626853	-0.704202	2.98383
O	3.191272	-0.606128	-0.703425	H	5.142317	-0.920088	1.296345
C	0.657033	2.720351	1.308783	H	4.185121	1.304056	0.700043
O	3.475166	-2.570239	-1.803372	H	3.710931	1.57558	2.386335
C	0.689216	-3.37593	-0.928155	H	-5.666821	-2.238426	2.336247
C	-2.815643	-0.416701	-0.622948	H	-4.127076	-1.589429	2.973982
O	-1.206051	0.655618	-1.90188	H	-5.384374	-0.474751	2.435449
O	-0.464792	1.886388	-2.225768				
O	2.62072	0.127498	1.441633				
C	4.948081	-0.180032	2.077617				
C	3.879283	0.804343	1.624945				
O	-3.420495	-0.491742	0.68072				
O	-4.648887	-2.248444	-0.03919				
C	-4.349457	-1.474881	0.839847				
C	-4.923186	-1.447484	2.235728				
H	-2.020244	4.178256	-0.645774				
H	-3.6103	2.159905	-0.278858				
H	-1.234955	1.097251	1.411225				
H	0.324464	3.500454	-1.3466				
H	0.133474	-0.601421	1.945084				
H	-0.940962	-1.428817	0.835627				

1*S*,4*R*,5*S*,8*S*,10*R*-**5** (conformer 6)							
Atomic	Standard Orientation (Å)			Atomic	Standard Orientation (Å)		
Type	X	Y	Z	Type	X	Y	Z
C	-1.522574	-3.026358	0.644759	H	2.457982	-1.561025	1.339854
C	-2.292544	-2.163065	-0.019584	H	1.200799	-0.428478	1.810671
C	-1.699975	-0.771124	-0.080374	H	1.953916	-2.435178	-1.41315
C	-0.316403	-0.902317	-0.800527	H	1.951931	-3.470256	0.023728
C	0.628753	-1.73333	0.165384	H	0.58221	-3.516934	-1.102001
C	-0.314746	-2.355537	1.24548	H	-0.336165	3.373812	-0.895046
C	0.302931	0.408629	-1.378374	H	0.066705	3.939754	0.742311
C	1.223418	1.101517	-0.44077	H	1.101591	4.376958	-0.61006
C	2.318187	0.248654	0.149854	H	-2.278716	1.278474	-0.479958
C	1.683582	-0.890824	0.947984	H	-2.659589	0.144114	-1.78773
C	1.31385	2.347559	0.039695	H	6.180477	-1.411047	-1.58024
C	2.474705	2.387949	0.97071	H	4.698127	-1.54502	-2.550201
O	3.030558	1.124335	1.038699	H	5.418446	0.048893	-2.249907
C	1.317891	-2.857099	-0.629034	H	4.913057	-0.172609	0.199886
O	2.903242	3.316801	1.607195	H	4.204026	-1.775799	-0.093261
C	0.485782	3.567984	-0.199859	H	-6.941707	1.426332	-0.302978
C	-2.6254	0.265748	-0.699157	H	-6.521692	-0.300119	-0.080526
O	-1.474224	-0.27906	1.273051	H	-6.103984	0.86695	1.174515
O	-0.81733	-1.303398	2.103314				
O	3.157295	-0.160299	-0.906029				
C	5.221519	-0.932765	-1.807991				
C	4.39488	-0.790338	-0.541145				
O	-3.941918	0.087347	-0.155641				
O	-4.628562	1.864546	-1.371535				
C	-4.86997	0.985995	-0.574511				
C	-6.199792	0.73178	0.091993				
H	-1.76737	-4.067323	0.831707				
H	-3.260002	-2.385371	-0.456713				
H	-0.514359	-1.532819	-1.674059				
H	0.245718	-2.990266	1.938815				
H	0.903911	0.115373	-2.249674				
H	-0.472369	1.081073	-1.755601				

1*S*,4*R*,5*S*,8*S*,10*R*-**5** (conformer 7)							
Atomic	Standard Orientation (Å)			Atomic	Standard Orientation (Å)		
Type	X	Y	Z	Type	X	Y	Z
C	-1.910473	-2.517829	0.91124	H	2.254033	-1.615454	1.466032
C	-2.552472	-1.602045	0.183944	H	1.188814	-0.269849	1.838322
C	-1.757313	-0.324281	0.012012	H	1.576046	-2.632954	-1.205028
C	-0.428045	-0.730694	-0.7105	H	1.447867	-3.53249	0.314896
C	0.400353	-1.611557	0.317081	H	0.064977	-3.466882	-0.793719
C	-0.605515	-1.995134	1.452662	H	0.216137	3.47083	-1.218027
C	0.376708	0.408044	-1.411114	H	0.688676	4.10173	0.376902
C	1.405131	1.036385	-0.542976	H	1.784427	4.272061	-0.987625
C	2.368127	0.08341	0.120523	H	-1.977991	1.740731	-0.576131
C	1.582503	-0.873011	1.018464	H	-2.682695	0.560032	-1.726963
C	1.690838	2.291938	-0.177769	H	5.912054	-2.282689	-1.462169
C	2.858145	2.238951	0.744314	H	4.416529	-2.265513	-2.420893
O	3.21721	0.917436	0.926478	H	5.374461	-0.781481	-2.248287
C	0.899572	-2.887734	-0.383528	H	4.866283	-0.726612	0.209995
O	3.431566	3.14617	1.292022	H	3.919419	-2.221935	0.045859
C	1.05374	3.597944	-0.525726	H	-7.030021	0.601109	-0.420868
C	-2.526828	0.802225	-0.672566	H	-6.09578	0.586485	1.107609
O	-1.426819	0.230191	1.317772	H	-6.206879	2.075891	0.171034
O	-0.923878	-0.815208	2.226165				
O	3.120952	-0.536742	-0.896443				
C	5.034867	-1.683218	-1.729367				
C	4.252777	-1.315995	-0.479573				
O	-3.796538	1.038737	-0.04172				
O	-4.891354	-0.031828	-1.717821				
C	-4.909468	0.593526	-0.679627				
C	-6.146667	0.985711	0.089575				
H	-2.29869	-3.499644	1.163883				
H	-3.527575	-1.728765	-0.273614				
H	-0.742661	-1.395446	-1.521869				
H	-0.134379	-2.653153	2.189471				
H	0.913997	-0.056971	-2.248607				
H	-0.292023	1.151106	-1.854223				

1*S*,4*R*,5*S*,8*S*,10*R*-**5** (conformer 8)							
Atomic	Standard Orientation (Å)			Atomic	Standard Orientation (Å)		
Type	X	Y	Z	Type	X	Y	Z
C	2.087593	-2.65243	-0.882147	H	-2.175008	-2.178979	-0.591628
C	2.758994	-1.52318	-0.649591	H	-1.405581	-0.991482	-1.635747
C	1.871226	-0.295928	-0.670141	H	-0.792268	-2.221954	1.983079
C	0.791112	-0.493535	0.441852	H	-0.895265	-3.564575	0.833897
C	-0.125517	-1.703313	-0.022764	H	0.682088	-3.073175	1.480515
C	0.639355	-2.389412	-1.200982	H	-0.255152	3.595417	-0.269125
C	0.029627	0.782407	0.915758	H	-1.167002	3.638628	-1.796572
C	-1.242938	1.028428	0.191112	H	-1.923645	4.20318	-0.313312
C	-2.198064	-0.137021	0.131261	H	3.487484	0.973536	-1.343649
C	-1.520516	-1.298569	-0.594665	H	1.999723	1.853867	-0.908656
C	-1.766471	2.072317	-0.463686	H	-5.782045	-1.178787	2.323003
C	-3.097027	1.658786	-0.985185	H	-4.678009	0.001789	3.060913
O	-3.31311	0.336487	-0.644382	H	-5.252781	0.244395	1.39637
C	-0.289686	-2.701236	1.13755	H	-3.996645	-1.775491	0.64838
O	-3.896743	2.301395	-1.617557	H	-3.454215	-2.079137	2.30842
C	-1.241096	3.447597	-0.719593	H	5.847636	1.262849	2.552718
C	2.654021	1.021733	-0.641903	H	4.640996	2.578867	2.45592
O	1.196904	-0.206474	-1.960607	H	4.152723	0.997165	3.065596
O	0.631773	-1.508791	-2.352066				
O	-2.604535	-0.431446	1.44667				
C	-4.938142	-0.506467	2.126478				
C	-3.747611	-1.294926	1.600041				
O	3.143791	1.303393	0.683595				
O	5.233	0.592447	0.140834				
C	4.461143	1.071434	0.939956				
C	4.80435	1.50172	2.345123				
H	2.530752	-3.641275	-0.948195				
H	3.82964	-1.439105	-0.49346				
H	1.349303	-0.832873	1.319456				
H	0.097383	-3.268294	-1.563837				
H	-0.238366	0.61474	1.9679				
H	0.687497	1.654158	0.905278				

Table S5 Experimental and calculated ^{1}H NMR data for compound **5**.

No.	**5**, exptl. δ_H [a]	1*R*,4*S*,5*R*,8*S*,10*S*-**5**, calcd. δ_H [b]	1*S*,4*R*,5*S*,8*S*,10*R*-**5**, calcd. δ_H [b]
1	4.2	3.96	4.02
2	6.74	7.01	6.99
3	6.39	7.07	6.85
5	2.53	1.60	1.94
6	2.47	2.73	2.62
	2.61	2.86	2.82
9	1.4	2.16	2.23
	2.26	3.29	2.60
13	1.79	1.95	1.78
14	1.61	0.67	1.19
15	4.37	3.66	3.92
	4.43	4.88	4.50
4-OAc	2.17	2.22	2.18
1′	3.15	3.37	3.03
	3.46	3.62	3.59
2′	1.21	1.28	1.23

[a] Recorded in $CDCl_3$ at 600 MHz.

[b] Calculated in $CDCl_3$

Table S6 Experimental and calculated ^{13}C NMR data for compound **5**.

No.	**5**, exptl. δ_C [a]	1*R*,4*S*,5*R*,8*S*,10*S*-**5**, calcd. δ_C [b]	1*S*,4*R*,5*S*,8*S*,10*R*-**5**, calcd. δ_C [b]
1	79.5	76.06	74.25
2	135.8	135.54	134.13
3	129.2	137.27	135.89
4	79.8	75.79	75.45
5	42.5	44.36	43.80
6	22.8	18.67	17.55
7	155.8	163.69	156.63
8	105.3	103.90	100.25
9	43.8	39.23	38.05
10	38.7	33.46	35.02
11	124.2	126.51	121.86
12	171.1	172.11	168.83
13	8.3	5.42	4.83
14	29.2	24.27	23.57
15	62.8	58.50	58.99
4-OAc	20.8	17.29	17.09
	170.7	172.53	170.57
1′	59.1	54.70	52.74
2′	15.1	10.92	10.81

[a] Recorded in $CDCl_3$ at 600 MHz.

[b] Calculated in $CDCl_3$

Table S7 DP4+ analyses of calculated and experimental NMR chemical shifts of **5** (unscaled). Isomer 1: 1*R*,4*S*,5*R*,8*S*,10*S*-**5**; Isomer 2: 1*S*,4*R*,5*S*,8*S*,10*R*-**5**

Functional	Solvent?		Basis Set		Type of Data	
mPW1PW91	PCM		6-311G(d,p)		Unscaled Shifts	
	Isomer 1	Isomer 2	Isomer 3	Isomer 4	Isomer 5	Isomer 6
sDP4+ (H data)	0.02%	99.98%	-	-	-	-
sDP4+ (C data)	0.54%	99.46%	-	-	-	-
sDP4+ (all data)	0.00%	100.00%	-	-	-	-
uDP4+ (H data)	0.00%	100.00%	-	-	-	-
uDP4+ (C data)	100.00%	0.00%	-	-	-	-
uDP4+ (all data)	83.93%	16.07%	-	-	-	-
DP4+ (H data)	0.00%	100.00%	-	-	-	-
DP4+ (C data)	100.00%	0.00%	-	-	-	-
DP4+ (all data)	0.00%	100.00%	-	-	-	-

Functional		Solvent?		Basis Set		Type of Data	
mPW1PW91		PCM		6-311G(d,p)		Unscaled Shifts	
		DP4+	0.00%	100.00%	-	-	-
Nuclei	sp2?	Experimental	Isomer 1	Isomer 2	Isomer 3	Isomer 4	Isomer 5
C		79.5	76.0622	74.2513			
C	x	135.8	135.5422	134.1323			
C	x	129.2	137.2658	135.8851			
C		79.8	75.7858	75.4483			
C		42.5	44.3624	43.8035			
C		22.8	18.672	17.5512			
C	x	155.8	163.688	156.6318			
C		105.3	103.9016	100.2476			
C		43.8	39.2282	38.0503			
C		38.7	33.4624	35.0231			
C	x	124.2	126.5138	121.8617			
C	x	171.1	172.1056	168.8265			
C		8.3	5.415	4.8285			
C		29.2	24.2716	23.5731			
C		62.8	58.5036	58.9896			
C		20.8	17.287	17.0932			
C	x	170.7	172.532	170.5733			
C		59.1	54.7042	52.7385			
C		15.1	10.9158	10.8119			
H		4.2	3.9644	4.0166			
H	x	6.74	7.0112	6.9858			
H	x	6.39	7.07	6.8478			
H		2.53	1.60	1.938			
H		2.47	2.73	2.6221			
H		2.61	2.86	2.8192			
H		1.4	2.1576	2.2344			
H		2.26	3.2894	2.6024			
H		1.79	1.945733	1.78239962			
H		1.61	0.674466	1.18653329			
H		4.37	3.6628	3.9216			
H		4.43	4.8826	4.4979			
H		2.17	2.2174662	2.17953314			
H		3.15	3.3732	3.0254			
H		3.46	3.6174	3.5878			
H		1.21	1.27806666	1.23139956			

Table S8 Energy analyses of 1*R*,7*R*,8*R*,10*S*,12*R*-**6** (six conformers)

NO.	3D conformers B3LYP/6-31G(d,p)	E (Hartree)	ΔE (KJ/mol)	Boltzmann distribution
1*R*,7*R*,8*R*,10*S*,12*R*-**6** (conformer 1)	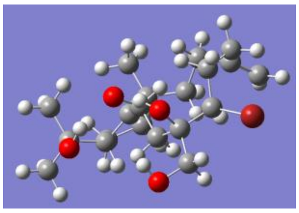	-3654.063991	5.243121193	9.99%
1*R*,7*R*,8*R*,10*S*,12*R*-**6** (conformer 2)	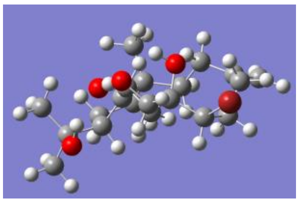	-3654.016987	128.6520689	0.00%
1*R*,7*R*,8*R*,10*S*,12*R*-**6** (conformer 3)	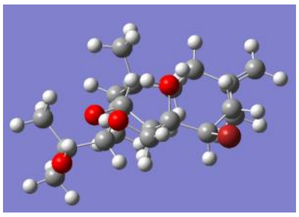	-3654.063212	7.288384793	4.38%
1*R*,7*R*,8*R*,10*S*,12*R*-**6** (conformer 4)	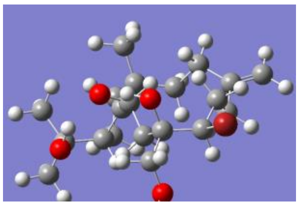	-3654.060185	15.2357698	0.18%

1*R*,7*R*,8*R*,10*S*,12*R*-**6** (conformer 5)	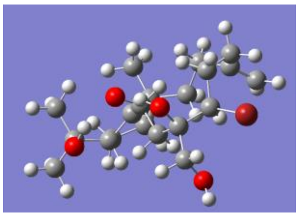	-3654.065988	0	82.81%
1*R*,7*R*,8*R*,10*S*,12*R*-**6** (conformer 6)	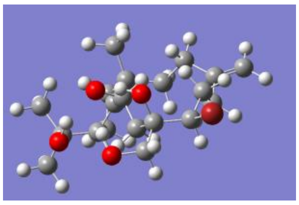	-3654.062738	8.532871245	2.65%

Table S9 Cartesian coordinates of the low-energy re-optimized conformers of 1*R*,7*R*,8*R*,10*S*,12*R*-**6** calculated at B3LYP/6-31G(d,p) level of theory

1*R*,7*R*,8*R*,10*S*,12*R*-**6** (conformer 1)							
Atomic	Standard Orientation (Å)			Atomic	Standard Orientation (Å)		
Type	X	Y	Z	Type	X	Y	Z
C	2.119118	0.819963	1.209604	H	-1.779646	3.858751	-0.684575
C	2.324363	2.32985	0.937619	H	2.223916	0.46707	-0.939718
C	1.302484	2.958018	0.000753	H	0.138285	0.969146	-0.420426
C	-0.131595	3.081214	0.523357	H	-0.395416	-2.03342	-0.802196
C	-1.27458	2.897543	-0.526553	H	0.482626	-0.967847	-1.907779
C	2.382829	-0.082604	-0.011045	H	-4.12088	2.646917	-1.307613
C	-2.383359	1.861369	-0.191344	H	-2.913804	1.827079	-2.302317
C	-1.912798	0.379138	-0.167606	H	-4.619893	0.107473	-2.086865
C	-0.479181	0.085908	-0.335052	H	-4.933655	0.527329	-0.410948
C	0.225893	-1.137586	-0.853585	H	-2.638102	-1.060013	-1.573805
C	1.547243	-1.404724	-0.060772	H	0.974002	3.883014	-1.890391
C	-3.429611	1.796904	-1.332704	H	2.704016	3.344672	-1.540958
C	-4.141089	0.44191	-1.161658	H	-4.768272	-3.447274	0.142737
C	-3.039193	-0.54569	-0.691654	H	-4.057301	-2.979248	-1.410526
C	1.672745	3.411775	-1.203746	H	-5.422105	-2.052164	-0.74242
C	-3.509435	-1.695235	0.253833	H	-4.37085	-2.090706	2.188046
C	-4.504497	-2.592853	-0.488546	H	-3.400614	-0.610027	2.150433
C	-4.115312	-1.214788	1.583213	H	-5.028483	-0.626627	1.442259
O	-2.388657	-2.544135	0.544723	H	-1.76005	-1.98873	1.037367
C	-3.06806	2.238956	1.138514	H	-2.384669	2.133061	1.985847
O	-1.104523	-0.020226	0.983827	H	-3.404695	3.281869	1.099007
C	2.281255	-2.595915	-0.708096	H	-3.942312	1.616623	1.34627
O	1.240376	-1.882372	1.256456	H	2.612228	-2.340805	-1.718889
O	1.431503	-3.72259	-0.816288	H	3.16906	-2.819886	-0.104663
Br	4.378164	-0.366482	-0.096317	H	0.512859	-1.348754	1.614611
H	1.100705	0.664821	1.576118	H	1.097119	-3.882862	0.082739
H	2.775682	0.503877	2.024522				
H	3.333837	2.484692	0.540506				
H	2.283375	2.845129	1.908192				
H	-0.228921	4.069079	0.994626				
H	-0.26628	2.368192	1.342011				
H	-0.840411	2.632096	-1.497034				

1*R*,7*R*,8*R*,10*S*,12*R*-**6** (conformer 2)							
Atomic	Standard Orientation (Å)			Atomic	Standard Orientation (Å)		
Type	X	Y	Z	Type	X	Y	Z
C	2.659352	0.994019	-1.216181	H	-0.885992	3.655686	-0.200797
C	2.951429	1.939896	-0.006475	H	2.463075	-1.038049	-1.902749
C	1.883223	2.980733	0.282362	H	-0.528714	-0.077124	1.407973
C	0.565911	2.50069	0.890134	H	-0.26872	-1.270115	-1.343165
C	-0.616437	2.595816	-0.103426	H	0.188053	0.401375	-1.024506
C	2.567268	-0.520168	-0.94487	H	-3.437003	3.355144	-0.465996
C	-1.958033	1.861033	0.198848	H	-2.586517	2.52871	-1.773056
C	-2.045213	0.3462	0.036409	H	-4.679619	1.308104	-1.774205
C	-1.04296	-0.597425	0.575459	H	-4.806758	1.365475	-0.01973
C	0.080152	-0.578718	-0.569512	H	-2.858625	-0.276041	-1.708489
C	1.4701	-1.05288	0.013029	H	1.356684	5.040044	0.286128
C	-3.041402	2.384923	-0.783939	H	3.031057	4.63817	-0.382133
C	-4.096734	1.282325	-0.850641	H	-5.81029	-2.381434	-1.127553
C	-3.282656	-0.023618	-0.714003	H	-5.017269	-1.368602	-2.340537
C	2.098559	4.280171	0.047421	H	-6.044603	-0.622264	-1.087804
C	-4.094663	-1.347628	-0.364243	H	-5.154999	-2.231178	1.297819
C	-5.319207	-1.417131	-1.288314	H	-3.644356	-1.42689	1.757336
C	-4.526765	-1.352984	1.115447	H	-5.107463	-0.461905	1.385777
O	-3.313833	-2.467849	-0.672453	H	-2.524184	-2.425615	-0.048274
C	-2.411177	2.127934	1.670508	H	-1.685652	1.738074	2.388301
O	-1.415476	-1.840982	0.926598	H	-2.498528	3.211331	1.811891
C	1.48016	-2.613122	0.139078	H	-3.383675	1.678738	1.895656
O	1.681684	-0.538265	1.310808	H	0.964718	-3.065949	-0.719363
O	0.897234	-2.985191	1.369601	H	2.512458	-2.967471	0.15416
Br	4.380223	-1.099531	-0.308892	H	1.486557	-1.311932	1.893766
H	3.457452	1.116106	-1.954843	H	-0.07129	-2.714999	1.300437
H	1.747888	1.311508	-1.736123				
H	3.894435	2.460536	-0.200247				
H	3.110745	1.329079	0.886727				
H	0.332478	3.115272	1.768212				
H	0.708359	1.483906	1.25525				
H	-0.289784	2.303799	-1.107619				

1*R*,7*R*,8*R*,10*S*,12*R*-**6** (conformer 3)							
Atomic	Standard Orientation (Å)			Atomic	Standard Orientation (Å)		
Type	X	Y	Z	Type	X	Y	Z
C	2.301052	1.37567	-0.137433	H	-2.402785	3.570023	-0.179323
C	1.284102	2.05842	-1.090822	H	2.640455	-0.196729	-1.610377
C	0.712649	3.345712	-0.525528	H	0.052068	0.589932	1.128676
C	-0.388581	3.206484	0.509258	H	-0.192869	-2.039956	-0.289747
C	-1.739069	2.701385	-0.089418	H	0.230476	-0.73696	-1.353865
C	2.64836	-0.077806	-0.523477	H	-4.626319	1.008302	0.762689
C	-2.53205	1.586258	0.669819	H	-4.374412	2.487933	-0.161419
C	-1.948569	0.182171	0.32772	H	-3.413686	1.237678	-2.037484
C	-0.529781	-0.136863	0.57823	H	-4.749576	0.215779	-1.543949
C	0.289994	-1.059725	-0.306174	H	-2.089586	-0.646433	-1.674895
C	1.788168	-1.198352	0.11512	H	0.766496	5.466507	-0.508434
C	-3.951517	1.499871	0.052043	H	1.937968	4.640949	-1.67638
C	-3.796252	0.633193	-1.2071	H	-4.327369	-3.492201	-1.586979
C	-2.763054	-0.463793	-0.829012	H	-3.127706	-2.59987	-2.536671
C	1.159611	4.541315	-0.923203	H	-4.736881	-1.89874	-2.253172
C	-3.402631	-1.863781	-0.51257	H	-4.868304	-2.81782	0.749991
C	-3.935398	-2.49348	-1.804445	H	-4.138416	-1.385751	1.491555
C	-4.513448	-1.801616	0.550247	H	-5.369645	-1.201278	0.2247
O	-2.388208	-2.774317	-0.056872	H	-2.046694	-2.366213	0.762849
C	-2.594009	1.859361	2.177618	H	-1.60872	1.829605	2.652345
O	-1.562703	-0.691263	1.429992	H	-3.028007	2.849365	2.363976
C	2.269142	-2.607701	-0.31836	H	-3.216077	1.114297	2.682798
O	1.915847	-1.104348	1.524028	H	2.069586	-2.777918	-1.38579
O	1.658029	-3.605849	0.498794	H	3.340506	-2.698307	-0.14196
Br	4.582684	-0.36474	-0.054195	H	1.716695	-1.999142	1.858828
H	1.967209	1.392301	0.90392	H	0.76531	-3.787046	0.164398
H	3.21596	1.971617	-0.164193				
H	0.459418	1.378171	-1.326659				
H	1.791148	2.263051	-2.041622				
H	-0.549937	4.175754	0.992756				
H	-0.046176	2.540238	1.307221				
H	-1.570129	2.364647	-1.119561				

1*R*,7*R*,8*R*,10*S*,12*R*-**6** (conformer 4)							
Atomic	Standard Orientation (Å)			Atomic	Standard Orientation (Å)		
Type	X	Y	Z	Type	X	Y	Z
C	2.288735	1.333555	-0.126005	H	-2.398753	3.503108	-0.454375
C	1.306178	1.930251	-1.168793	H	2.732142	-0.355969	-1.442904
C	0.719509	3.259928	-0.73245	H	0.031291	0.661281	1.067196
C	-0.396346	3.209185	0.294693	H	-0.198659	-2.108413	-0.111152
C	-1.736192	2.647453	-0.276203	H	0.243208	-0.896598	-1.265601
C	2.654473	-0.14097	-0.376584	H	-4.644103	1.063437	0.699013
C	-2.541224	1.603234	0.567582	H	-4.367241	2.454112	-0.3472
C	-1.968782	0.169394	0.340746	H	-3.409556	1.03325	-2.101188
C	-0.546061	-0.123184	0.596908	H	-4.76777	0.080223	-1.534562
C	0.27602	-1.125693	-0.195089	H	-2.133481	-0.8512	-1.567824
C	1.762529	-1.236597	0.264558	H	0.761603	5.374038	-0.906831
C	-3.959314	1.482406	-0.0473	H	1.955453	4.453759	-1.977433
C	-3.809943	0.50878	-1.225906	H	-4.442322	-3.615573	-1.200787
C	-2.800738	-0.569411	-0.74503	H	-3.241701	-2.837586	-2.244366
C	1.166206	4.417693	-1.229933	H	-4.834825	-2.082846	-2.005136
C	-3.471706	-1.915737	-0.292525	H	-4.94438	-2.712784	1.066287
C	-4.036555	-2.648631	-1.51508	H	-4.171845	-1.238029	1.667161
C	-4.568083	-1.72799	0.771047	H	-5.413917	-1.138709	0.40084
O	-2.474992	-2.804127	0.230116	H	-2.095531	-2.329942	0.994182
C	-2.606669	1.995805	2.048668	H	-1.624075	1.993591	2.529811
O	-1.565525	-0.602651	1.51379	H	-3.030785	3.001918	2.153822
C	2.271311	-2.639383	-0.121251	H	-3.239571	1.299988	2.607924
O	1.865847	-1.0891	1.679633	H	3.303944	-2.754866	0.223281
O	2.160086	-2.782223	-1.533592	H	1.645614	-3.380767	0.403794
Br	4.550767	-0.384484	0.260913	H	1.184432	-1.646173	2.089917
H	1.920244	1.439598	0.898541	H	2.644733	-3.578424	-1.79578
H	3.202995	1.929588	-0.172224				
H	0.489842	1.230896	-1.376071				
H	1.845749	2.054682	-2.115266				
H	-0.567985	4.217141	0.68711				
H	-0.063886	2.619259	1.154835				
H	-1.551136	2.219242	-1.268859				

1*R*,7*R*,8*R*,10*S*,12*R*-**6** (conformer 5)							
Atomic	Standard Orientation (Å)			Atomic	Standard Orientation (Å)		
Type	X	Y	Z	Type	X	Y	Z
C	-2.018357	0.805505	-1.284758	H	1.838992	3.756181	0.809724
C	-2.216813	2.312392	-0.99123	H	-2.198128	0.408641	0.856125
C	-1.221681	2.909697	-0.006957	H	-0.08583	0.887533	0.395643
C	0.229012	3.031084	-0.479895	H	0.430597	-2.126741	0.620317
C	1.334813	2.804301	0.600506	H	-0.452567	-1.133768	1.798121
C	-2.324083	-0.11625	-0.09108	H	4.153083	2.503985	1.461769
C	2.447301	1.773804	0.262084	H	2.911051	1.653495	2.385378
C	1.96813	0.297194	0.166411	H	4.612455	-0.06817	2.155605
C	0.52938	0.004971	0.286016	H	4.983646	0.416301	0.509267
C	-0.19146	-1.234524	0.737405	H	2.646171	-1.20437	1.531196
C	-1.521381	-1.460291	-0.05216	H	-0.946781	3.783887	1.916222
C	3.456814	1.658175	1.431862	H	-2.669393	3.271692	1.49793
C	4.16614	0.306277	1.229444	H	4.821005	-3.524271	-0.22181
C	3.075212	-0.654595	0.684251	H	4.069487	-3.117796	1.329823
C	-1.627215	3.337236	1.195658	H	5.453855	-2.16901	0.73759
C	3.569756	-1.764693	-0.294898	H	4.486024	-2.081597	-2.218496
C	4.542238	-2.695531	0.436698	H	3.515518	-0.602249	-2.14693
C	4.213649	-1.231098	-1.58539	H	5.122526	-0.650596	-1.393821
O	2.456306	-2.597772	-0.651914	H	1.842776	-2.022788	-1.14177
C	3.175645	2.197593	-1.030348	H	2.51889	2.127965	-1.902175
O	1.187236	-0.050969	-1.019014	H	3.515657	3.236306	-0.940482
C	-2.278839	-2.615686	0.63699	H	4.053172	1.578727	-1.234362
O	-1.265474	-1.946495	-1.366396	H	-3.116422	-2.909533	-0.005765
O	-2.697877	-2.297126	1.95257	H	-1.593106	-3.464542	0.711573
Br	-4.335055	-0.387066	-0.086847	H	-0.543403	-1.42306	-1.748466
H	-0.991055	0.64456	-1.623781	H	-3.517779	-1.781168	1.863853
H	-2.652227	0.510744	-2.125366				
H	-3.237766	2.47122	-0.625935				
H	-2.136908	2.845591	-1.949559				
H	0.35227	4.030327	-0.920158				
H	0.382695	2.338958	-1.312971				
H	0.867325	2.506394	1.545748				

1*R*,7*R*,8*R*,10*S*,12*R*-**6** (conformer 6)							
Atomic	Standard Orientation (Å)			Atomic	Standard Orientation (Å)		
Type	X	Y	Z	Type	X	Y	Z
C	2.29113	1.369772	0.147809	H	-2.400185	3.560229	-0.112455
C	1.258383	2.037489	-1.092014	H	2.604335	-0.219213	-1.611614
C	0.699564	3.326553	-0.520277	H	0.03472	0.525782	1.181673
C	-0.37729	3.181427	0.537464	H	-0.12828	-2.119193	-0.20335
C	-1.737365	2.688698	-0.045919	H	0.222594	-0.827483	-1.316444
C	2.641069	-0.08253	-0.528031	H	-4.616221	0.984633	0.79972
C	-2.522403	1.564271	0.705895	H	-4.370971	2.493911	-0.077797
C	-1.953194	0.159559	0.334782	H	-3.414801	1.301842	-1.998017
C	-0.54171	-0.186924	0.605115	H	-4.755082	0.272309	-1.534424
C	0.296516	-1.114865	-0.259354	H	-2.101975	-0.610347	-1.692386
C	1.807121	-1.195555	0.153603	H	0.751762	5.447179	-0.506224
C	-3.947118	1.49908	0.100132	H	1.894661	4.621681	-1.702474
C	-3.79891	0.67331	-1.186462	H	-4.391492	-3.416993	-1.68987
C	-2.773931	-0.442634	-0.842108	H	-3.189071	-2.508187	-2.618889
C	1.135581	4.522056	-0.92982	H	-4.787543	-1.796778	-2.297481
C	-3.434805	-1.841416	-0.567336	H	-4.907407	-2.815053	0.670393
C	-3.989426	-2.416097	-1.876832	H	-4.150867	-1.421889	1.457438
C	-4.538894	-1.797503	0.50466	H	-5.388661	-1.173655	0.207174
O	-2.437042	-2.783802	-0.159159	H	-2.07347	-2.424347	0.672175
C	-2.565561	1.814668	2.2179	H	-1.574756	1.772596	2.680606
O	-1.601643	-0.742093	1.423358	H	-2.991202	2.804408	2.424405
C	2.308869	-2.600003	-0.241092	H	-3.185761	1.065333	2.718761
O	1.956337	-1.047226	1.557252	H	2.114303	-2.79012	-1.307578
O	1.587637	-3.516822	0.58732	H	3.383371	-2.672505	-0.054592
Br	4.596668	-0.342294	-0.115053	H	1.688291	-1.905156	1.93437
H	1.965936	1.388837	0.896017	H	2.049156	-4.36748	0.575645
H	3.200827	1.972673	-0.187344				
H	0.431194	1.351638	-1.303083				
H	1.747052	2.234784	-2.053976				
H	-0.525285	4.144748	1.03696				
H	-0.02237	2.501078	1.317831				
H	-1.584274	2.368463	-1.083471				

Table S10 Energy analyses of 1*R*,7*R*,8*S*,10*S*,12*R*-**6** (eight conformers)

NO.	**3D conformers** B3LYP/6-31G(d,p)	**E (Hartree)**	**ΔE (KJ/mol)**	**Boltzmann distribution**
1*R*,7*R*,8*S*,10*S*,12*R*-**6** (conformer 1)	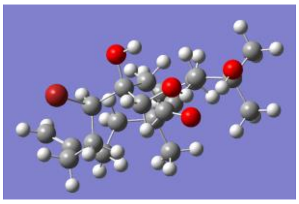	-3654.064038	0.270426381	45.59%
1*R*,7*R*,8*S*,10*S*,12*R*-**6** (conformer 2)	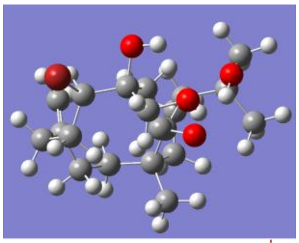	-3654.059541	12.07729469	0.39%
1*R*,7*R*,8*S*,10*S*,12*R*-**6** (conformer 3)	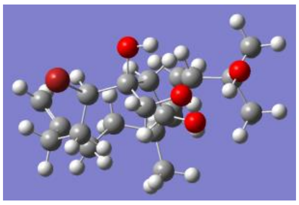	-3654.059542	12.07466919	0.39%
1*R*,7*R*,8*S*,10*S*,12*R*-**6** (conformer 4)	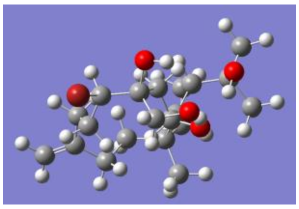	-3654.061290	7.485297206	2.48%

1*R*,7*R*,8*S*,10*S*,12*R*-**6** (conformer 5)	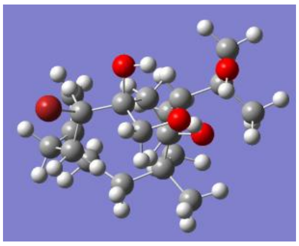	-3654.059215	12.93320731	0.28%
1*R*,7*R*,8*S*,10*S*,12*R*-**6** (conformer 6)	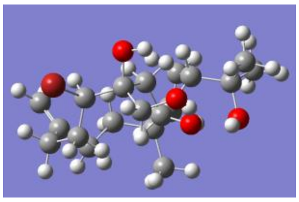	-3654.053768	27.23429952	0.00%
1*R*,7*R*,8*S*,10*S*,12*R*-**6** (conformer 7)	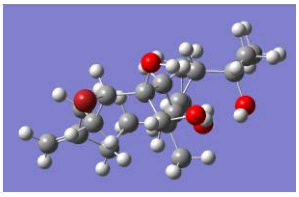	-3654.056647	19.67548834	0.02%
1*R*,7*R*,8*S*,10*S*,12*R*-**6** (conformer 8)	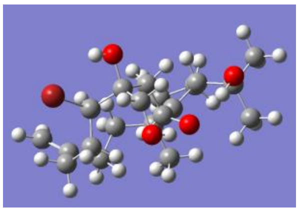	-3654.064141	0	50.85%

Table S11 Cartesian coordinates of the low-energy re-optimized conformers of 1*R*,7*R*,8*S*,10*S*,12*R*-**6** calculated at B3LYP/6-31G(d,p) level of theory.

1*R*,7*R*,8*S*,10*S*,12*R*-**6** (conformer 1)							
Atomic	Standard Orientation (Å)			Atomic	Standard Orientation (Å)		
Type	X	Y	Z	Type	X	Y	Z
C	2.076147	0.801367	1.119422	H	-1.993835	3.915337	-0.421708
C	2.212506	2.338556	0.952384	H	2.403813	0.618035	-1.038916
C	1.16328	3.019355	0.081804	H	0.10288	1.130389	-0.446006
C	-0.259492	3.114993	0.64822	H	-0.375715	-1.810226	-1.143132
C	-1.439728	2.969683	-0.372164	H	0.463183	-0.589661	-2.099991
C	2.443417	-0.011749	-0.147433	H	-4.306977	2.558944	-1.119427
C	-2.482542	1.851121	-0.085736	H	-3.069607	1.883487	-2.183679
C	-1.91044	0.414872	-0.160537	H	-4.647908	0.035025	-2.012706
C	-0.471737	0.217942	-0.393236	H	-4.929425	0.341829	-0.306425
C	0.24147	-0.911517	-1.075162	H	-2.55734	-0.985701	-1.636462
C	1.61663	-1.298134	-0.439171	H	0.787003	4.07047	-1.732936
C	-3.553741	1.767634	-1.204364	H	2.525277	3.512761	-1.465755
C	-4.160948	0.352544	-1.085583	H	-4.249652	-3.744995	-0.023766
C	-2.970295	-0.570907	-0.70823	H	-3.51998	-3.142523	-1.522884
C	1.50338	3.555508	-1.097543	H	-5.048739	-2.44758	-0.937396
C	-3.288438	-1.818427	0.182658	H	-4.140471	-2.408221	2.0706
C	-4.077684	-2.848132	-0.62748	H	-3.455344	-0.776709	2.09875
C	-4.024658	-1.487288	1.490719	H	-5.022111	-1.070377	1.316949
O	-2.044388	-2.466808	0.536665	H	-1.547968	-1.775003	1.018702
C	-3.158059	2.104522	1.278337	H	-2.448321	1.997345	2.103625
O	-1.042159	0.006422	0.935347	H	-3.564612	3.122514	1.307605
C	1.441149	-2.197866	0.805678	H	-3.984326	1.413736	1.467453
O	0.87575	-3.452473	0.427783	H	0.845959	-1.708683	1.584763
O	2.29917	-2.063556	-1.422093	H	2.433789	-2.416303	1.210166
Br	4.396272	-0.440501	-0.030921	H	-0.092483	-3.355416	0.360765
H	1.0656	0.561442	1.45587	H	1.973267	-2.974373	-1.290289
H	2.738372	0.490171	1.932078				
H	3.212281	2.555266	0.558324				
H	2.17046	2.777852	1.959566				
H	-0.344312	4.084146	1.158559				
H	-0.365704	2.369101	1.441129				
H	-1.034842	2.812299	-1.378299				

1*R*,7*R*,8*S*,10*S*,12*R*-**6** (conformer 2)							
Atomic	Standard Orientation (Å)			Atomic	Standard Orientation (Å)		
Type	X	Y	Z	Type	X	Y	Z
C	2.50924	1.511507	0.346047	H	-2.505186	3.654086	0.607604
C	1.951762	2.665814	-0.531629	H	2.723666	0.309601	-1.471866
C	0.471306	2.657347	-0.874842	H	0.037213	0.753108	1.018673
C	-0.455874	3.144326	0.234768	H	-0.09239	-1.75299	-0.710627
C	-1.964046	2.814236	0.153146	H	0.399495	-0.264033	-1.477616
C	2.7505	0.167133	-0.390291	H	-4.571067	0.804067	1.143132
C	-2.542183	1.538863	0.858164	H	-4.523879	2.38201	0.356234
C	-1.928383	0.216116	0.319133	H	-3.731745	1.396892	-1.742549
C	-0.482984	-0.008222	0.450207	H	-4.922166	0.230968	-1.2062
C	0.377392	-0.786	-0.515611	H	-2.232726	-0.395585	-1.73403
C	1.848452	-1.050855	-0.071529	H	-0.97898	2.373544	-2.416012
C	-4.020335	1.410109	0.414247	H	0.777979	2.106912	-2.89413
C	-3.964117	0.686367	-0.941302	H	-4.289129	-3.380374	-1.764397
C	-2.822112	-0.361633	-0.810522	H	-3.251724	-2.306479	-2.716132
C	0.064232	2.363462	-2.115101	H	-4.858314	-1.753646	-2.18883
C	-3.344166	-1.827585	-0.601393	H	-4.604652	-3.01958	0.679285
C	-3.980256	-2.338972	-1.89923	H	-3.879573	-1.636164	1.512383
C	-4.332521	-1.964825	0.570696	H	-5.252761	-1.39245	0.413028
O	-2.238637	-2.710232	-0.372056	H	-1.832693	-2.385064	0.451456
C	-2.410794	1.669107	2.381997	H	-2.860915	0.812261	2.890712
O	-1.400856	-0.727583	1.321696	H	-1.367334	1.728153	2.710625
C	1.931737	-1.506337	1.399967	H	-2.918725	2.578525	2.724322
O	1.17941	-2.703988	1.58849	H	1.603601	-0.72208	2.094291
O	2.328725	-2.110603	-0.89381	H	2.97089	-1.751013	1.62754
Br	4.676319	-0.354358	-0.102752	H	0.249735	-2.450115	1.706901
H	1.889456	1.364418	1.236044	H	1.958458	-2.922402	-0.501656
H	3.473001	1.853367	0.727953				
H	2.541449	2.703884	-1.455432				
H	2.168904	3.599697	0.006988				
H	-0.358963	4.241297	0.240407				
H	-0.051936	2.842539	1.209824				
H	-2.27039	2.818683	-0.897705				

1*R*,7*R*,8*S*,10*S*,12*R*-**6** (conformer 3)							
Atomic	Standard Orientation (Å)			Atomic	Standard Orientation (Å)		
Type	X	Y	Z	Type	X	Y	Z
C	2.509272	1.511517	0.34596	H	-2.505227	3.654088	0.607421
C	1.951777	2.665854	-0.531684	H	2.723873	0.309612	-1.471914
C	0.471328	2.657489	-0.874907	H	0.03721	0.753284	1.018332
C	-0.455873	3.144344	0.234748	H	-0.092349	-1.752875	-0.710963
C	-1.964038	2.814202	0.153088	H	0.399523	-0.263814	-1.477798
C	2.75055	0.167143	-0.390336	H	-4.571016	0.804175	1.143476
C	-2.542125	1.538889	0.858234	H	-4.523872	2.38206	0.356474
C	-1.928409	0.216119	0.319136	H	-3.732181	1.396814	-1.742367
C	-0.482987	-0.008157	0.450009	H	-4.922402	0.23084	-1.205706
C	0.377375	-0.785885	-0.515851	H	-2.232944	-0.395482	-1.733986
C	1.848423	-1.050816	-0.071677	H	-0.978945	2.374021	-2.416147
C	-4.02035	1.410147	0.414486	H	0.778008	2.107362	-2.894276
C	-3.964331	0.68632	-0.941031	H	-4.288958	-3.38047	-1.764432
C	-2.822237	-0.361608	-0.810416	H	-3.251868	-2.306274	-2.716162
C	0.064261	2.363826	-2.115221	H	-4.8585	-1.753807	-2.188585
C	-3.344167	-1.827652	-0.601337	H	-4.604497	-3.019825	0.679345
C	-3.98029	-2.338991	-1.899168	H	-3.879467	-1.636419	1.512489
C	-4.332444	-1.965044	0.570803	H	-5.252739	-1.392732	0.41323
O	-2.238571	-2.710213	-0.372166	H	-1.832538	-2.385034	0.451303
C	-2.410552	1.669153	2.382029	H	-2.86078	0.812401	2.890814
O	-1.400707	-0.727595	1.32163	H	-1.367054	1.728012	2.710572
C	1.931568	-1.506301	1.399828	H	-2.91829	2.578661	2.724404
O	1.179342	-2.704054	1.588136	H	1.60318	-0.722099	2.094077
O	2.328723	-2.110566	-0.893902	H	2.970728	-1.750797	1.627585
Br	4.676296	-0.354477	-0.102566	H	0.24967	-2.450302	1.706821
H	1.889523	1.364447	1.235986	H	1.958479	-2.922361	-0.501712
H	3.473042	1.853403	0.727827				
H	2.541465	2.70395	-1.455488				
H	2.168981	3.599699	0.006977				
H	-0.35898	4.241314	0.240484				
H	-0.051924	2.84247	1.20977				
H	-2.27034	2.818496	-0.897773				

1*R*,7*R*,8*S*,10*S*,12*R*-**6** (conformer 4)							
Atomic	Standard Orientation (Å)			Atomic	Standard Orientation (Å)		
Type	X	Y	Z	Type	X	Y	Z
C	2.380173	1.296101	-0.125001	H	-2.269689	3.586901	-0.080662
C	1.400913	2.068097	-1.051601	H	2.71992	-0.189747	-1.682571
C	0.844099	3.330797	-0.418464	H	0.093049	0.57701	0.938905
C	-0.267853	3.161944	0.602192	H	-0.203235	-2.039316	-0.559001
C	-1.625398	2.70181	-0.019316	H	0.265713	-0.687301	-1.541953
C	2.68906	-0.141346	-0.591923	H	-4.564825	1.090127	0.813786
C	-2.448554	1.576244	0.696525	H	-4.264171	2.592891	-0.05533
C	-1.924631	0.171615	0.260967	H	-3.398694	1.377964	-2.000655
C	-0.498183	-0.159459	0.409853	H	-4.773765	0.401987	-1.520078
C	0.291785	-1.065954	-0.513167	H	-2.176008	-0.597636	-1.7492
C	1.781636	-1.304154	-0.115383	H	0.930826	5.446879	-0.289015
C	-3.883115	1.578875	0.10824	H	2.100125	4.665097	-1.488559
C	-3.795004	0.758969	-1.187492	H	-4.566408	-3.315309	-1.655767
C	-2.811199	-0.402155	-0.877552	H	-3.393691	-2.430852	-2.644037
C	1.313651	4.538478	-0.74814	H	-4.952621	-1.680711	-2.228809
C	-3.509261	-1.775376	-0.577494	H	-4.946011	-2.706316	0.734003
C	-4.148832	-2.323992	-1.858202	H	-4.10287	-1.343113	1.484942
C	-4.551469	-1.70159	0.551775	H	-5.393169	-1.04723	0.300631
O	-2.517146	-2.749557	-0.227601	H	-2.098747	-2.401702	0.580479
C	-2.471265	1.764412	2.218505	H	-1.480596	1.666353	2.672345
O	-1.475339	-0.748743	1.312185	H	-2.859236	2.75909	2.469379
C	1.903592	-1.621262	1.391794	H	-3.116769	1.018924	2.692962
O	1.124242	-2.772256	1.714515	H	1.627001	-0.764706	2.019904
O	2.224165	-2.444613	-0.84534	H	2.940337	-1.880173	1.608808
Br	4.597503	-0.550184	-0.107713	H	0.202525	-2.487923	1.825375
H	2.027779	1.287198	0.912063	H	1.866139	-3.212061	-0.363116
H	3.316409	1.858243	-0.104422				
H	0.569742	1.424025	-1.355622				
H	1.937038	2.321827	-1.973593				
H	-0.418206	4.113469	1.123018				
H	0.060622	2.463369	1.379079				
H	-1.459367	2.398195	-1.060093				

1*R*,7*R*,8*S*,10*S*,12*R*-**6** (conformer 5)							
Atomic	Standard Orientation (Å)			Atomic	Standard Orientation (Å)		
Type	X	Y	Z	Type	X	Y	Z
C	1.898832	1.250355	0.845136	H	-0.611983	2.050086	2.060167
C	1.632729	2.625016	0.198208	H	1.446372	0.237511	-1.030529
C	0.316029	2.73604	-0.55121	H	-0.248315	-0.318295	2.147126
C	-0.9036	3.201721	0.227234	H	0.323434	-2.64992	0.985533
C	-1.445588	2.370632	1.424453	H	0.029881	-1.849469	-0.52604
C	2.09266	0.112799	-0.161143	H	-4.471512	0.986568	0.50609
C	-2.43456	1.171411	1.233581	H	-3.801485	2.589416	0.230376
C	-1.808142	-0.118428	0.613165	H	-2.44434	1.785458	-1.62706
C	-0.615249	-0.767519	1.221749	H	-3.902145	0.840782	-1.828612
C	0.373112	-1.66634	0.493699	H	-1.323348	-0.230702	-1.498235
C	1.892316	-1.311749	0.401368	H	-0.635239	2.667967	-2.454522
C	-3.556288	1.520825	0.225031	H	1.178173	2.319709	-2.444511
C	-3.068348	1.029044	-1.14685	H	-3.573817	-2.609248	-3.06651
C	-2.226685	-0.248541	-0.878297	H	-2.112011	-1.629308	-3.25968
C	0.280209	2.557829	-1.878954	H	-3.708257	-0.845528	-3.212986
C	-2.980141	-1.571259	-1.27083	H	-4.804627	-2.661183	-0.90242
C	-3.104315	-1.657758	-2.797345	H	-4.291203	-1.680619	0.478951
C	-4.366756	-1.700267	-0.613334	H	-5.055444	-0.90787	-0.925334
O	-2.193386	-2.712746	-0.915852	H	-2.115351	-2.673544	0.05503
C	-3.025589	0.873099	2.62585	H	-2.246428	0.6368	3.360117
O	-1.926617	-1.330399	1.424531	H	-3.574367	1.748373	2.992464
C	2.641256	-1.506329	1.740485	H	-3.713892	0.025117	2.592992
O	2.763219	-2.895864	2.053615	H	2.166357	-0.958046	2.564246
O	2.433969	-2.238099	-0.528777	H	3.664539	-1.141134	1.621143
Br	3.925825	0.321554	-0.941473	H	1.921023	-3.202282	2.424005
H	1.068049	1.005318	1.508461	H	2.69747	-3.011267	0.00462
H	2.789242	1.324826	1.478527				
H	2.463383	2.843143	-0.481299				
H	1.664611	3.383054	0.99217				
H	-1.719703	3.417509	-0.469426				
H	-0.627864	4.17689	0.657553				
H	-2.00386	3.088865	2.039042				

1*R*,7*R*,8*S*,10*S*,12*R*-**6** (conformer 6)							
Atomic	Standard Orientation (Å)			Atomic	Standard Orientation (Å)		
Type	X	Y	Z	Type	X	Y	Z
C	2.56698	1.460846	0.42954	H	-2.448596	3.63884	0.714879
C	2.036594	2.682825	-0.373956	H	2.877127	0.383412	-1.45169
C	0.565575	2.740111	-0.755544	H	0.080477	0.802713	0.826778
C	-0.392542	3.151733	0.360724	H	-0.047599	-1.643181	-0.999929
C	-1.899857	2.833339	0.210416	H	0.507907	-0.107973	-1.6357
C	2.817121	0.162466	-0.384474	H	-4.575314	0.801053	0.920673
C	-2.519695	1.512537	0.789246	H	-4.451127	2.420054	0.204919
C	-1.891685	0.208904	0.202641	H	-3.412032	1.455839	-1.838713
C	-0.435899	0.031705	0.272376	H	-4.790151	0.42422	-1.520976
C	0.427505	-0.701105	-0.719311	H	-2.271685	-0.605736	-1.793792
C	1.862895	-1.04686	-0.220536	H	-0.838328	2.640437	-2.361202
C	-3.974903	1.434275	0.259628	H	0.926695	2.36908	-2.806713
C	-3.836008	0.754728	-1.10878	H	-3.031568	-3.875971	-0.326469
C	-2.830944	-0.401576	-0.872672	H	-1.61147	-2.848537	-0.098148
C	0.193415	2.572479	-2.02998	H	-2.291443	-3.064516	-1.719585
C	-3.496455	-1.765179	-0.429772	H	-5.126276	-3.068045	-0.984289
C	-2.542387	-2.954069	-0.657725	H	-5.592785	-1.37056	-0.895006
C	-4.792469	-2.048651	-1.201358	H	-4.644136	-1.956834	-2.28303
O	-3.895014	-1.719134	0.947781	H	-3.07606	-1.68625	1.469383
C	-2.467025	1.531803	2.323401	H	-2.959934	0.649578	2.741096
O	-1.291017	-0.72449	1.165802	H	-1.441453	1.547373	2.708757
C	1.832062	-1.618378	1.212715	H	-2.982012	2.422026	2.703519
O	0.971821	-2.754797	1.271103	H	1.532586	-0.861426	1.948516
O	2.359734	-2.055478	-1.097567	H	2.834959	-1.964981	1.468905
Br	4.697927	-0.450034	0.007173	H	0.064016	-2.420978	1.375743
H	1.939306	1.259832	1.30337	H	1.956066	-2.884747	-0.783574
H	3.529238	1.77078	0.841324				
H	2.648759	2.780336	-1.278721				
H	2.258057	3.569144	0.238429				
H	-0.296179	4.245594	0.443716				
H	-0.018455	2.783497	1.325153				
H	-2.1739	2.926718	-0.845084				

1*R*,7*R*,8*S*,10*S*,12*R*-**6** (conformer 7)							
Atomic	Standard Orientation (Å)			Atomic	Standard Orientation (Å)		
Type	X	Y	Z	Type	X	Y	Z
C	2.475368	1.265338	-0.046793	H	-2.149141	3.606357	-0.11043
C	1.562121	2.136691	-0.955826	H	2.875103	-0.121037	-1.683017
C	0.969068	3.340639	-0.24078	H	0.140278	0.650469	0.654407
C	-0.211905	3.107171	0.689374	H	-0.154853	-1.976814	-0.848815
C	-1.528216	2.704767	-0.05248	H	0.394143	-0.586009	-1.752442
C	2.769536	-0.147386	-0.596149	H	-4.568026	1.164089	0.450504
C	-2.428314	1.562689	0.533197	H	-4.114524	2.637333	-0.421724
C	-1.888542	0.15246	0.107057	H	-3.009498	1.277737	-2.184525
C	-0.445695	-0.121494	0.17993	H	-4.557631	0.491874	-1.935585
C	0.349918	-1.013484	-0.74348	H	-2.254348	-0.923656	-1.761471
C	1.802592	-1.312341	-0.259797	H	1.066469	5.435549	0.083409
C	-3.80302	1.612739	-0.191287	H	2.305226	4.74655	-1.102899
C	-3.6177	0.745139	-1.441749	H	-3.552666	-3.774011	0.081808
C	-2.833506	-0.489148	-0.937992	H	-1.999402	-2.927199	0.169607
C	1.470555	4.565955	-0.429419	H	-2.721348	-3.287339	-1.408948
C	-3.712847	-1.654325	-0.334908	H	-5.542598	-2.731451	-0.716354
C	-2.943292	-2.988667	-0.377483	H	-5.705829	-0.986203	-0.918934
C	-5.036627	-1.834278	-1.086359	H	-4.873419	-1.948723	-2.163628
O	-4.097726	-1.364488	1.018323	H	-3.287848	-1.407081	1.553045
C	-2.596775	1.693698	2.052412	H	-1.65394	1.566289	2.592916
O	-1.34473	-0.723397	1.145129	H	-3.000833	2.682049	2.303148
C	1.79486	-1.71179	1.233346	H	-3.295722	0.938846	2.424422
O	0.909819	-2.810766	1.442319	H	1.536103	-0.865978	1.882638
O	2.268384	-2.423601	-1.021143	H	2.793746	-2.055347	1.505281
Br	4.625359	-0.654284	-0.009843	H	0.011173	-2.448649	1.527793
H	2.078005	1.195156	0.971482	H	1.869317	-3.207039	-0.601645
H	3.425588	1.794175	0.054765				
H	0.75248	1.536883	-1.383549				
H	2.161524	2.476722	-1.808248				
H	-0.392115	4.024305	1.260032				
H	0.05807	2.359219	1.443673				
H	-1.292504	2.454647	-1.094143				

1*R*,7*R*,8*S*,10*S*,12*R*-**6** (conformer 8)							
Atomic Type	Standard Orientation (Å) X	Y	Z	Atomic Type	Standard Orientation (Å) X	Y	Z
C	2.166223	0.653562	1.118642	H	-1.736007	3.854785	-0.425067
C	2.378326	2.180235	0.974883	H	2.319392	0.507921	-1.062077
C	1.335693	2.895588	0.129818	H	0.139239	0.921099	-0.338989
C	-0.0838	2.970115	0.699297	H	-0.43272	-2.025576	-0.957504
C	-1.245007	2.877007	-0.340801	H	0.32164	-0.866962	-2.054301
C	2.417435	-0.134295	-0.184037	H	-4.097244	2.741594	-1.105592
C	-2.367733	1.837436	-0.070666	H	-2.905052	1.996132	-2.173546
C	-1.923424	0.348392	-0.165654	H	-4.633537	0.290284	-2.107676
C	-0.491115	0.04397	-0.331495	H	-4.938667	0.563306	-0.399916
C	0.182737	-1.121724	-0.996074	H	-2.663029	-0.941302	-1.704963
C	1.616091	-1.450076	-0.459385	H	0.957758	3.990007	-1.659248
C	-3.418856	1.887108	-1.2086	H	2.695046	3.422213	-1.40817
C	-4.148778	0.534189	-1.157589	H	-4.853551	-3.439624	-0.236837
C	-3.061508	-0.506926	-0.779386	H	-4.108495	-2.849267	-1.731639
C	1.673493	3.457557	-1.038029	H	-5.465511	-1.957859	-1.003102
C	-3.563373	-1.730712	0.048988	H	-4.449559	-2.287312	1.931917
C	-4.563055	-2.537934	-0.785097	H	-3.463028	-0.820187	2.036204
C	-4.17783	-1.364087	1.410263	H	-5.082642	-0.754789	1.313479
O	-2.461346	-2.624725	0.274321	H	-1.853904	-2.139591	0.857483
C	-3.035008	2.120701	1.290515	H	-2.34428	1.941262	2.11936
O	-1.135912	-0.163426	0.95865	H	-3.3586	3.167534	1.334757
C	1.589143	-2.425309	0.74152	H	-3.915423	1.494466	1.458983
O	1.005349	-1.939968	1.939417	H	2.622056	-2.681715	1.000522
O	2.183915	-2.158322	-1.559312	H	1.095639	-3.343005	0.38819
Br	4.398434	-0.557089	-0.230203	H	0.118231	-1.594433	1.74576
H	1.16248	0.453467	1.492101	H	3.132415	-2.277918	-1.371334
H	2.827531	0.268831	1.898211				
H	3.377575	2.366745	0.563836				
H	2.369861	2.606	1.988664				
H	-0.170517	3.913034	1.256774				
H	-0.19955	2.188515	1.45638				
H	-0.826715	2.673217	-1.332851				

Table S12 Experimental and calculated ^{1}H NMR data for compound **6**

No.	**6**, exptl. δ_H [a]	1*R*,7*R*,8*S*,10*R*,12*R*-**6**, calcd. δ_H [b]	1*R*,7*R*,8*S*,10,12*R*-**6**, calcd. δ_H [b]
2	1.25	1.38	1.28
	1.96	2.04	2.06
3	1.63	1.91	1.82
	2.11	2.10	2.08
5	2.28	2.32	2.26
	2.42	2.65	2.47
6	1.9	2.01	2.05
	2.11	2.59	2.81
7	4.04	4.53	4.61
9	2.25	1.94	1.81
	2.25	2.45	2.47
10	2.89	2.53	2.50
12	2.21	2.34	2.35
13	1.63	1.74	1.76
	1.9	1.85	1.82
14	1.76	1.51	1.50
	1.76	1.63	1.62
15	0.86	1.13	1.13
16	4.84	5.36	5.34
	5.03	5.54	5.52
17	3.65	3.34	3.61
	3.87	4.11	3.91
18	1.21	1.15	1.17
19	1.27	1.38	1.39

[a] Recorded in $CDCl_3$ at 600 MHz.

[b] Calculated in $CDCl_3$

Table S13 Experimental and calculated ^{13}C NMR data for compound **6**

No.	**6**, exptl. δ_C [a]	1*R*,7*R*,8*S*,10*R*,12*R*-**6**, calcd. δ_H [b]	1*R*,7*R*,8*S*,10,12*R*-**6**, calcd. δ_H [b]
1	44.6	44.50	44.03
2	42.4	37.97	38.00
3	25.1	21.86	21.45
4	147.4	156.41	155.29
5	35.4	33.49	34.34
6	29.7	30.38	30.53
7	63	76.01	76.58
8	75	77.02	72.27
9	33.3	33.62	33.82
10	54.7	59.19	58.19
11	76.2	77.82	80.65
12	50.2	46.50	45.38
13	27.7	23.61	23.33
14	38.8	35.42	34.95
15	24.1	14.73	14.50
16	113.8	114.99	113.97
17	67	67.08	62.42
18	75.4	71.35	72.18
19	29.6	24.40	24.15
20	26	21.53	21.38

[a] Recorded in $CDCl_3$ at 600 MHz.

[b] Calculated in $CDCl_3$

Table S 14 DP4+ analyses of calculated and experimental NMR chemical shifts of **6** (unscaled). Isomer 1: 1*R*,7*R*,8*S*,10*R*,12*R*-**6**; Isomer 2: 1*R*,7*R*,8*S*,10*S*,12*R*-**6**

Functional	Solvent?		Basis Set		Type of Data	
mPW1PW91	PCM		6-311G(d,p)		Unscaled Shifts	
	Isomer 1	Isomer 2	Isomer 3	Isomer 4	Isomer 5	Isomer 6
sDP4+ (H data)	84.50%	15.50%	-	-	-	-
sDP4+ (C data)	99.99%	0.01%	-	-	-	-
sDP4+ (all data)	100.00%	0.00%	-	-	-	-
uDP4+ (H data)	0.80%	99.20%	-	-	-	-
uDP4+ (C data)	99.95%	0.05%	-	-	-	-
uDP4+ (all data)	94.52%	5.48%	-	-	-	-
DP4+ (H data)	4.23%	95.77%	-	-	-	-
DP4+ (C data)	100.00%	0.00%	-	-	-	-
DP4+ (all data)	100.00%	0.00%	-	-	-	-

Functional		Solvent?		Basis Set		Type of Data	
mPW1PW91		PCM		6-311G(d,p)		Unscaled Shifts	
		DP4+	100.00%	0.00%	-	-	-
Nuclei	sp2?	Experimental	Isomer 1	Isomer 2	Isomer 3	Isomer 4	Isomer 5
C		44.6	44.4954	44.0338			
C		42.4	37.9731	38.0005			
C		25.1	21.8622	21.4497			
C	x	147.4	156.4149	155.2911			
C		35.4	33.4935	34.344			
C		29.7	30.3811	30.5338			
C		63	76.0138	76.5803			
C		75	77.0199	72.2694			
C		33.3	33.6197	33.8159			
C		54.7	59.1888	58.1856			
C		76.2	77.8232	80.6474			
C		50.2	46.5004	45.3765			
C		27.7	23.614	23.3259			
C		38.8	35.418	34.9465			
C		24.1	14.7326	14.499			
C	x	113.8	114.992	113.9682			
C		67	67.0767	62.417			
C		75.4	71.3471	72.1775			
C		29.6	24.3962	24.1539			
C		26	21.5274	21.3832			
H		1.25	1.3819	1.2792			
H		1.96	2.0432	2.0601			
H		1.63	1.9091	1.8248			
H		2.11	2.1012	2.0765			
H		2.28	2.3217	2.2561			
H		2.42	2.6501	2.4668			
H		1.9	2.0126	2.0459			
H		2.11	2.5913	2.8141			
H		4.04	4.5341	4.6118			
H		2.25	1.9405	1.806			
H		2.25	2.4485	2.4734			
H		2.89	2.5307	2.4959			
H		2.21	2.339	2.3474			
H		1.63	1.7374	1.7576			
H		1.9	1.847	1.8244			
H		1.76	1.5056	1.5045			
H		1.76	1.6255	1.6152			
H	x	0.86	1.13126601	1.129			
H	x	4.84	5.3612	5.3401			
H		5.03	5.5369	5.5154			
H		3.65	3.3435	3.608			
H		3.87	4.1124	3.9077			
H		1.21	1.14853272	1.16979934			
H		1.27	1.37696631	1.39223302			

Table S15 Energy analyses of 1*R*,7*R*,8*S*,10*R*-**8** (four conformers)

**NO.**	**3D conformers** B3LYP/6-31G(d,p)	**E (Hartree)**	**ΔE (KJ/mol)**	**Boltzmann distribution**
1*R*,7*R*,8*S*,10*R*-**8** (conformer 1)	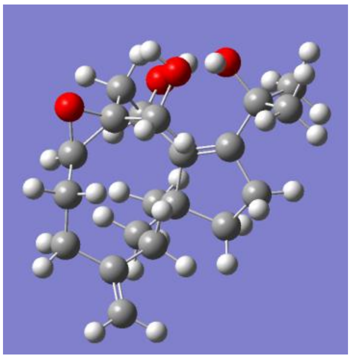	-1081.750344	0	96.27%
1*R*,7*R*,8*S*,10*R*-**8** (conformer 2)	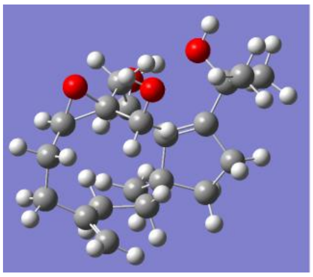	-1081.744634	14.9915984	0.23%
1*R*,7*R*,8*S*,10*R*-**8** (conformer 3)	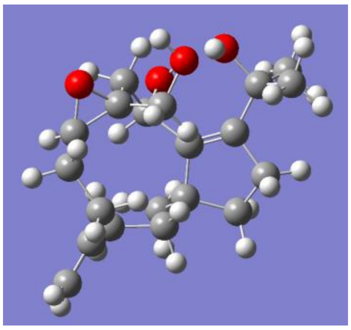	-1081.747207	8.236189876	3.47%
1*R*,7*R*,8*S*,10*R*-**8** (conformer 4)	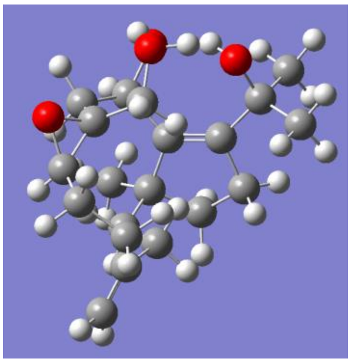	-1081.742677	20.12969964	0.03%

Table S16 Cartesian coordinates of the low-energy re-optimized conformers of 1*R*,7*R*,8*S*,10*R*-**8** calculated at B3LYP/6-31G(d,p) level of theory

1*R*,7*R*,8*S*,10*R*-**8** (conformer 1)							
Atomic	Standard Orientation (Å)			Atomic	Standard Orientation (Å)		
Type	X	Y	Z	Type	X	Y	Z
C	3.450372	1.381008	-0.146959	H	2.41802	1.379802	1.780163
C	4.023386	-0.047126	0.045493	H	-0.197979	-0.088978	2.501219
C	3.411057	-1.134861	-0.818721	H	0.148255	2.145641	2.356366
C	1.917152	-1.377861	-0.742748	H	-1.069566	2.408882	1.139014
C	1.415684	-1.825124	0.642038	H	-1.385868	-3.682879	0.179699
C	2.267729	1.710303	0.746881	H	0.037481	-3.528028	-0.835285
C	-0.118617	-2.019818	0.790801	H	-1.034455	-1.824807	-2.170189
C	-0.939176	-0.716773	0.627676	H	-2.501536	-2.538503	-1.529623
C	-0.840922	0.341665	1.729196	H	3.772089	-2.649374	-2.260955
C	-0.2424	1.750671	1.412955	H	5.255515	-1.693983	-1.712143
C	0.851472	1.925197	0.368385	H	-3.868365	1.013036	-2.557632
C	-0.726226	-2.952437	-0.301825	H	-2.108994	0.859258	-2.702856
C	-1.562002	-2.055717	-1.232669	H	-3.140289	-0.572091	-2.885868
C	-1.783765	-0.78494	-0.42435	H	-5.09906	0.140626	-0.571087
C	4.183295	-1.859631	-1.636486	H	-4.21668	-0.614777	0.778649
C	-2.944705	0.131501	-0.813456	H	-4.418159	-1.492361	-0.752043
C	-3.015087	0.364584	-2.334183	H	-2.216243	1.966656	-0.595436
C	-4.254395	-0.505214	-0.309248	H	0.127704	-2.098458	2.99171
O	-2.894049	1.409534	-0.157421	H	-1.436218	-2.689032	2.410288
C	-0.365601	-2.649773	2.183555	H	0.028491	-3.67355	2.201921
O	-2.092076	0.539672	2.3863	H	-2.64995	0.973656	1.709195
C	0.414156	1.945005	-1.086372	H	1.256472	2.215457	-1.731432
O	1.73997	3.041777	0.671356	H	0.029369	0.972972	-1.394495
O	-0.674374	2.85399	-1.301758	H	-0.394531	3.729229	-0.985322
H	3.222742	1.562004	-1.202239				
H	4.234179	2.101334	0.122575				
H	5.098403	-0.016409	-0.161254				
H	3.933288	-0.316583	1.108766				
H	1.649578	-2.131826	-1.491485				
H	1.389135	-0.463317	-1.030186				
H	1.743928	-1.104307	1.402886				
H	1.909019	-2.773037	0.899495				

1*R*,7*R*,8*S*,10*R*-**8** (conformer 2)							
Atomic	Standard Orientation (Å)			Atomic	Standard Orientation (Å)		
Type	X	Y	Z	Type	X	Y	Z
C	-3.88103	-1.092555	0.07022	H	-2.676699	-1.256856	1.897441
C	-4.253906	0.409359	0.13219	H	-0.252134	0.381767	2.096014
C	-3.261834	1.32315	-0.564345	H	-0.348656	-2.029765	2.167433
C	-2.055537	1.714282	0.26816	H	0.739214	-2.18553	0.791631
C	-0.805442	2.172428	-0.498844	H	2.364937	3.303546	0.07083
C	-2.627287	-1.487134	0.827521	H	1.243391	3.554338	-1.269316
C	0.540941	2.139684	0.289475	H	1.883271	1.414959	-2.268639
C	1.111982	0.704165	0.492858	H	3.399349	1.875064	-1.514072
C	0.544994	-0.183896	1.608154	H	-2.759563	2.370012	-2.347521
C	-0.053152	-1.567357	1.219263	H	-4.360047	1.451734	-2.370532
C	-1.255953	-1.64392	0.284804	H	3.725931	-2.229472	-1.856977
C	1.644097	2.799111	-0.584212	H	1.974781	-1.918465	-1.811126
C	2.351381	1.643977	-1.298923	H	3.07634	-0.780651	-2.629907
C	2.16094	0.478961	-0.333797	H	5.275899	-1.038784	-0.264558
C	-3.465752	1.735371	-1.820195	H	4.716029	0.39572	0.621277
C	3.143325	-0.681284	-0.443878	H	4.909996	0.443586	-1.146984
C	2.960373	-1.446077	-1.768022	H	3.501829	-2.354337	0.53221
C	4.596851	-0.177419	-0.303526	H	-0.375485	2.513713	2.271791
O	2.912205	-1.593244	0.652375	H	1.350997	2.864125	2.181331
C	0.411186	2.906832	1.62087	H	0.176473	3.961076	1.423721
O	1.501092	-0.373273	2.652298	H	2.237764	-0.856622	2.232785
C	-0.970892	-1.544199	-1.205812	H	-1.893771	-1.391648	-1.777285
O	-2.105688	-2.799027	0.567646	H	-0.301702	-0.706558	-1.410885
O	-0.289177	-2.713327	-1.653579	H	-0.818519	-3.464567	-1.335867
H	-3.802737	-1.406304	-0.974771				
H	-4.707707	-1.67205	0.504698				
H	-5.244181	0.523605	-0.323328				
H	-4.358713	0.709758	1.185317				
H	-1.806787	0.866643	0.908968				
H	-2.370282	2.505467	0.965568				
H	-0.965042	3.202258	-0.845787				
H	-0.693351	1.564335	-1.405143				

1*R*,7*R*,8*S*,10*R*-**8** (conformer 3)							
Atomic Type	Standard Orientation (Å) X	Y	Z	Atomic Type	Standard Orientation (Å) X	Y	Z
C	-3.490319	-1.571645	-0.465854	H	-2.728224	-1.312479	1.587622
C	-3.468733	-0.234556	-1.248775	H	-0.099996	0.577199	2.239713
C	-3.583169	1.025171	-0.40957	H	-0.508253	-1.654419	2.467893
C	-2.360237	1.486774	0.362563	H	0.824732	-2.15039	1.467266
C	-1.201196	1.974166	-0.534891	H	1.79261	3.569206	-0.268226
C	-2.435047	-1.72199	0.618482	H	0.605183	3.501138	-1.569221
C	0.185351	2.164555	0.153865	H	1.598506	1.450419	-2.434551
C	0.951003	0.832988	0.421008	H	3.024805	2.151918	-1.693458
C	0.615849	-0.0092	1.659805	H	-4.835304	2.609925	0.242793
C	-0.005347	-1.442489	1.518642	H	-5.609575	1.401585	-0.921967
C	-0.974121	-1.816444	0.40367	H	4.272604	-1.526396	-1.84868
C	1.144451	2.8912	-0.836221	H	2.586656	-1.304737	-2.349338
C	2.009637	1.799011	-1.474858	H	3.750082	0.022613	-2.53922
C	1.980836	0.681237	-0.44369	H	5.184927	-0.41476	0.195909
C	-4.730754	1.711285	-0.360729	H	4.132665	0.595076	1.219028
C	3.121154	-0.336457	-0.461296	H	4.6826	1.198682	-0.357519
C	3.448938	-0.806499	-1.891921	H	2.181674	-2.038991	-0.131155
C	4.36106	0.306669	0.188845	H	-0.656922	2.601689	2.160726
O	2.84994	-1.496779	0.340742	H	1.008572	3.138369	1.925078
C	0.039183	3.021899	1.42876	H	-0.330901	4.021238	1.164933
O	1.743015	-0.133391	2.527645	H	2.356418	-0.72015	2.040401
C	-0.403956	-1.927746	-0.997699	H	-1.172902	-2.266968	-1.699464
O	-1.772369	-2.990475	0.744406	H	-0.009379	-0.969408	-1.337024
O	0.71153	-2.831362	-1.031764	H	0.397474	-3.683805	-0.685251
H	-3.376417	-2.39689	-1.177536				
H	-4.475659	-1.695999	0.000165				
H	-2.548723	-0.19805	-1.846571				
H	-4.296789	-0.261622	-1.966406				
H	-2.00395	0.675501	1.003798				
H	-2.657751	2.298349	1.035149				
H	-1.504247	2.936788	-0.967993				
H	-1.068947	1.293861	-1.385947				

1*R*,7*R*,8*S*,10*R*-**8** (conformer 4)							
Atomic	Standard Orientation (Å)			Atomic	Standard Orientation (Å)		
Type	X	Y	Z	Type	X	Y	Z
C	-3.514045	-1.117597	-0.901018	H	-3.039236	-0.44992	1.12501
C	-3.18269	0.08072	-1.831124	H	1.034845	-0.208277	2.737555
C	-2.893689	1.394776	-1.119948	H	-1.214446	-0.544068	2.378215
C	-1.432363	1.674506	-0.780135	H	-0.467746	-2.113092	2.483692
C	-1.137048	1.928	0.710837	H	1.988424	3.31421	0.986894
C	-2.716032	-1.188536	0.388563	H	0.594453	3.770927	0.008073
C	0.363079	1.880684	1.13677	H	1.23404	2.190775	-1.730011
C	1.082375	0.540666	0.778802	H	2.824422	2.527508	-1.076054
C	0.85969	-0.631432	1.741365	H	-3.678572	3.204861	-0.328488
C	-0.56846	-1.237099	1.830744	H	-4.906767	2.063879	-1.102799
C	-1.335313	-1.700927	0.580804	H	3.337171	-0.824277	-3.058749
C	1.196983	2.917352	0.33944	H	1.640908	-0.354134	-2.843856
C	1.834904	2.136786	-0.812151	H	2.902595	0.892749	-2.942134
C	1.895191	0.700156	-0.29663	H	5.014962	-0.487795	-1.212464
C	-3.8712	2.262021	-0.835278	H	4.48259	0.01849	0.405045
C	2.871251	-0.218293	-1.046144	H	4.560423	1.203346	-0.912068
C	2.671942	-0.10776	-2.56698	H	2.714342	-1.672059	0.225892
C	4.32227	0.162956	-0.669055	H	-0.226851	1.580531	3.249209
O	2.71422	-1.611389	-0.758239	H	1.444306	2.139267	3.028282
C	0.421994	2.221548	2.641307	H	0.090302	3.255182	2.798361
O	1.812006	-1.707699	1.660246	H	1.331801	-2.45345	1.231636
C	-0.565529	-2.220312	-0.618843	H	-1.264527	-2.73265	-1.288382
O	-2.491575	-2.482894	0.960763	H	-0.13229	-1.367282	-1.154568
O	0.479043	-3.125471	-0.247157	H	1.272005	-2.824643	-0.741994
H	-3.40334	-2.054337	-1.456652				
H	-4.570669	-1.054448	-0.609671				
H	-2.315933	-0.181564	-2.451155				
H	-4.026464	0.208774	-2.518951				
H	-1.098065	2.540443	-1.367016				
H	-0.823698	0.831681	-1.119731				
H	-1.704098	1.212972	1.312158				
H	-1.524308	2.916729	0.991869				

Table S17 Energy analyses of 1*S*,7*S*,8*R*,10*S*-**8** (four conformers)

**NO.**	**3D conformers** B3LYP/6-31G(d,p)	**E (Hartree)**	**ΔE (KJ/mol)**	**Boltzmann distribution**
1*S*,7*S*,8*R*,10*S*-**8** (conformer 1)	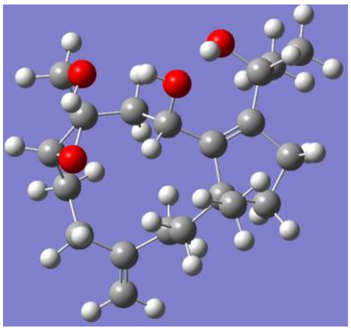	-1081.756891	0	88.45%
1*S*,7*S*,8*R*,10*S*-**8** (conformer 2)	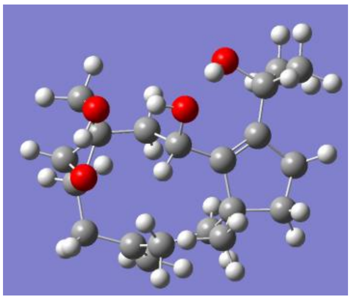	-1081.753153	9.814114681	1.69%
1*S*,7*S*,8*R*,10*S*-**8** (conformer 3)	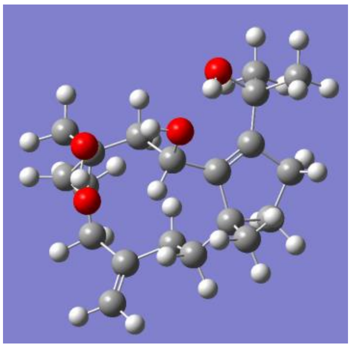	-1081.754016	7.548309178	4.21%
1*S*,7*S*,8*R*,10*S*-**8** (conformer 4)	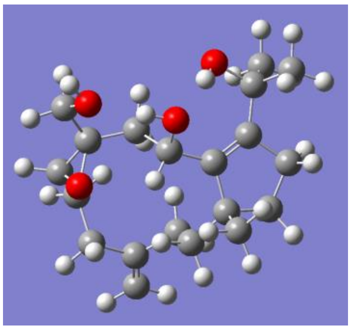	-1081.754294	6.8184205	5.65%

Table S18 Cartesian coordinates of the low-energy re-optimized conformers of 1*S*,7*S*,8*R*,10*S*-**8** calculated at B3LYP/6-31G(d,p) level of theory

1*S*,7*S*,8*R*,10*S*-**8** (conformer 1)							
Atomic	Standard Orientation (Å)			Atomic	Standard Orientation (Å)		
Type	X	Y	Z	Type	X	Y	Z
C	-3.314475	0.22175	-1.25856	H	-3.818856	2.332618	-0.773084
C	-3.598069	-0.9237	-0.239825	H	-0.430773	0.548505	1.443159
C	-2.921407	-2.243562	-0.566773	H	0.265565	1.80833	-1.214293
C	-1.404906	-2.319463	-0.471353	H	-0.723218	0.37475	-1.146043
C	-0.851945	-2.104919	0.957411	H	1.802852	-3.682358	1.055156
C	-3.041391	1.582819	-0.610601	H	1.065672	-3.236294	-0.483819
C	0.663073	-1.787231	1.097879	H	3.248772	-2.456457	-0.96942
C	1.135773	-0.467643	0.441245	H	3.61261	-2.100274	0.709931
C	0.324209	0.790061	0.685988	H	-3.175788	-4.271779	-1.140013
C	-0.436847	1.259928	-0.579401	H	-4.727413	-3.279924	-0.982083
C	-1.678726	2.095776	-0.298682	H	5.169809	1.29657	-0.599216
C	1.569055	-2.834259	0.401586	H	4.34001	0.892913	0.919089
C	2.815471	-2.053696	-0.046863	H	5.099555	-0.391348	-0.044681
C	2.319618	-0.619684	-0.189724	H	4.079585	0.764499	-2.828088
C	-3.64246	-3.315763	-0.91396	H	2.564566	-0.155947	-2.885726
C	3.216717	0.404913	-0.885758	H	4.074705	-0.955945	-2.388374
C	4.541801	0.54998	-0.102066	H	2.285156	1.946905	-0.119549
C	3.502568	-0.025157	-2.336259	H	0.457843	-0.887573	3.095826
O	2.616877	1.691092	-1.009187	H	0.686897	-2.643847	3.117895
C	0.985836	-1.717523	2.611216	H	2.057908	-1.566602	2.778316
O	1.160745	1.830432	1.229416	H	0.59695	2.631017	1.317891
C	-1.472199	3.588375	-0.104704	H	-0.799867	4.003977	-0.859462
O	-2.554412	1.587548	0.744441	H	-2.430529	4.121806	-0.155414
O	-0.847765	3.832593	1.164676	H	-1.459626	3.469278	1.829147
H	-4.171674	0.334435	-1.930676				
H	-2.474911	-0.053884	-1.904544				
H	-3.273017	-0.583926	0.748218				
H	-4.67897	-1.084746	-0.168994				
H	-0.951508	-1.583501	-1.148029				
H	-1.085767	-3.297466	-0.847329				
H	-1.065444	-3.009059	1.543759				
H	-1.414827	-1.304016	1.450896				

1*S*,7*S*,8*R*,10*S*-**8** (conformer 2)							
Atomic	Standard Orientation (Å)			Atomic	Standard Orientation (Å)		
Type	X	Y	Z	Type	X	Y	Z
C	-3.395991	0.091977	-1.476613	H	-3.93233	2.111179	-0.76574
C	-3.833185	-1.181798	-0.680456	H	-0.358688	0.553589	1.562739
C	-2.728243	-2.214302	-0.520408	H	0.088701	1.627995	-1.237166
C	-1.721227	-1.961749	0.590151	H	-0.793748	0.140222	-0.983933
C	-0.28671	-2.474015	0.333904	H	2.785408	-2.466594	1.859793
C	-3.149467	1.356986	-0.656219	H	2.01688	-3.630299	0.779288
C	0.85805	-1.77129	1.134585	H	2.711905	-2.355921	-1.175754
C	1.237831	-0.394769	0.528287	H	4.015416	-1.913624	-0.087246
C	0.325559	0.797811	0.742867	H	-1.881973	-4.019013	-1.262264
C	-0.539111	1.093637	-0.517331	H	-3.422366	-3.445205	-2.101125
C	-1.817091	1.866983	-0.23688	H	4.98554	1.751396	-0.68378
C	2.18579	-2.560612	0.946187	H	4.136097	1.390048	0.836531
C	2.926185	-1.875091	-0.209655	H	5.070699	0.120928	0.020012
C	2.373698	-0.457029	-0.199095	H	4.04205	0.956749	-2.883108
C	-2.671756	-3.276203	-1.332246	H	2.63195	-0.117086	-2.927491
C	3.148663	0.629298	-0.949152	H	4.194186	-0.724856	-2.329127
C	4.41883	0.986644	-0.141805	H	2.077704	2.125424	-0.294104
C	3.530223	0.144147	-2.357997	H	-0.430732	-1.178695	2.835943
O	2.403655	1.8242	-1.171067	H	0.40789	-2.716852	3.043337
C	0.508751	-1.703117	2.634367	H	1.302452	-1.192931	3.191722
O	1.094304	1.942218	1.156291	H	0.446632	2.649484	1.371839
C	-1.688075	3.351415	0.069963	H	-0.9922	3.840137	-0.616941
O	-2.720991	1.235107	0.71484	H	-2.664617	3.846114	-0.01232
O	-1.157789	3.556016	1.387183	H	-1.755476	3.077902	1.988804
H	-4.187286	0.341808	-2.192542				
H	-2.517518	-0.152283	-2.082684				
H	-4.2126	-0.87929	0.30335				
H	-4.669724	-1.644644	-1.214429				
H	-2.112202	-2.421817	1.510435				
H	-1.723091	-0.892731	0.804202				
H	-0.057917	-2.387828	-0.735848				
H	-0.250817	-3.547168	0.564899				

1*S*,7*S*,8*R*,10*S*-**8** (conformer 3)							
Atomic	Standard Orientation (Å)			Atomic	Standard Orientation (Å)		
Type	X	Y	Z	Type	X	Y	Z
C	2.936646	0.224495	1.946072	H	3.89248	1.892359	0.866572
C	3.5474	-1.154498	1.595589	H	0.474952	0.380992	-1.41157
C	2.905305	-1.928238	0.453999	H	-0.246724	2.216793	0.841735
C	1.396325	-2.140685	0.496699	H	0.392738	0.664874	1.290671
C	0.715638	-2.194835	-0.887556	H	-1.94802	-3.692051	-0.617608
C	2.990207	1.274638	0.847087	H	-1.139721	-3.056988	0.819033
C	-0.791823	-1.835334	-0.950336	H	-3.312683	-2.189926	1.270219
C	-1.185392	-0.441932	-0.402383	H	-3.715406	-2.031349	-0.430561
C	-0.318637	0.750138	-0.754358	H	3.262125	-3.043699	-1.321312
C	0.377827	1.394885	0.481874	H	4.749621	-2.30525	-0.518481
C	1.793472	1.871475	0.189834	H	-5.178345	1.499989	0.528274
C	-1.678676	-2.769144	-0.091135	H	-4.459796	0.825563	-0.949038
C	-2.896733	-1.912535	0.294363	H	-5.154213	-0.259638	0.27368
C	-2.362486	-0.48542	0.256213	H	-3.972696	1.303313	2.745205
C	3.672338	-2.452486	-0.507596	H	-2.431456	0.432211	2.854077
C	-3.214683	0.643036	0.834685	H	-3.944738	-0.465785	2.588207
C	-4.58997	0.668073	0.127849	H	-2.290728	2.026298	-0.217274
C	-3.403588	0.455024	2.351089	H	-0.722136	-1.119696	-3.026855
O	-2.615237	1.928891	0.704744	H	-0.95887	-2.870483	-2.879335
C	-1.219358	-1.899777	-2.438203	H	-2.299381	-1.753301	-2.546966
O	-1.079847	1.719592	-1.503643	H	-0.445562	2.435565	-1.722725
C	1.919612	3.240324	-0.452491	H	1.385255	3.998546	0.12689
O	2.623373	0.894474	-0.489437	H	2.974884	3.534826	-0.524046
O	1.320537	3.250477	-1.757405	H	1.824582	2.611355	-2.289902
H	3.486588	0.628677	2.805601				
H	1.910415	0.083496	2.29546				
H	4.617346	-1.03825	1.384322				
H	3.48246	-1.757755	2.514809				
H	0.931612	-1.34752	1.088307				
H	1.187965	-3.064648	1.056282				
H	0.830969	-3.200581	-1.313646				
H	1.265328	-1.528632	-1.561879				

1*S*,7*S*,8*R*,10*S*-**8** (conformer 4)							
Atomic	Standard Orientation (Å)			Atomic	Standard Orientation (Å)		
Type	X	Y	Z	Type	X	Y	Z
C	-3.30308	0.331883	-1.18387	H	-3.779431	2.430847	-0.637498
C	-3.617829	-0.829643	-0.195306	H	-0.470868	0.45883	1.463706
C	-2.998457	-2.161098	-0.579652	H	0.330545	1.89636	-1.066982
C	-1.48436	-2.285114	-0.516197	H	-0.692062	0.487395	-1.147012
C	-0.906104	-2.169112	0.913642	H	1.71682	-3.789173	0.896643
C	-3.008643	1.668674	-0.498423	H	0.969236	-3.257972	-0.610444
C	0.613632	-1.877599	1.041687	H	3.164996	-2.4948	-1.084261
C	1.095056	-0.538568	0.432797	H	3.54878	-2.223405	0.606473
C	0.310434	0.723176	0.743086	H	-3.33113	-4.158017	-1.218502
C	-0.402702	1.315997	-0.499765	H	-4.845756	-3.122792	-0.991754
C	-1.636762	2.158712	-0.18353	H	5.191396	1.131475	-0.612359
C	1.49112	-2.907354	0.286205	H	4.430788	0.61603	0.907566
C	2.74603	-2.128565	-0.139597	H	5.075291	-0.59806	-0.217514
C	2.273545	-0.681386	-0.211249	H	3.980989	0.824459	-2.817782
C	-3.761375	-3.196144	-0.948949	H	2.402733	0.015677	-2.868336
C	3.187237	0.354547	-0.86682	H	3.874008	-0.923409	-2.521742
C	4.558649	0.365763	-0.151838	H	2.323033	1.844631	0.066703
C	3.373133	0.035974	-2.361922	H	0.461628	-1.051036	3.074338
O	2.656727	1.676727	-0.844071	H	0.651263	-2.812326	3.026479
C	0.962696	-1.873699	2.550724	H	2.040456	-1.752693	2.705358
O	1.171055	1.689891	1.379712	H	0.656974	2.523964	1.434136
C	-1.425158	3.654486	-0.088819	H	-1.058895	4.030078	-1.054983
O	-2.507309	1.64217	0.845723	H	-2.37658	4.149263	0.145238
O	-0.470231	3.918823	0.948924	H	-0.104791	4.806666	0.822033
H	-4.151366	0.478022	-1.861107				
H	-2.463369	0.052529	-1.828096				
H	-3.259092	-0.533239	0.795332				
H	-4.703005	-0.948593	-0.106855				
H	-1.021287	-1.519756	-1.152639				
H	-1.196341	-3.246564	-0.954974				
H	-1.119292	-3.106	1.446195				
H	-1.449986	-1.394856	1.467124				

Table S19 Experimental and calculated ^{1}H NMR data for compound **8**

No.	**8**, exptl. δ_H [a]	1*R*,7*R*,8*S*,10*R*-**8**, calcd. δ_H [b]	1*S*,7*S*,8*R*,10*S*-**8**, calcd. δ_H [b]
2	1.32	1.17	1.26
	1.74	1.68	1.73
3	1.79	1.79	2.03
	2.04	2.22	2.16
5	2.27	2.40	2.27
	2.46	2.45	2.66
6	1.49	1.18	1.44
	1.96	2.13	2.04
7	3.13	3.04	3.34
9	1.96	1.63	1.83
	2.58	2.83	3.01
10	4.37	4.18	4.44
13	2.2	2.29	2.32
	2.35	2.50	2.54
14	1.43	1.71	1.44
	1.74	1.98	1.89
15	1.05	1.22	1.11
16	4.67	5.02	5.00
	4.71	5.06	5.08
17	3.32	3.52	3.53
	3.98	4.52	4.00
19	1.35	1.42	1.35
20	1.38	1.41	1.38

[a] Recorded in $CDCl_3$ at 600 MHz.

[b] Calculated in $CDCl_3$

Table S20 Experimental and calculated ^{13}C NMR data for compound **8**

No.	**8**, exptl. δ_C [a]	1*R*,7*R*,8*S*,10*R*-**8**, calcd. δ_C [b]	1*S*,7*S*,8*R*,10*S*-**8**, calcd. δ_C [b]
1	53.1	52.88	53.00
2	35.7	35.70	32.59
3	29.9	29.84	28.88
4	150.3	153.27	154.83
5	28.7	29.02	24.95
6	22.3	24.65	20.58
7	60.2	61.36	62.17
8	63.8	59.06	62.89
9	37.8	39.73	36.33
10	65	60.95	63.32
11	141.2	145.47	143.22
12	144.7	150.72	152.53
13	32.8	32.66	31.03
14	32.7	31.42	29.44
15	26.8	26.93	21.18
16	111.1	110.33	111.98
17	68.6	61.37	68.24
18	72.3	71.84	70.58
19	31	22.68	26.44
20	29.7	24.56	24.37

[a] Recorded in $CDCl_3$ at 600 MHz.

[b] Calculated in $CDCl_3$

Table S21 DP4+ analyses of calculated and experimental NMR chemical shifts of **8** (unscaled). Isomer 1: 1*R*-7*R*-8*S*-10*R*-**8**; Isomer 2: 1*S*-7*S*-8*R*-10*S*-**8**

Functional	Solvent?	Basis Set	Type of Data
mPW1PW91	PCM	6-311G(d,p)	Unscaled Shifts

	Isomer 1	Isomer 2	Isomer 3	Isomer 4	Isomer 5	Isomer 6
sDP4+ (H data)	0.00%	100.00%	-	-	-	-
sDP4+ (C data)	0.00%	100.00%	-	-	-	-
sDP4+ (all data)	0.00%	100.00%	-	-	-	-
uDP4+ (H data)	0.04%	99.96%	-	-	-	-
uDP4+ (C data)	99.97%	0.03%	-	-	-	-
uDP4+ (all data)	62.82%	37.18%	-	-	-	-
DP4+ (H data)	0.00%	100.00%	-	-	-	-
DP4+ (C data)	0.00%	100.00%	-	-	-	-
DP4+ (all data)	0.00%	100.00%	-	-	-	-

Functional	Solvent?	Basis Set	Type of Data
mPW1PW91	PCM	6-311G(d,p)	Unscaled Shifts

		DP4+	0.00%	100.00%	-	-	-
**Nuclei**	**sp2?**	**Experimental**	**Isomer 1**	**Isomer 2**	**Isomer 3**	**Isomer 4**	**Isomer 5**
C		53.1	52.8765	52.998			
C		35.7	35.7018	32.5932			
C		29.9	29.8413	28.8842			
C	x	150.3	153.2658	154.8336			
C		28.7	29.0241	24.9488			
C		22.3	24.6492	20.5768			
C		60.2	61.3587	62.1674			
C		63.8	59.055	62.887			
C		37.8	39.7287	36.3324			
C		65	60.9468	63.3236			
C	x	141.2	145.4745	143.2214			
C	x	144.7	150.717	152.5332			
C		32.8	32.664	31.0284			
C		32.7	31.419	29.4386			
C		26.8	26.9277	21.1754			
C	x	111.1	110.3292	111.9846			
C		68.6	61.3713	68.2414			
C		72.3	71.835	70.5804			
C		31	22.6776	26.4434			
C		29.7	24.5574	24.368			
H		1.32	1.1742	1.2562			
H		1.74	1.6818	1.7252			
H		1.79	1.7907	2.0314			
H		2.04	2.2218	2.1616			
H		2.27	2.3967	2.2684			
H		2.46	2.4456	2.6622			
H		1.49	1.1787	1.4376			
H		1.96	2.1279	2.0406			
H		3.13	3.0423	3.3376			
H		1.96	1.6332	1.833			
H		2.58	2.8323	3.0124			
H		4.37	4.1838	4.4444			
H		2.2	2.2929	2.3236			
H		2.35	2.4999	2.5426			
H		1.43	1.7133	1.4384			
H		1.74	1.9767	1.8928			
H		1.05	1.2177	1.11466632			
H	x	4.67	5.0229	5.0036			
H	x	4.71	5.0631	5.0822			
H		3.32	3.5232	3.5272			
H		3.98	4.515	3.995			
H		1.35	1.4159	1.34653326			
H		1.38	1.412299	1.38059994			

Table S22 Cytotoxicity of active compounds against Human Oral Cancer (Ca9-22)

Compound/tumor cell	Ca9-22 (μM)	Compound/tumor cell	Ca9-22 (μM)
**1**	212.64 ± 2.64	**11**	238.51 ± 9.24
**3**	239.09 ± 2.17	**13**	112.45 ± 1.4
**4**	231.02± 11.72	**15**	24.87 ± 3.0
**5**	87.39 ± 4.42	**16**	166.7 ± 2.97
**6**	31.51 ± 16.03	**17**	156.99 ± 20.47
**7**	15.65 ± 0.37	**19**	190.26 ± 13.59
**8**	118.72 ± 4.14	**21**	72.23 ± 12.74
**10**	194.62 ± 4.97	**22**	43.62 ± 5.79

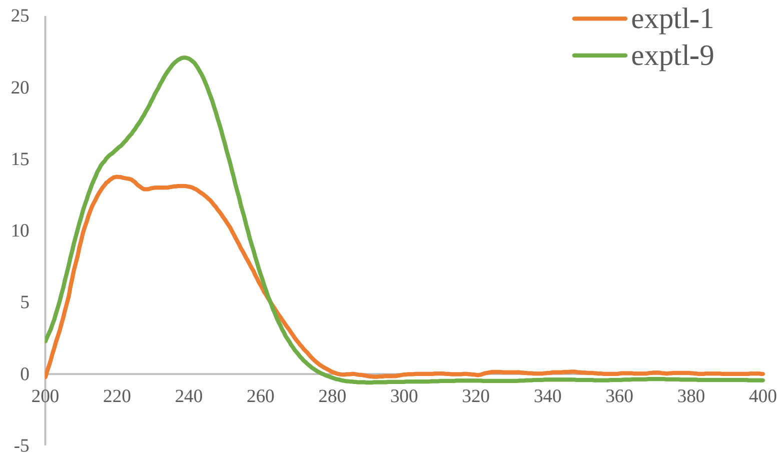

Figure S1 Experimental ECD spectra of **1** and **9**

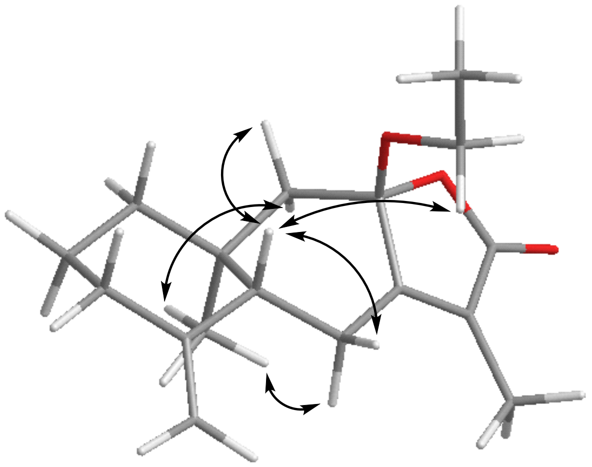

Figure S2 NOESY (double arrow) correlations of **2**

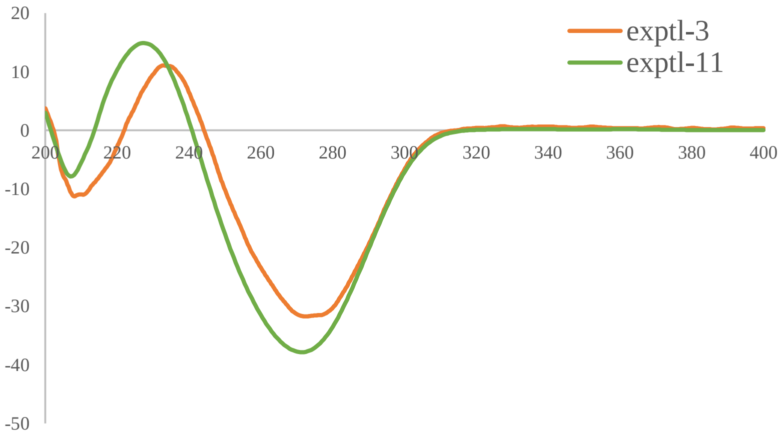

Figure S3 Experimental ECD spectra of **3** and **11**

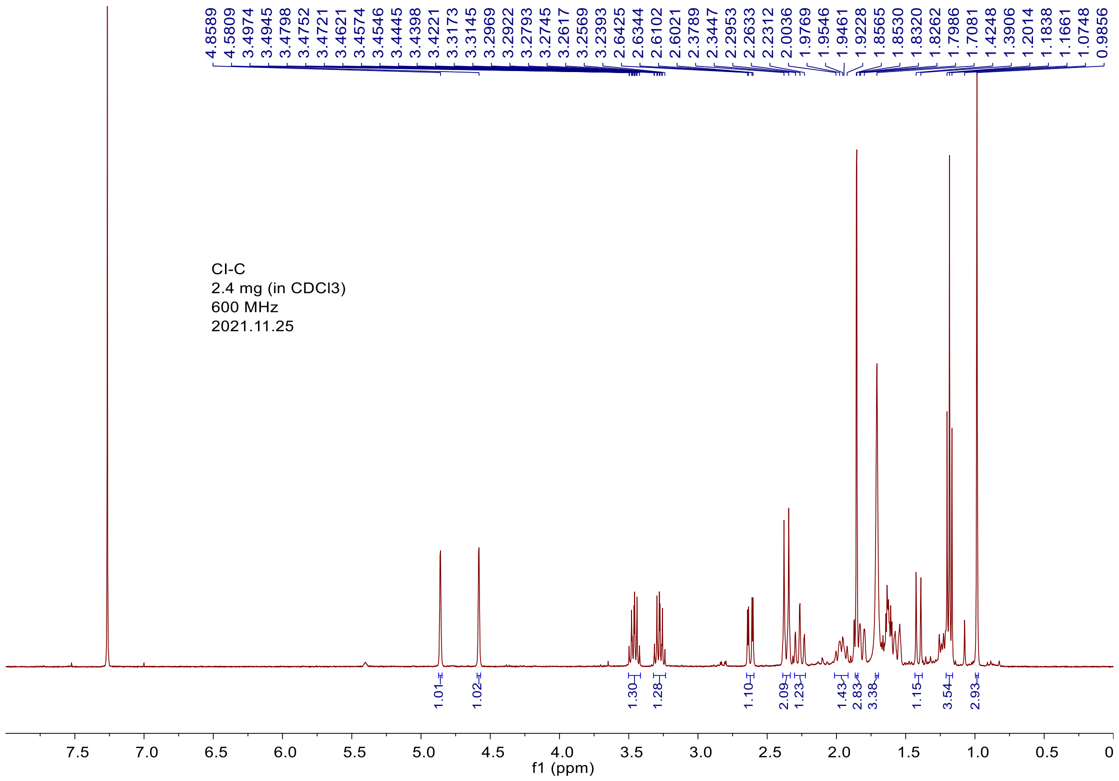

Figure S4 ^{1}H NMR spectrum of **1** (600 MHz, $CDCl_3$)

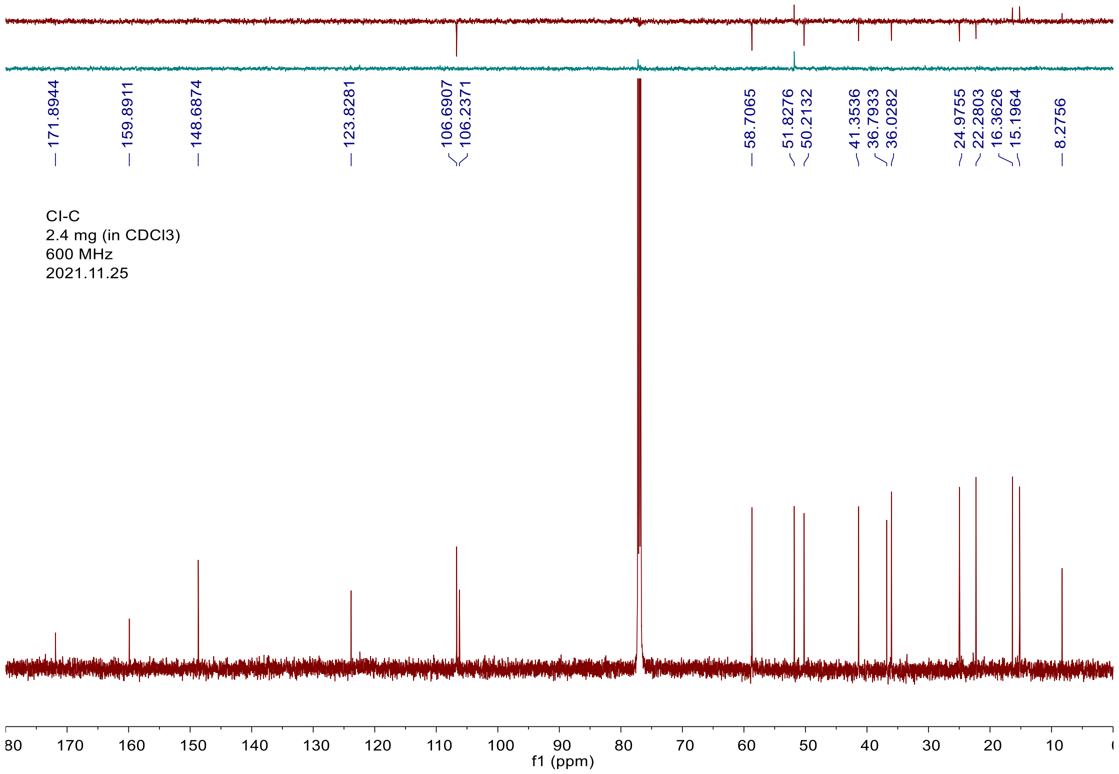

Figure S5 ^{13}C NMR spectrum of **1** (125 MHz, $CDCl_3$)

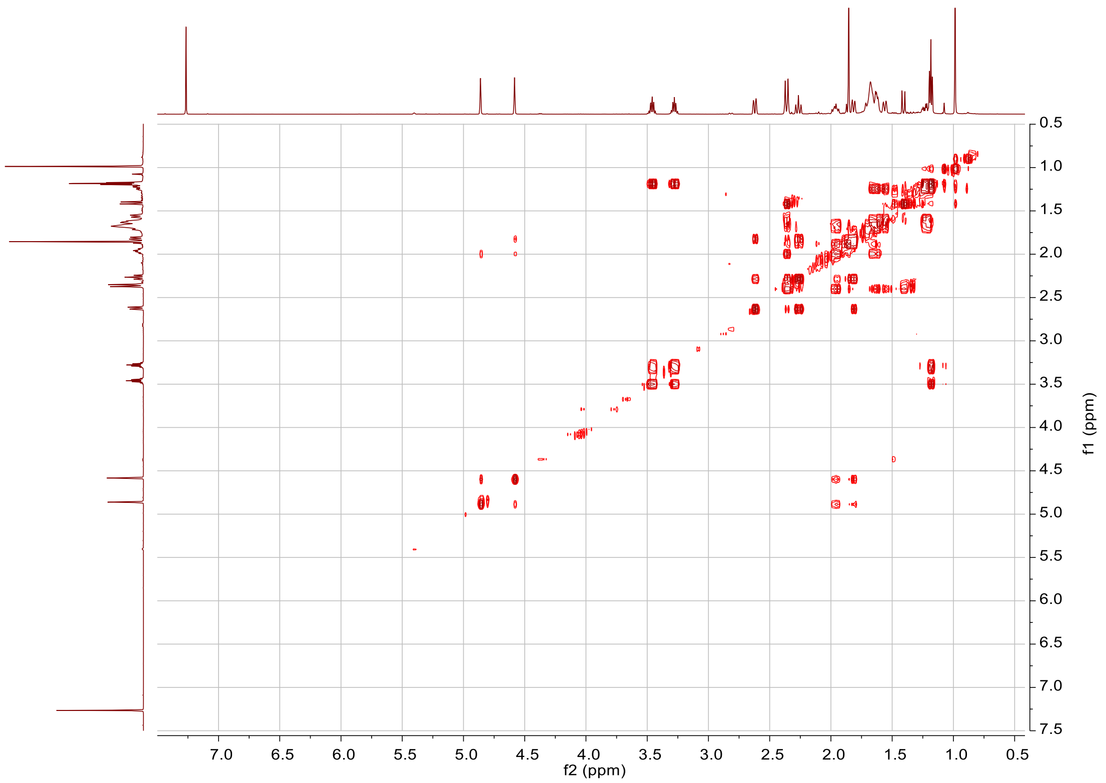

Figure S6 COSY spectrum of **1**

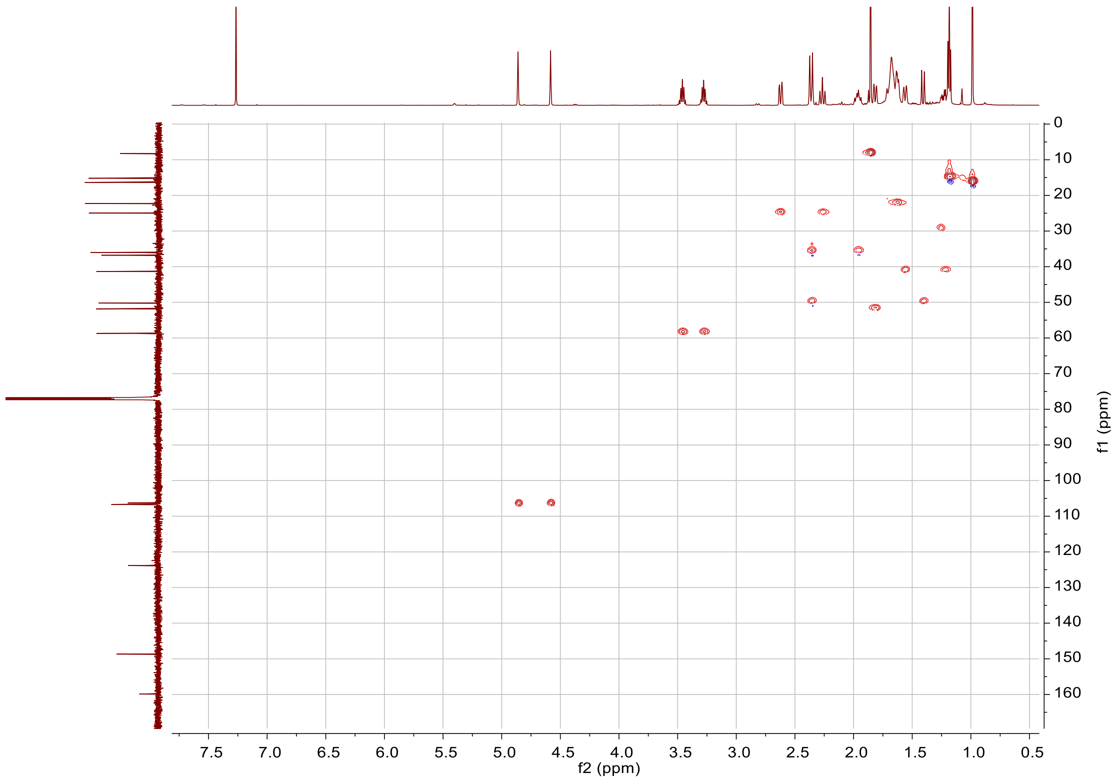

Figure S7 HSQC spectrum of **1**

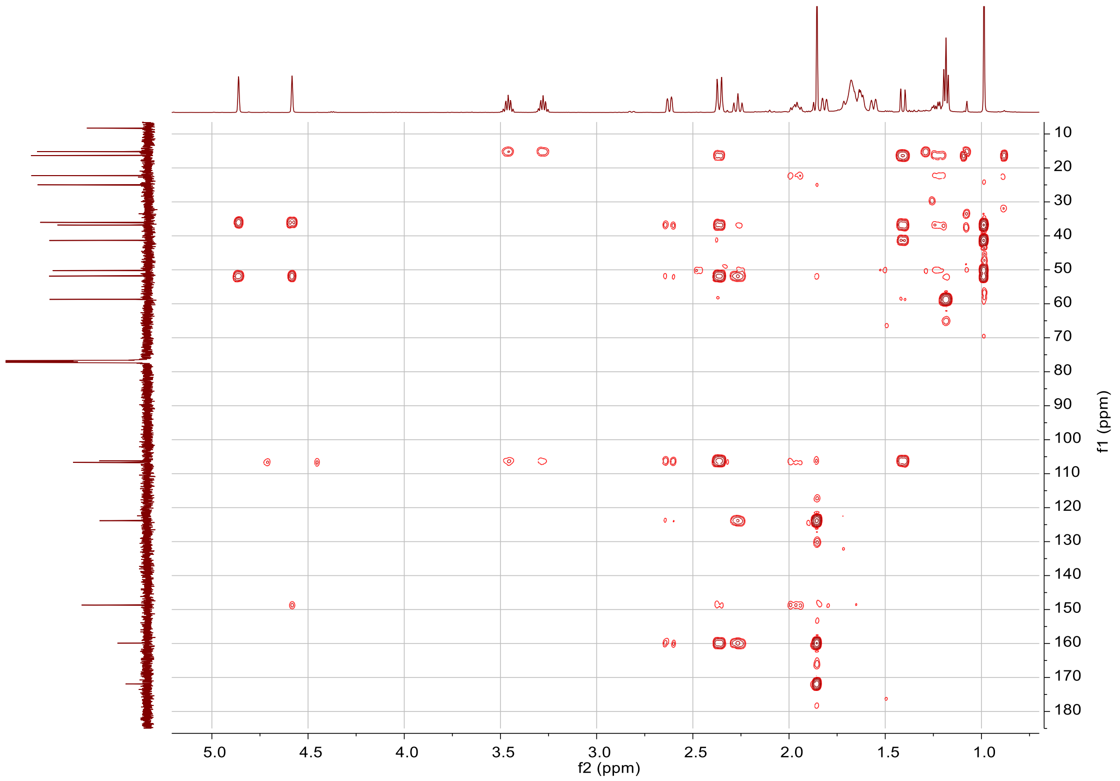

Figure S8 HMBC spectrum of **1**

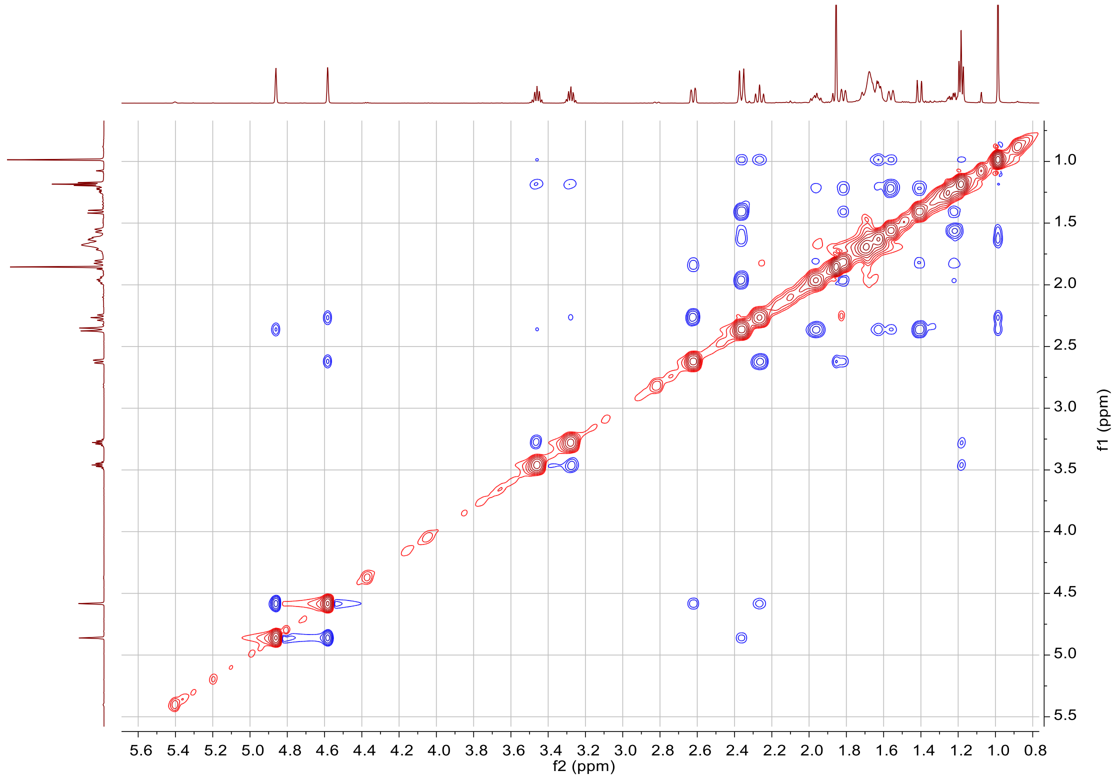

Figure S9 NOESY spectrum of **1**

Mass Spectrum SmartFormula Report

Analysis Info

Analysis Name	D:\QTOF\CICR.d	12/23/2021 1:03:53 PM
Method	_tune_low_pos(50-1000)_20170502_Liao lab.m	Operator: YU HSIAO-CHING
Sample Name	CI-C	Instrument: BRUKER micrOTOF-Q
Comment	ESI Positive	

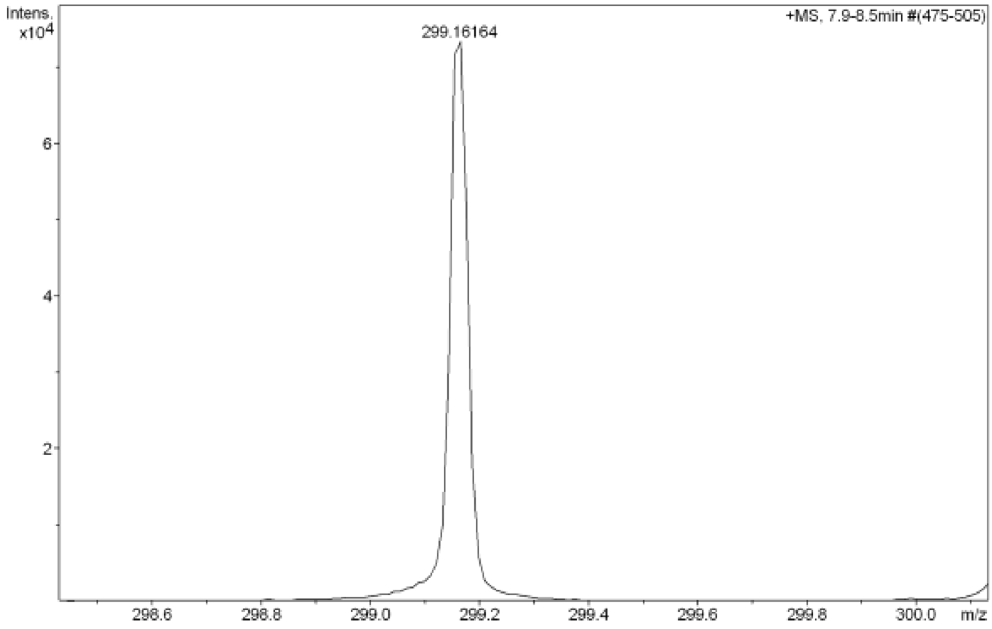

Meas. m/z	#	Formula	Score	m/z	err [mDa]	err [ppm]	mSigma	rdb	e⁻ Conf	N-Rule
299.16164	1	C 17 H 24 Na O 3	100.00	299.16177	0.12	0.41	39.7	5.5	even	ok

Figure S10 HRESIMS spectrum of **1**

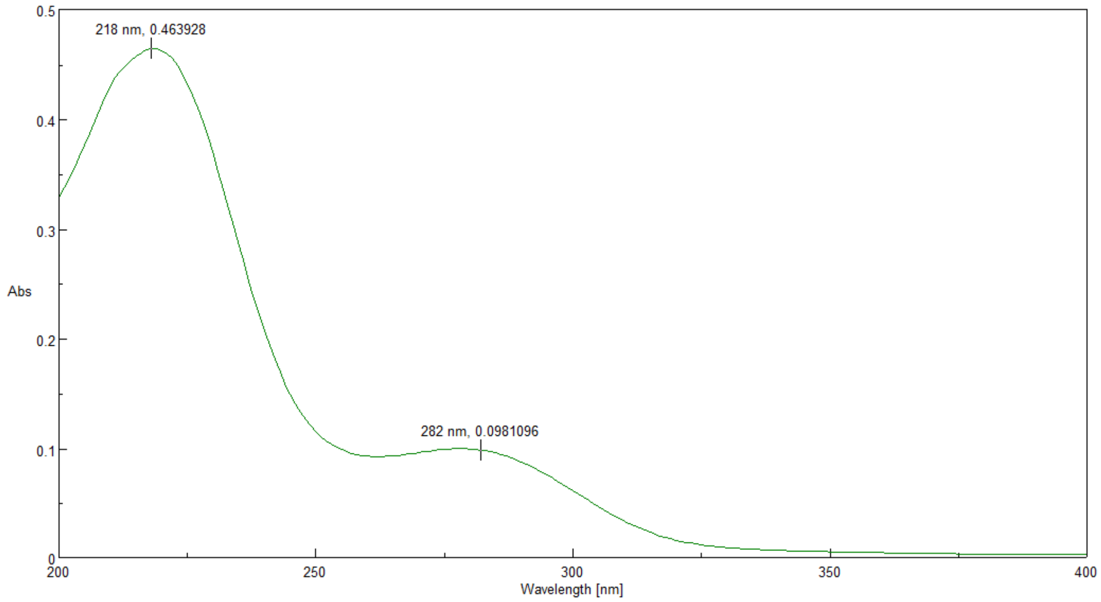

Figure S11 UV spectrum of **1**

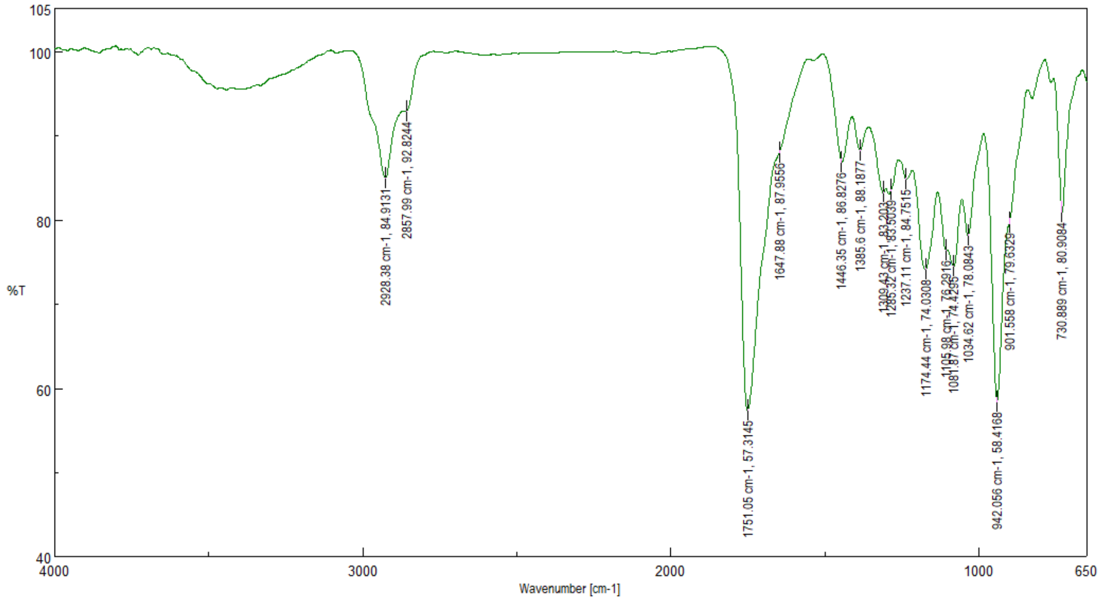

Figure S12 IR spectrum of **1**

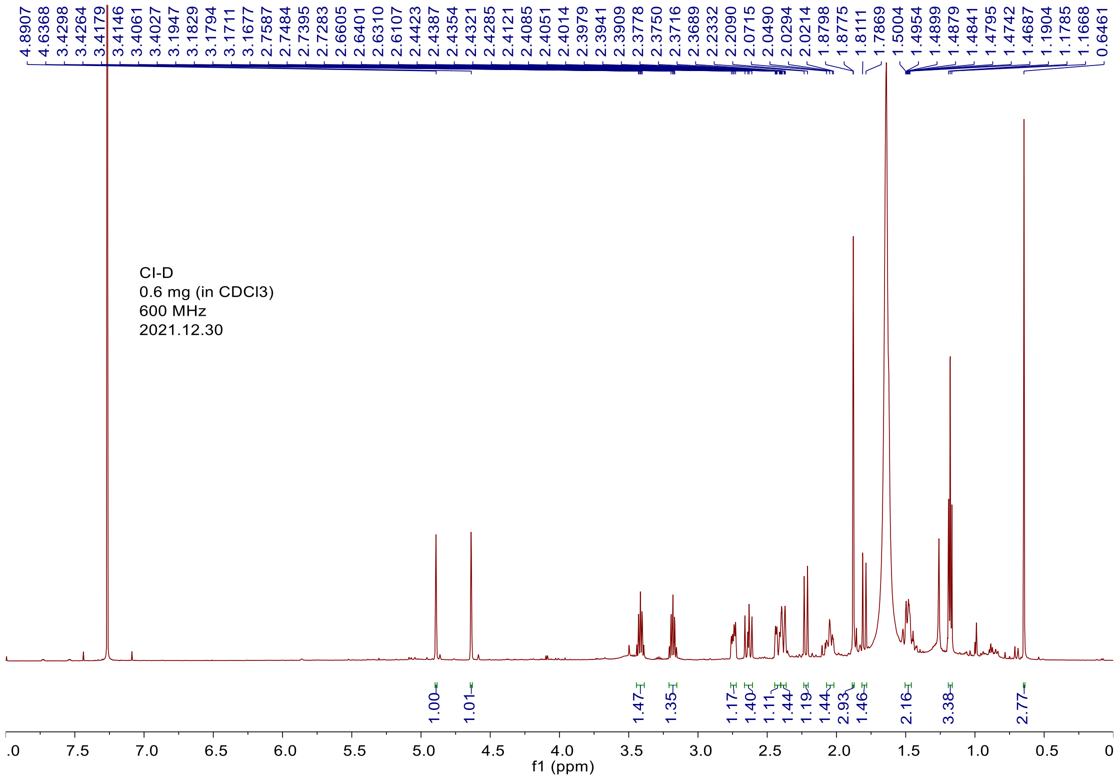

Figure S13 ^{1}H NMR spectrum of **2** (600 MHz, $CDCl_3$)

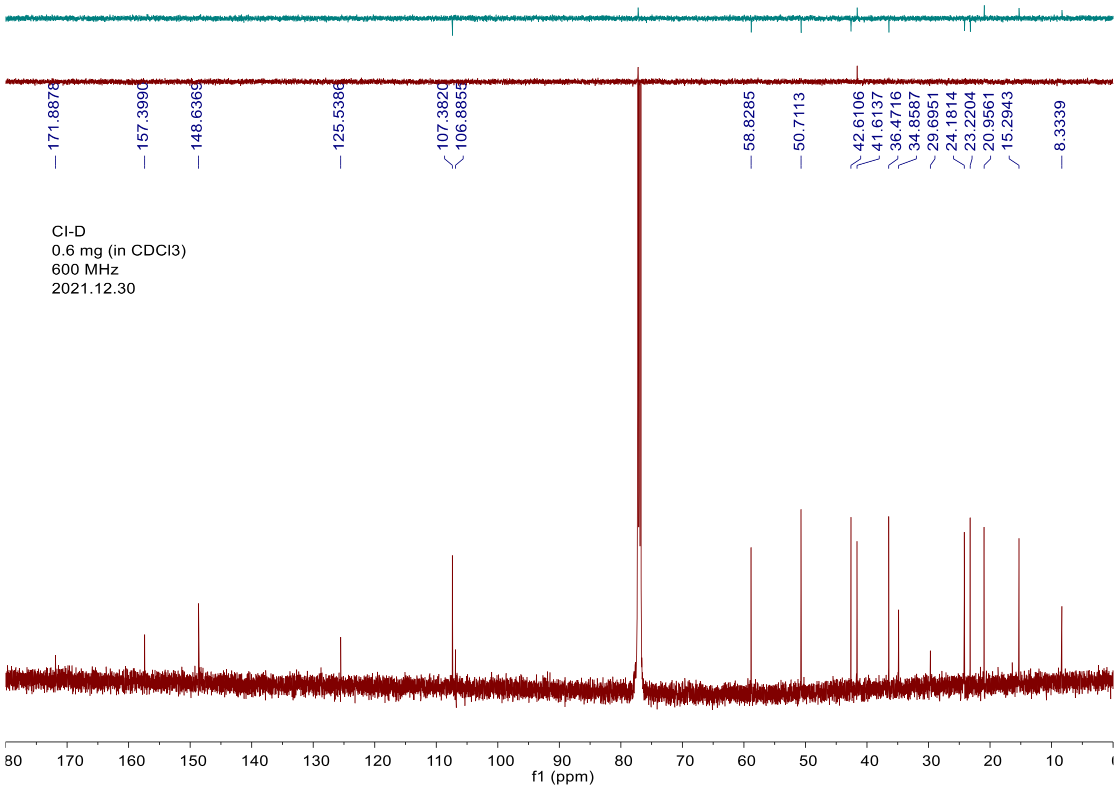

Figure S14 ^{13}C NMR spectrum of **2** (125 MHz, $CDCl_3$)

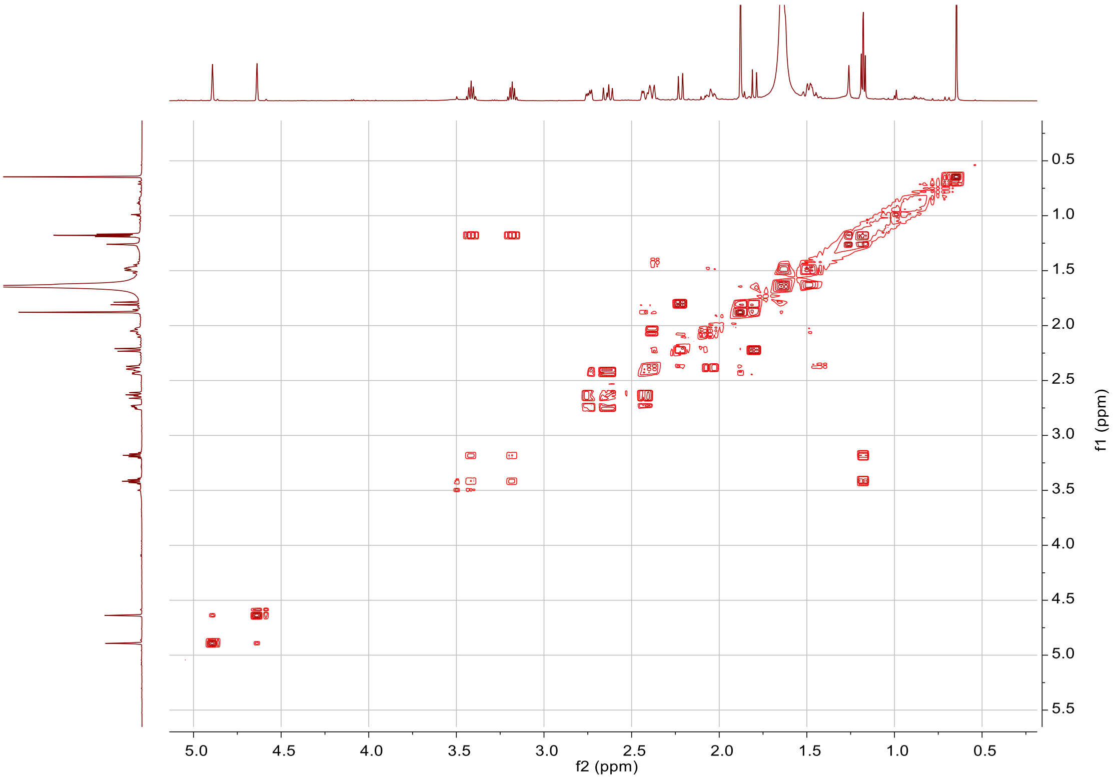

Figure S15 COSY spectrum of **2**

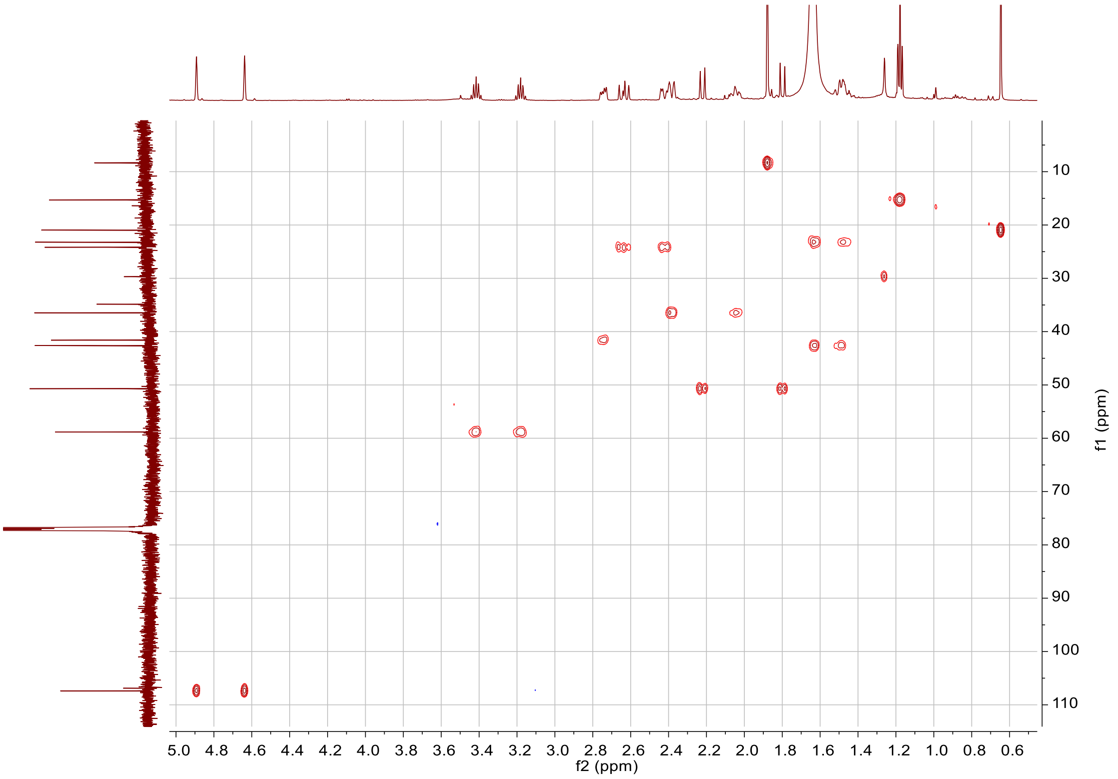

Figure S16 HSQC spectrum of **2**

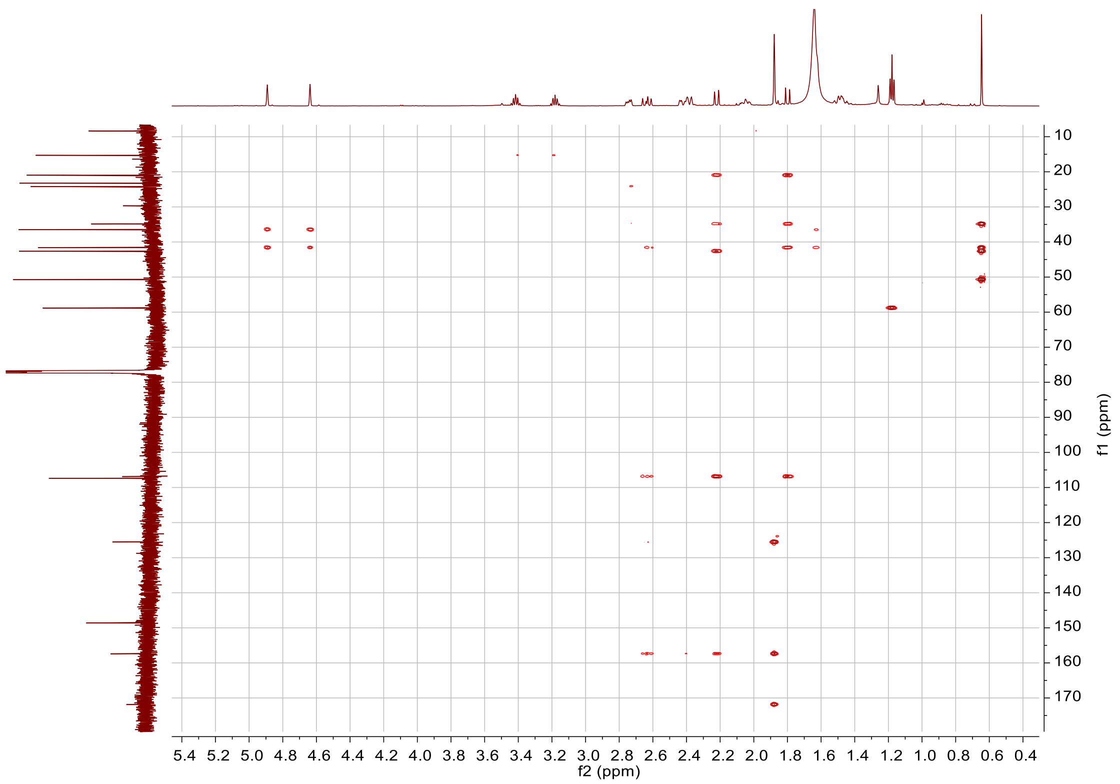

Figure S17 HMBC spectrum of **2**

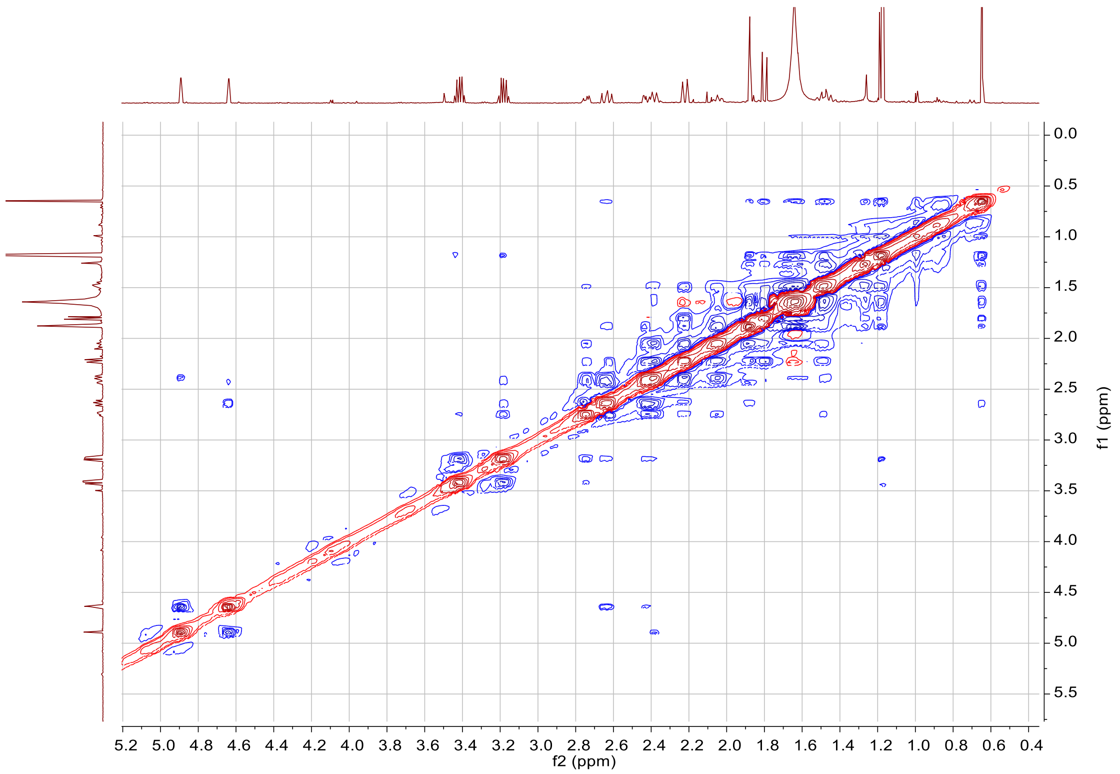

Figure S18 NOESY spectrum of **2**

Mass Spectrum SmartFormula Report

Analysis Info

Analysis Name	D:\1107\CIDR1.d	11/8/2022 2:41:15 PM
Method	tune_wide_pos_20220422.m	Operator: YU HSIAO-CHING
Sample Name	CI-D	Instrument: BRUKER micrOTOF-Q
Comment	ESI Positive	

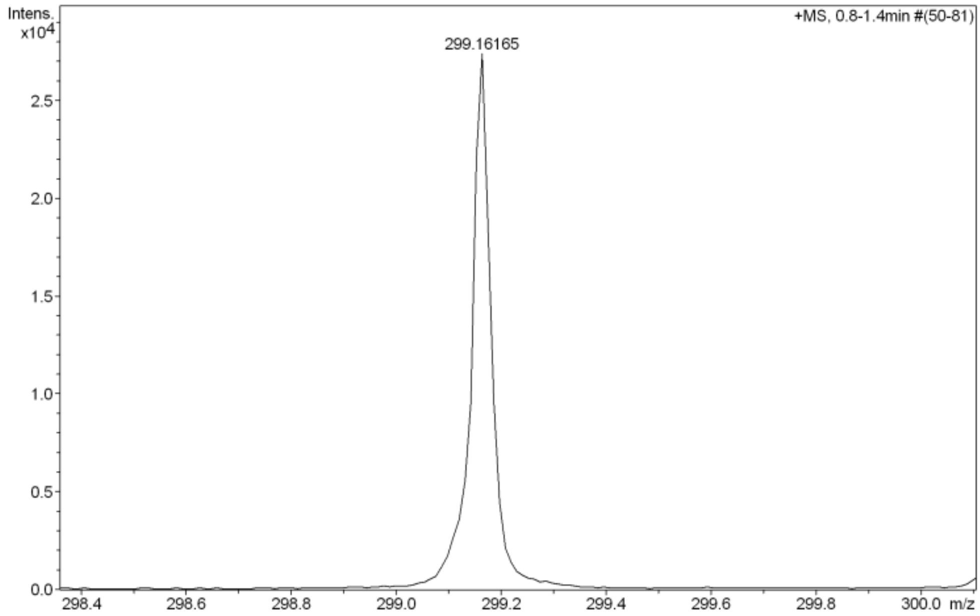

Meas. m/z	#	Formula	Score	m/z	err [mDa]	err [ppm]	mSigma	rdb	e⁻ Conf	N-Rule
299.16165	1	C 17 H 24 Na O 3	100.00	299.16177	0.11	0.38	35.6	5.5	even	ok

Figure S19 HRESIMS spectrum of **2**

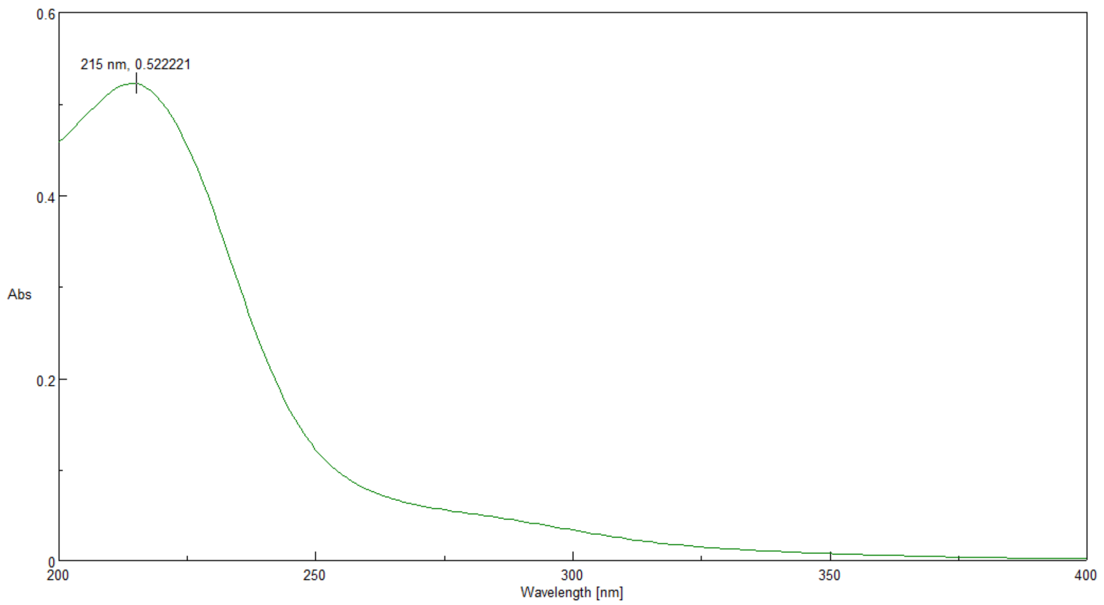

Figure S20 UV spectrum of **2**

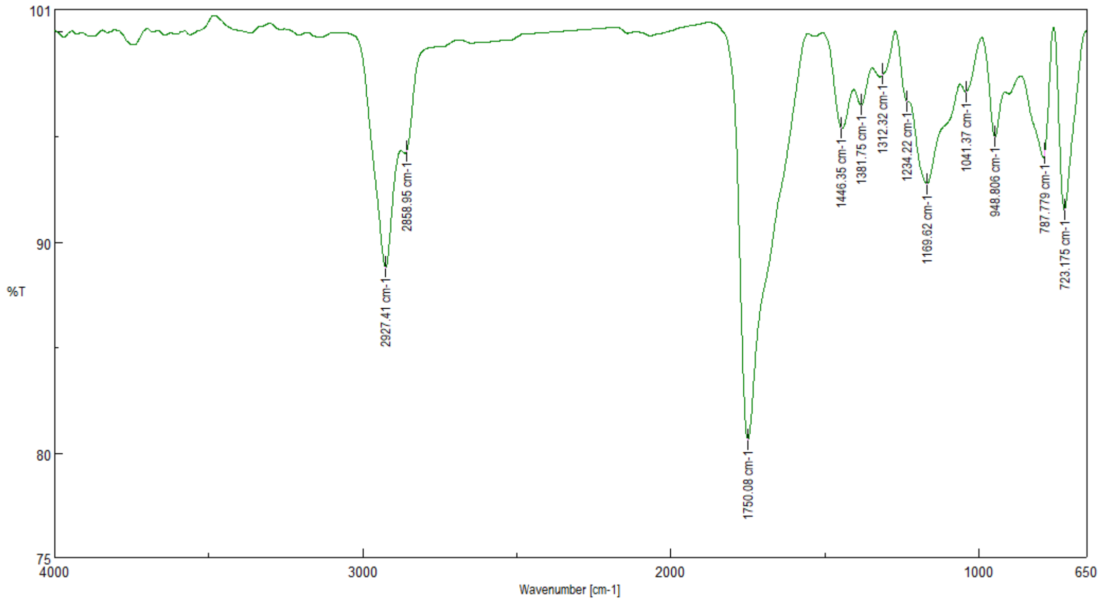

Figure S21 IR spectrum of **2**

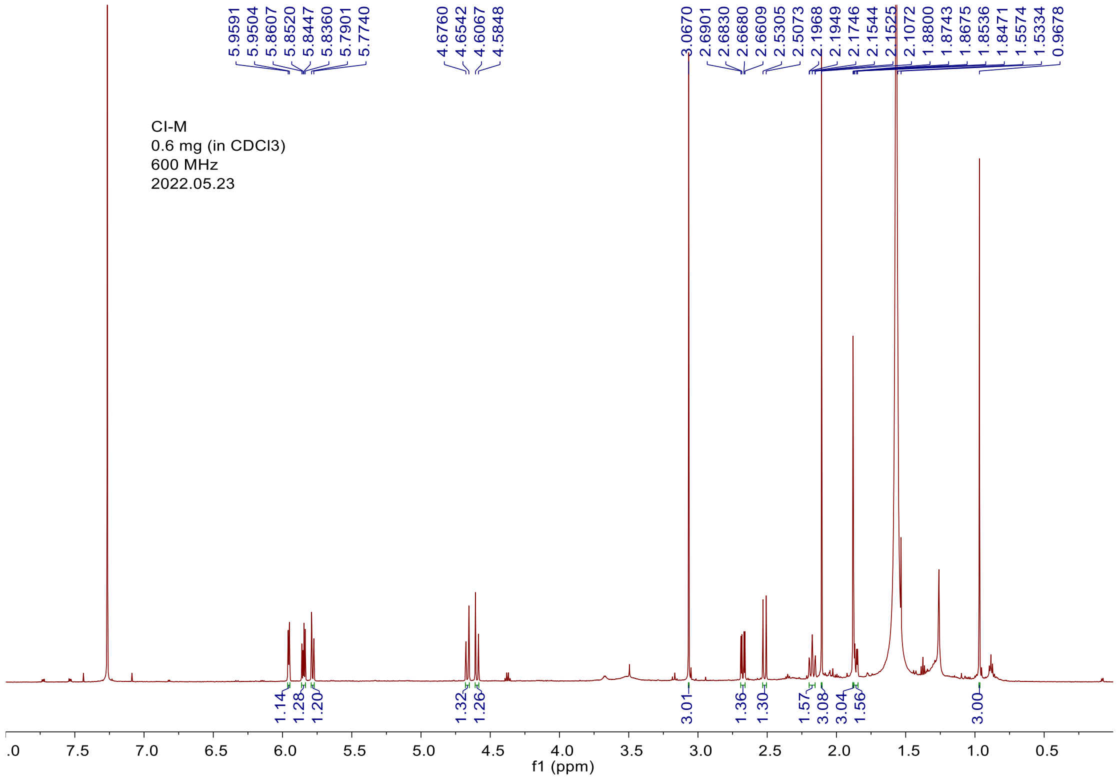

Figure S22 ^{1}H NMR spectrum of **3** (600 MHz, $CDCl_3$)

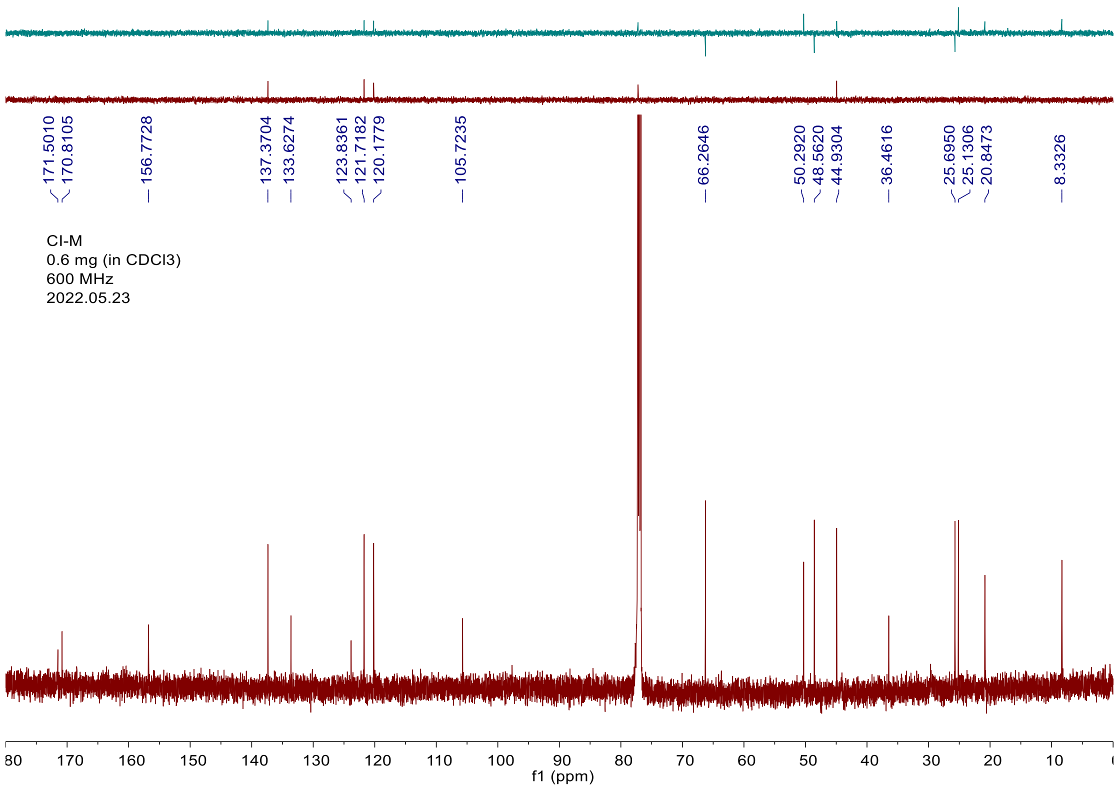

Figure S23 ^{13}C NMR spectrum of **3** (125 MHz, $CDCl_3$)

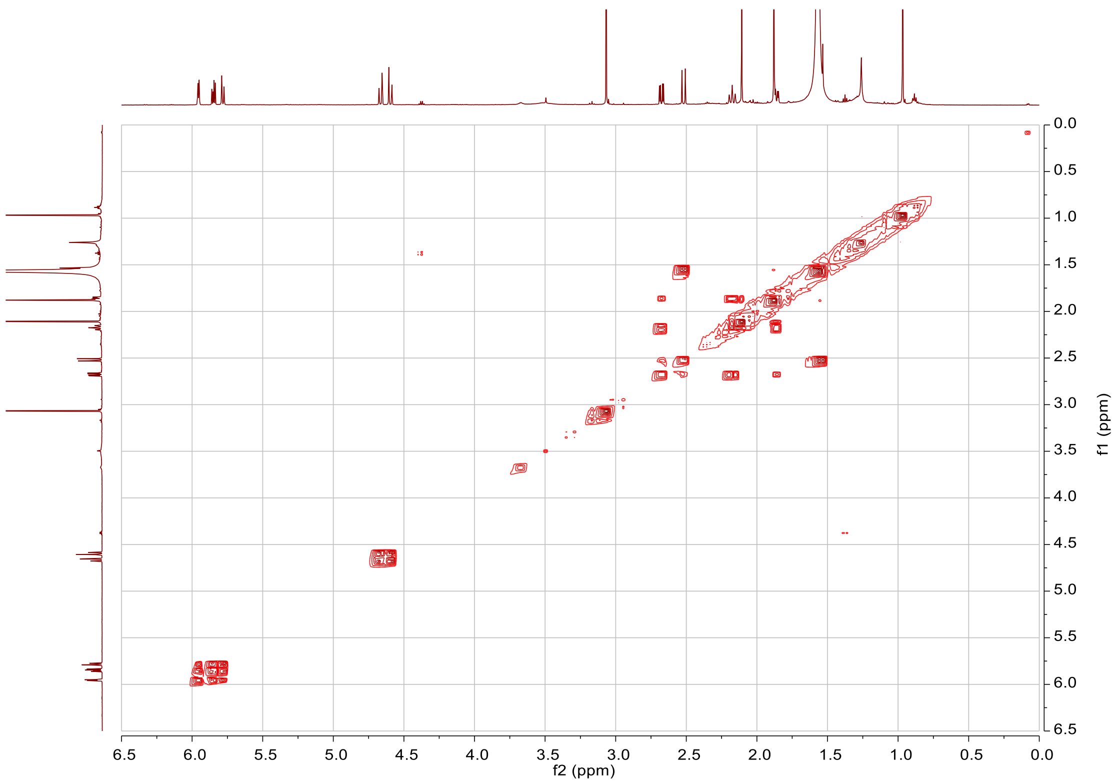

Figure S24 COSY spectrum of **3**

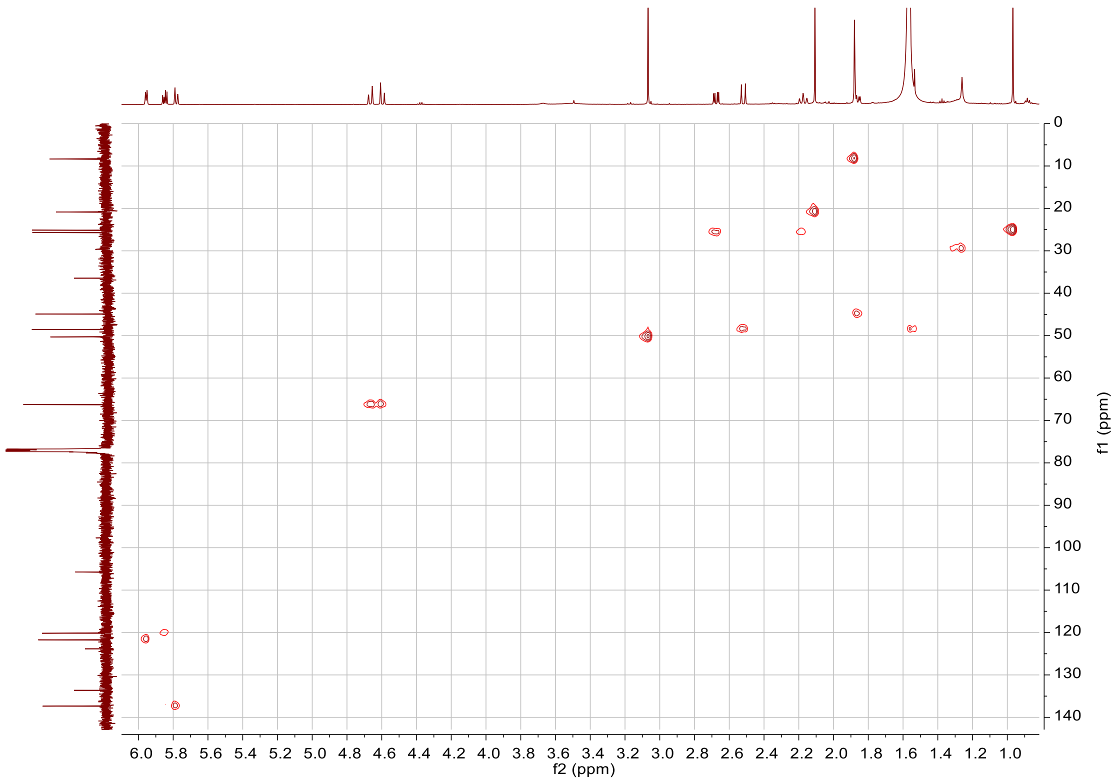

Figure S25 HSQC spectrum of **3**

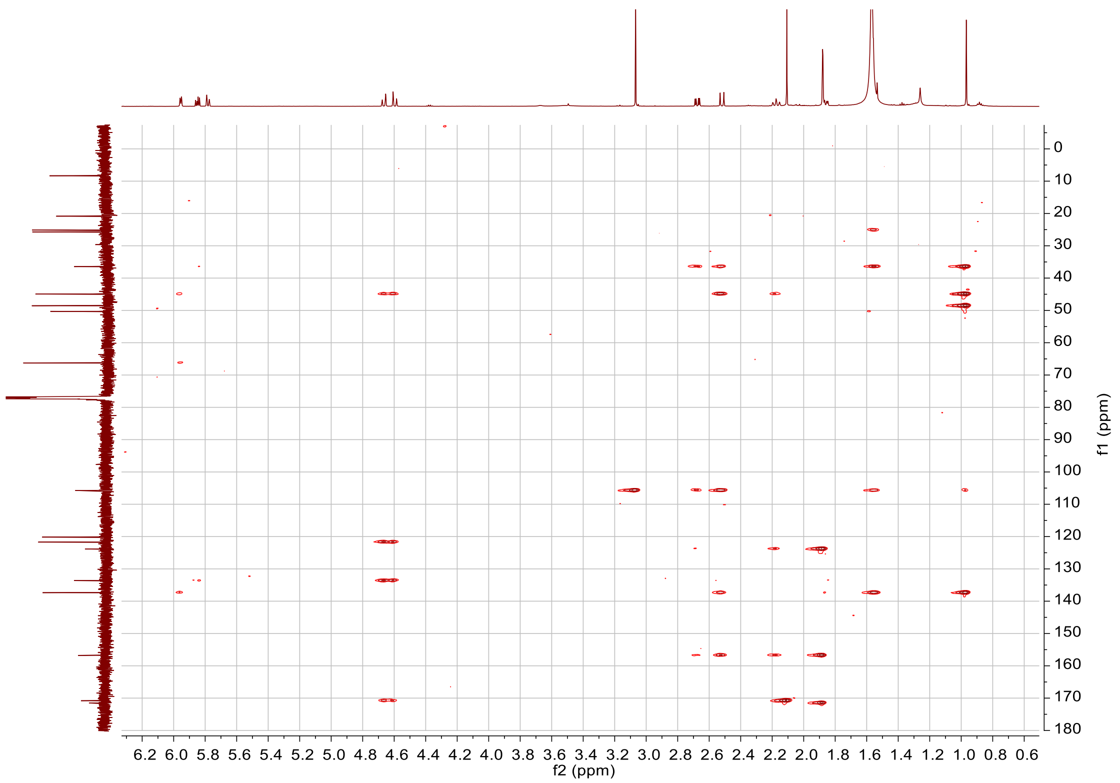

Figure S26 HMBC spectrum of **3**

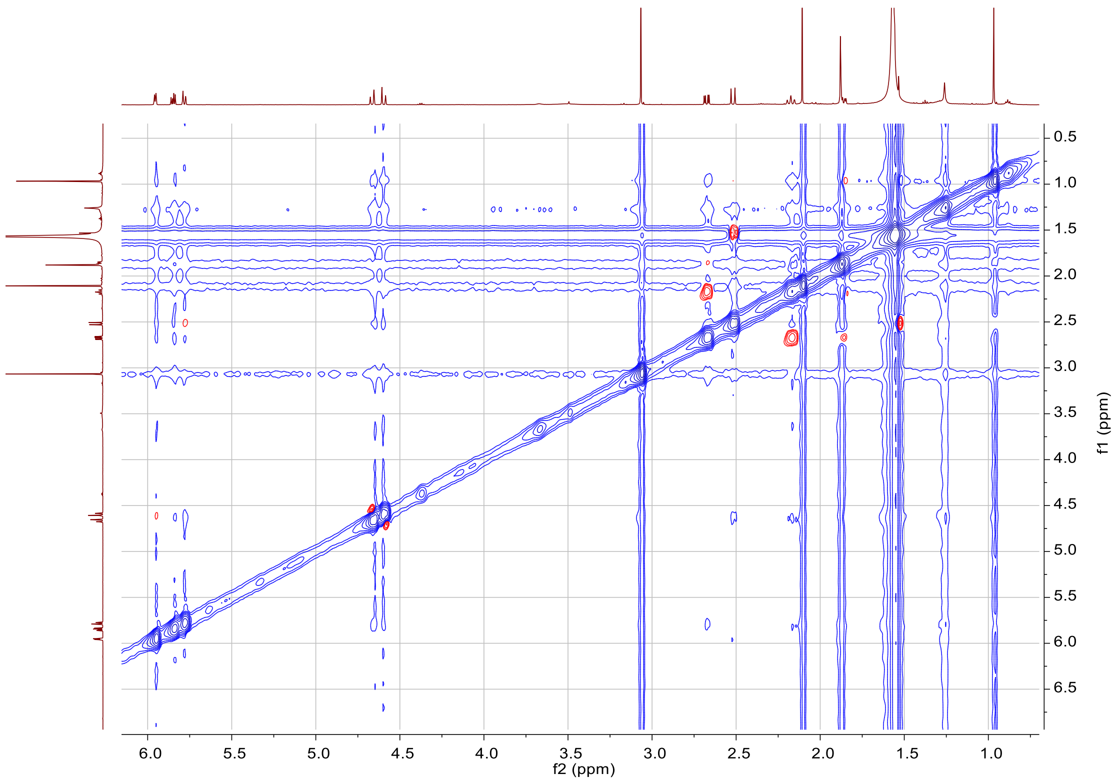

Figure S27 NOESY spectrum of **3**

Mass Spectrum SmartFormula Report

Analysis Info

Analysis Name	D:\0819\CIMR.d	8/23/2022 11:57:34 AM
Method	tune_wide_pos_20220422.m	Operator: YU HSIAO-CHING
Sample Name	CI-M	Instrument: BRUKER micrOTOF-Q
Comment	Positive	

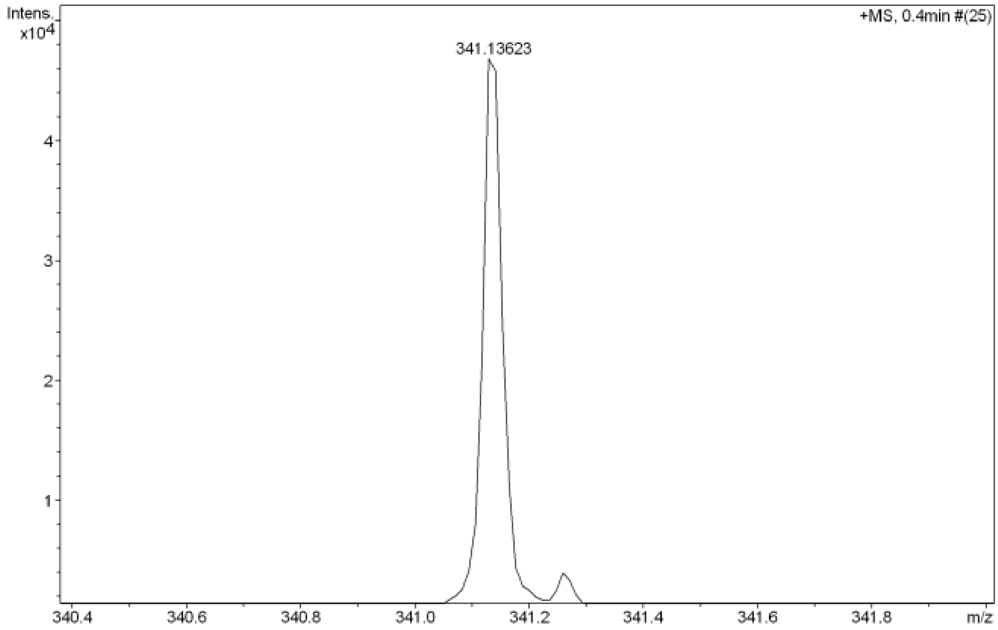

Meas. m/z	#	Formula	Score	m/z	err [mDa]	err [ppm]	mSigma	rdb	e⁻ Conf	N-Rule
341.13623	1	C 18 H 22 Na O 5	100.00	341.13594	-0.29	-0.84	31.7	7.5	even	ok

Figure S28 HRESIMS spectrum of **3**

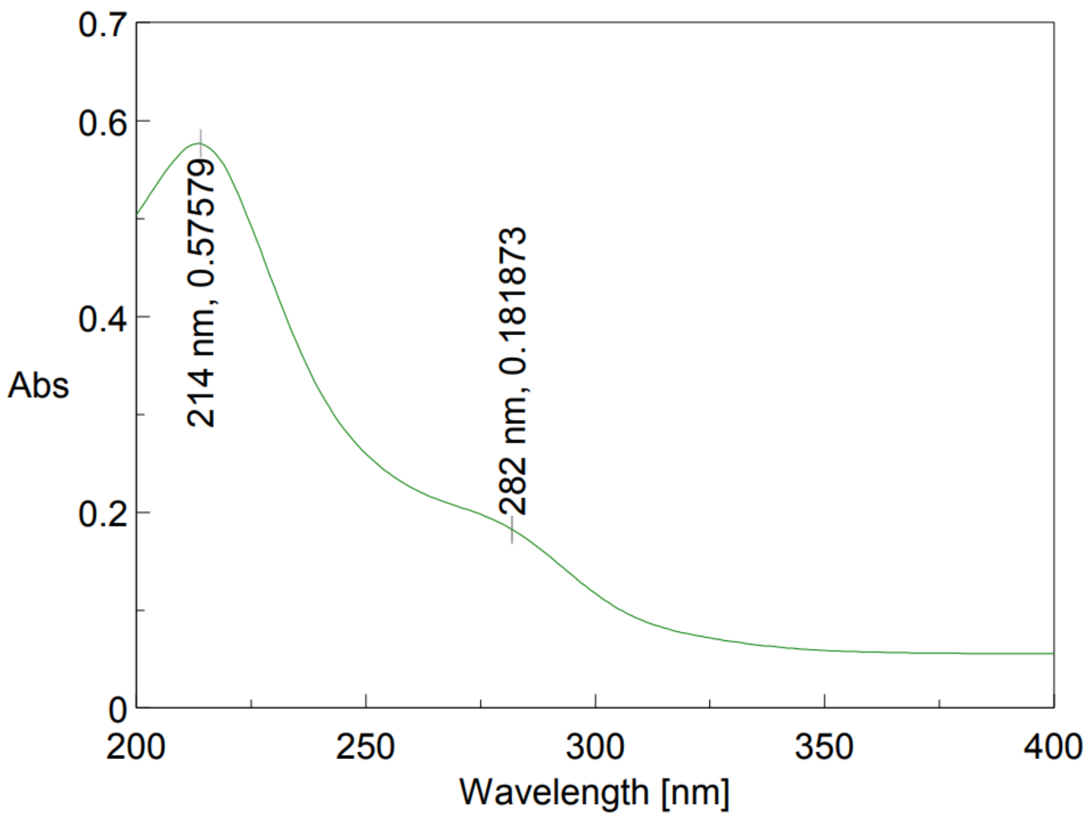

Figure S29 UV spectrum of **3**

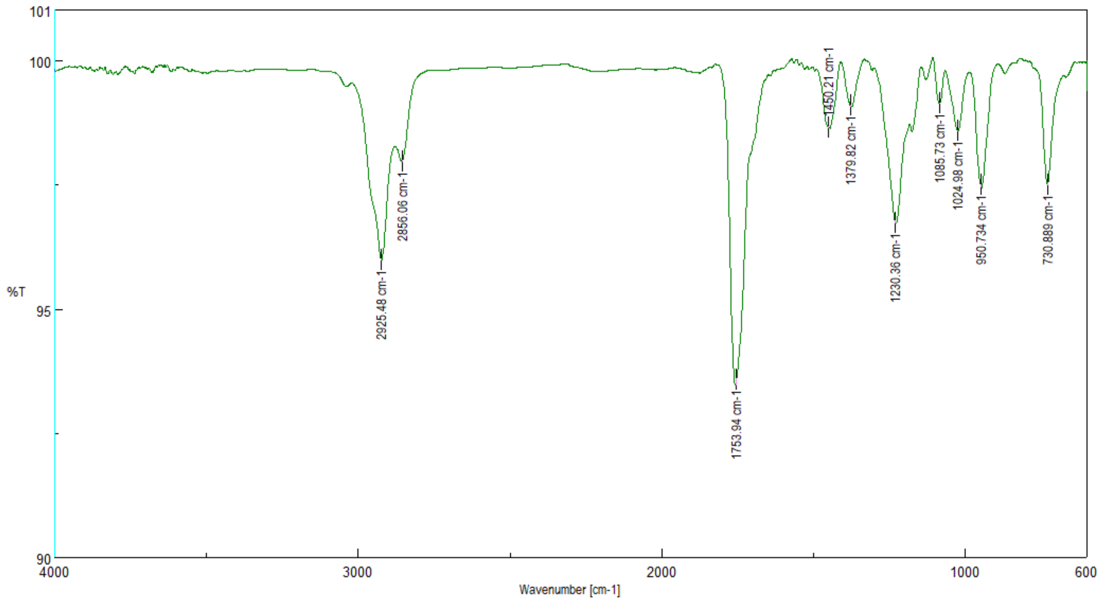

Figure S30 IR spectrum of **3**

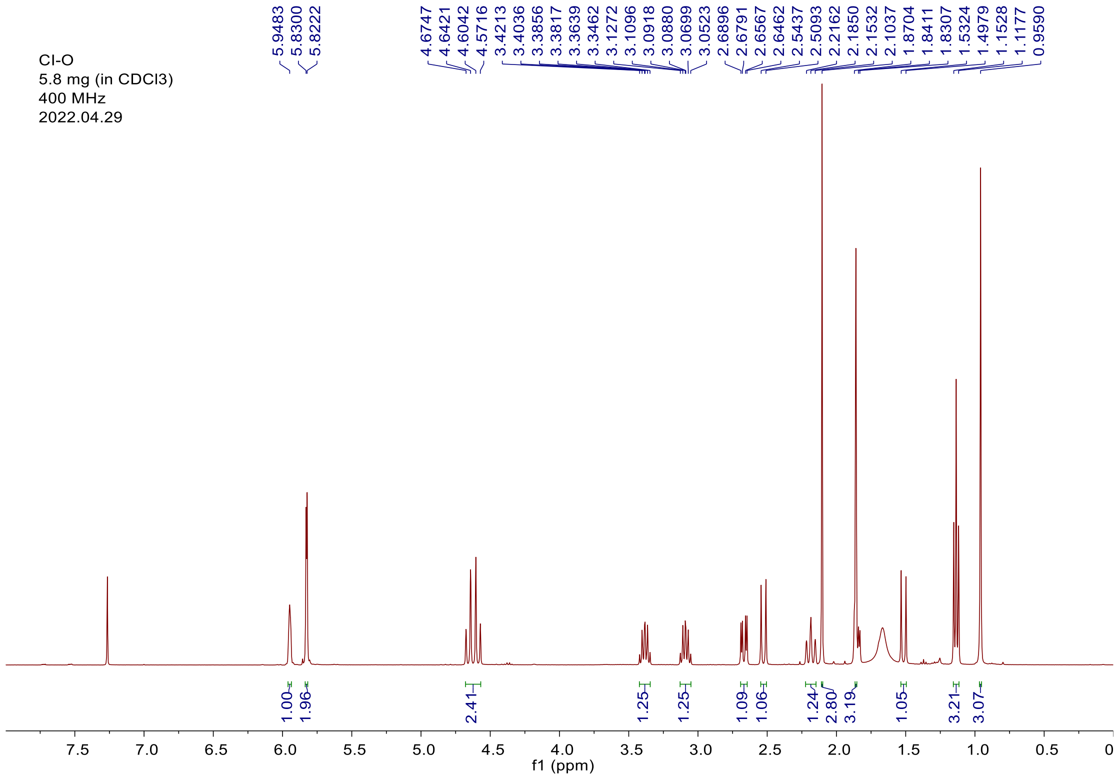

Figure S31 ^{1}H NMR spectrum of **4** (400 MHz, $CDCl_3$)

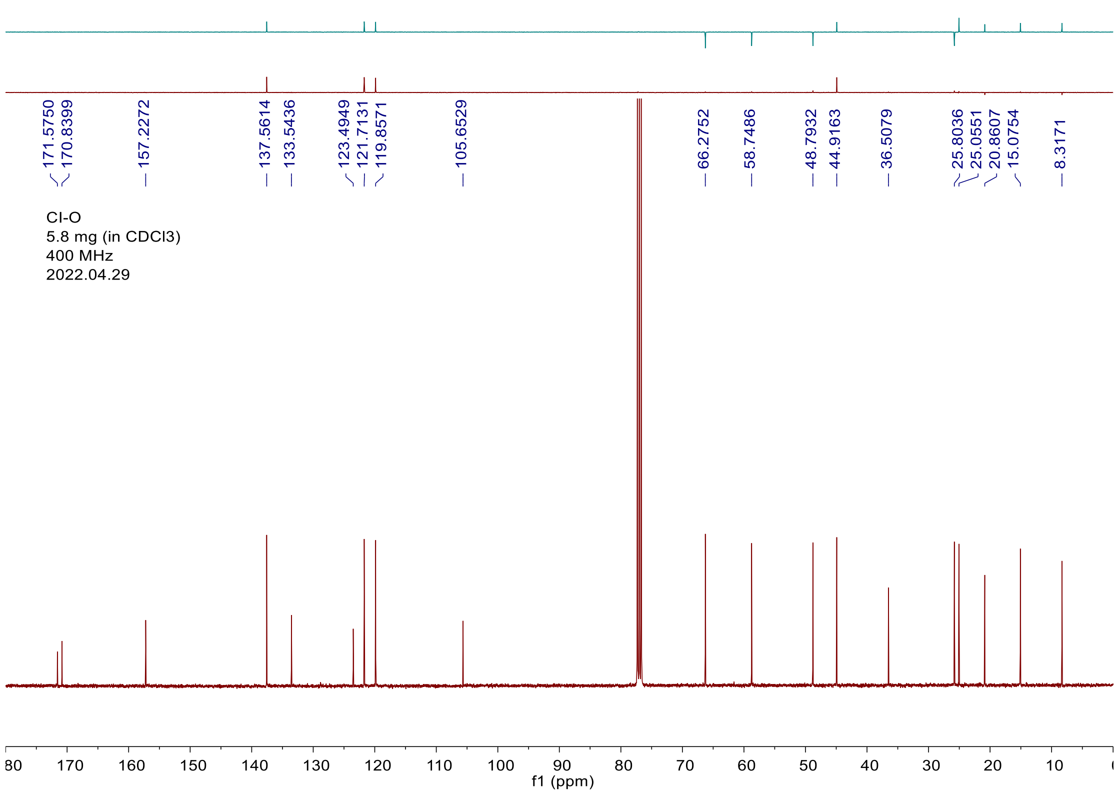

Figure S32 ^{13}C NMR spectrum of **4** (100 MHz, $CDCl_3$)

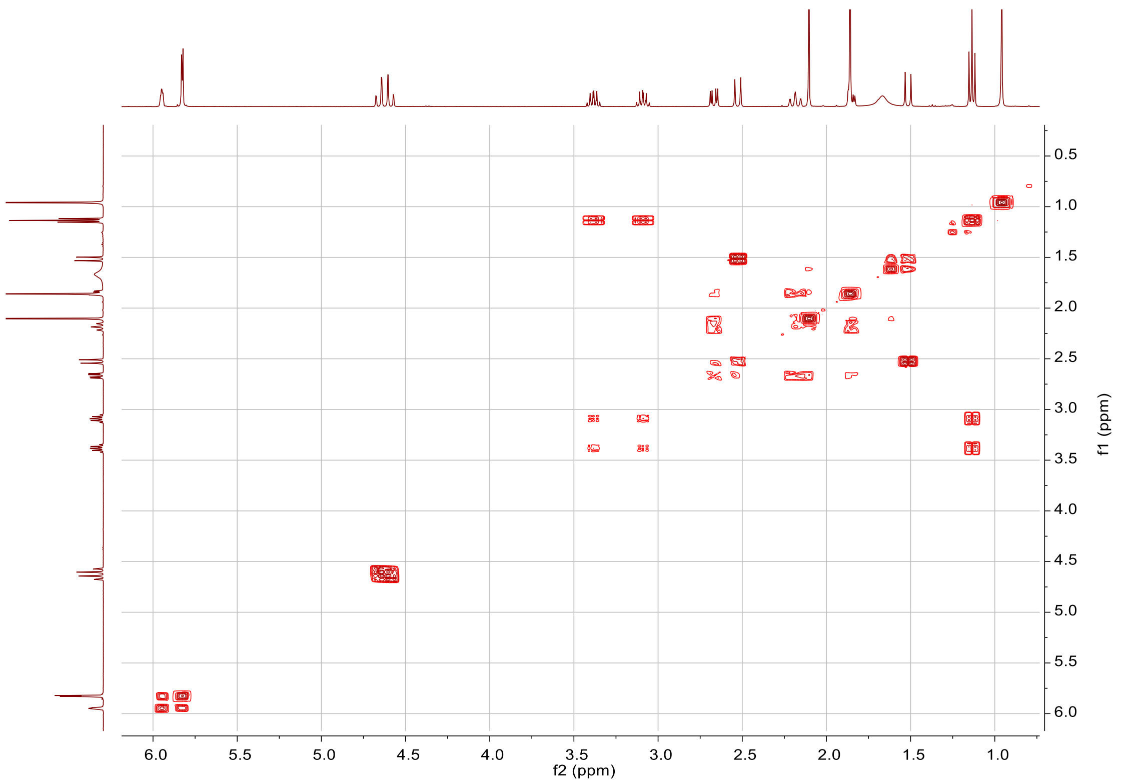

Figure S33 COSY spectrum of **4**

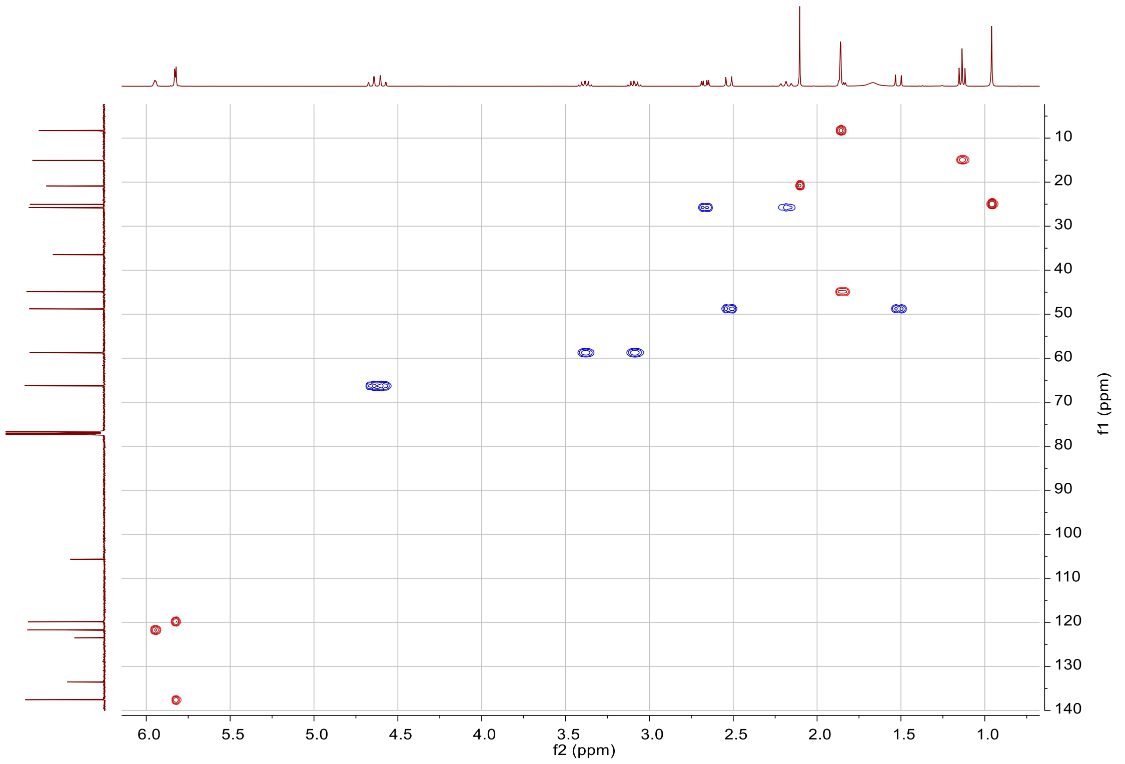

Figure S34 HSQC spectrum of **4**

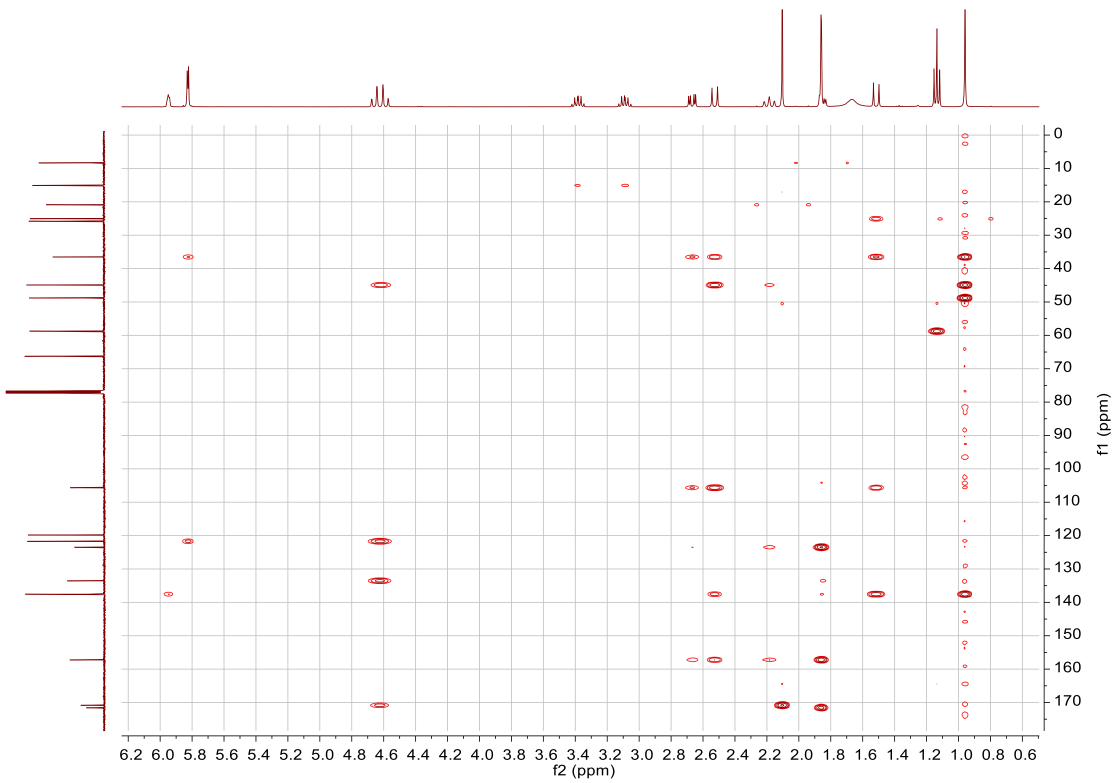

Figure S35 HMBC spectrum of **4**

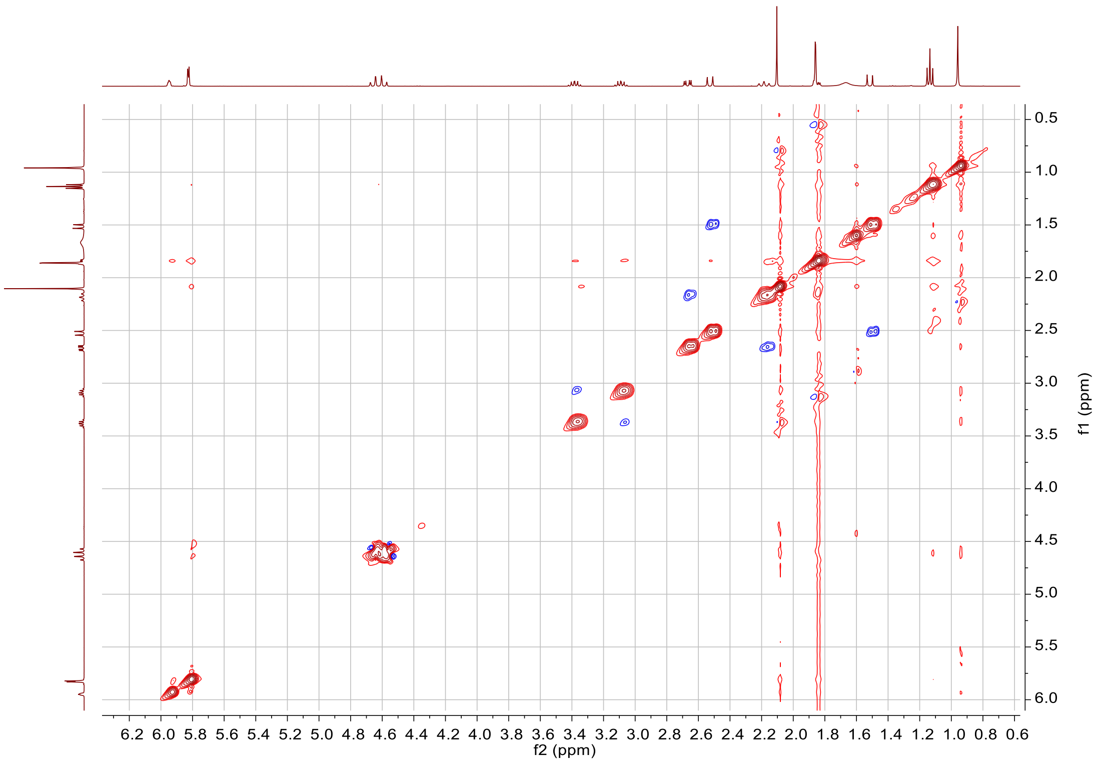

Figure S36 NOESY spectrum of **4**

Mass Spectrum SmartFormula Report

Analysis Info

Analysis Name	D:\0819\CIOR1.d	8/23/2022 11:59:58 AM
Method	tune_wide_pos_20220422.m	Operator: YU HSIAO-CHING
Sample Name	CI-O	Instrument: BRUKER micrOTOF-Q
Comment	Positive	

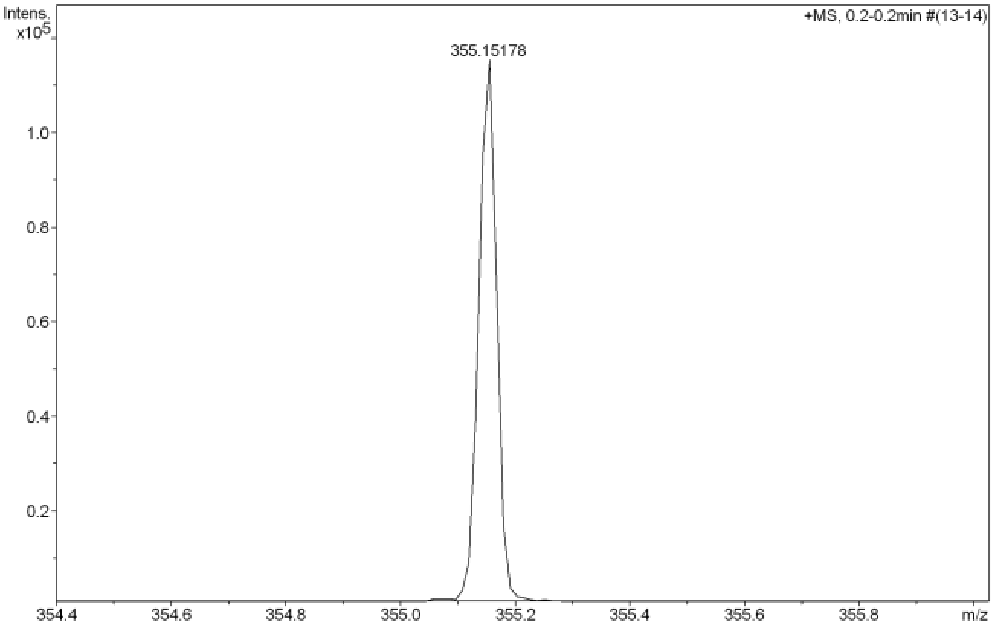

Meas. m/z	#	Formula	Score	m/z	err [mDa]	err [ppm]	mSigma	rdb	e⁻ Conf	N-Rule
355.15178	1	C 19 H 24 Na O 5	100.00	355.15159	-0.19	-0.52	25.5	7.5	even	ok

Figure S37 HRESIMS spectrum of **4**

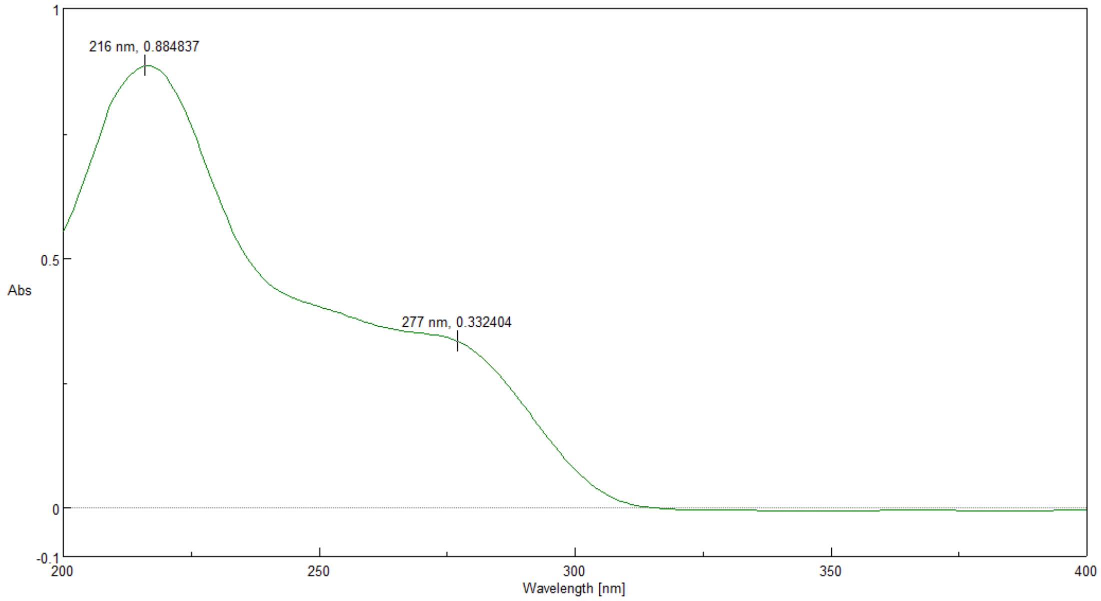

Figure S38 UV spectrum of **4**

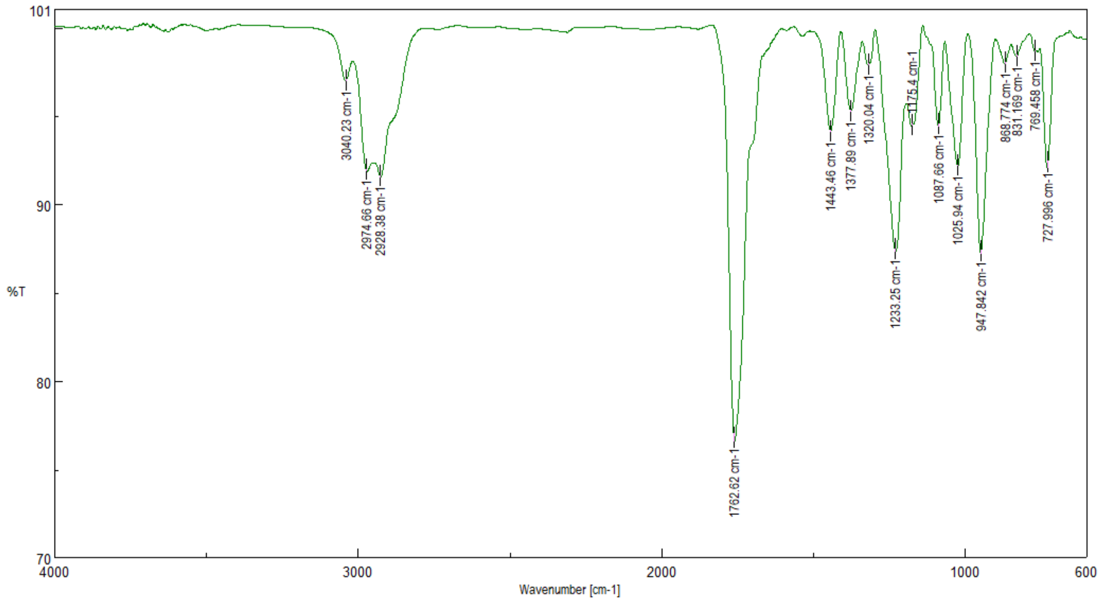

Figure S39 IR spectrum of **4**

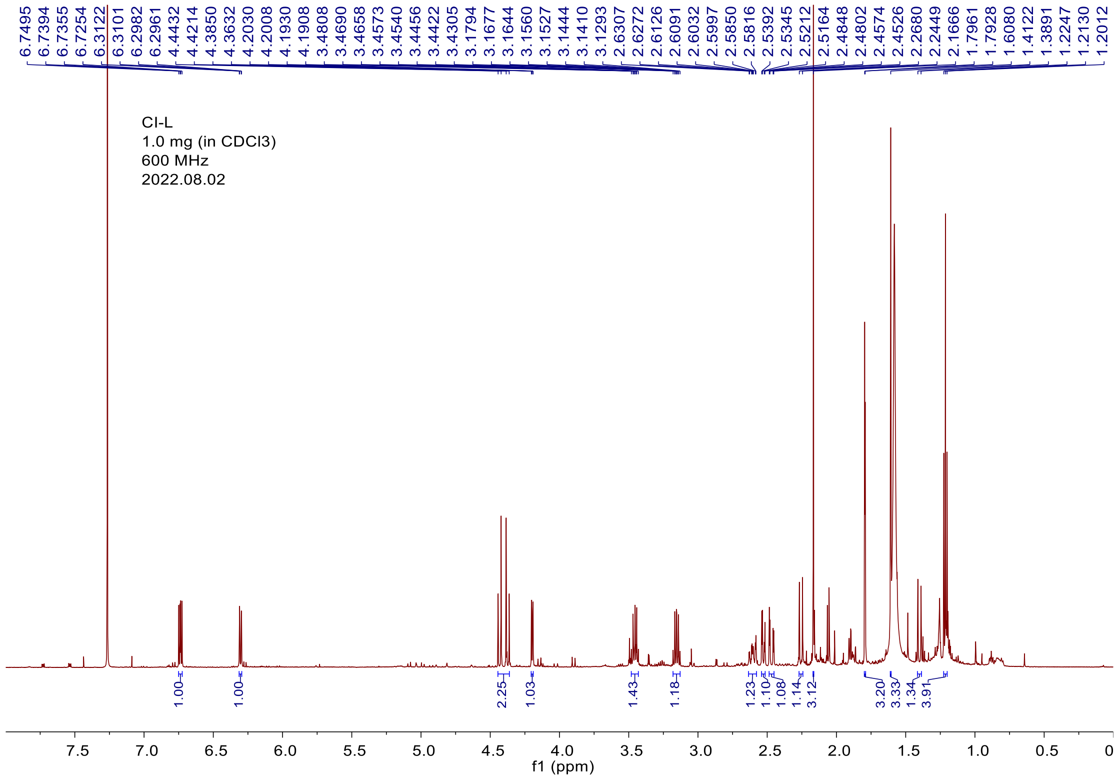

Figure S40 ^{1}H NMR spectrum of **5** (600 MHz, $CDCl_3$)

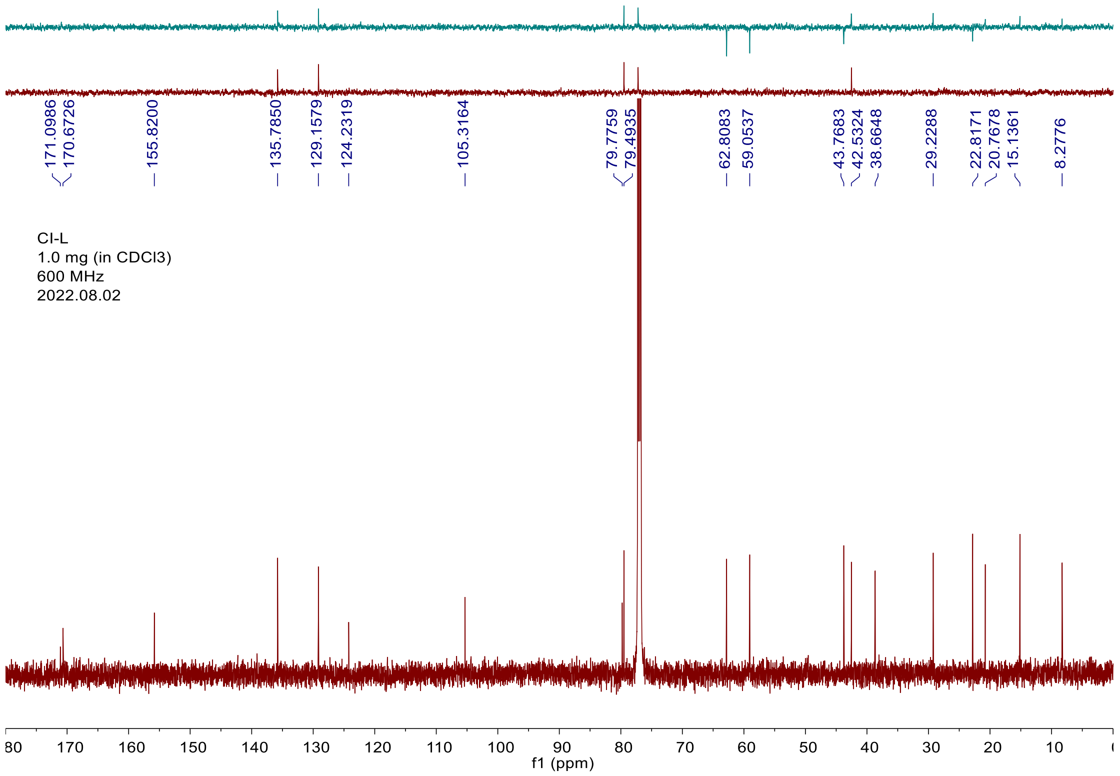

Figure S41 ^{13}C NMR spectrum of **5** (125 MHz, $CDCl_3$)

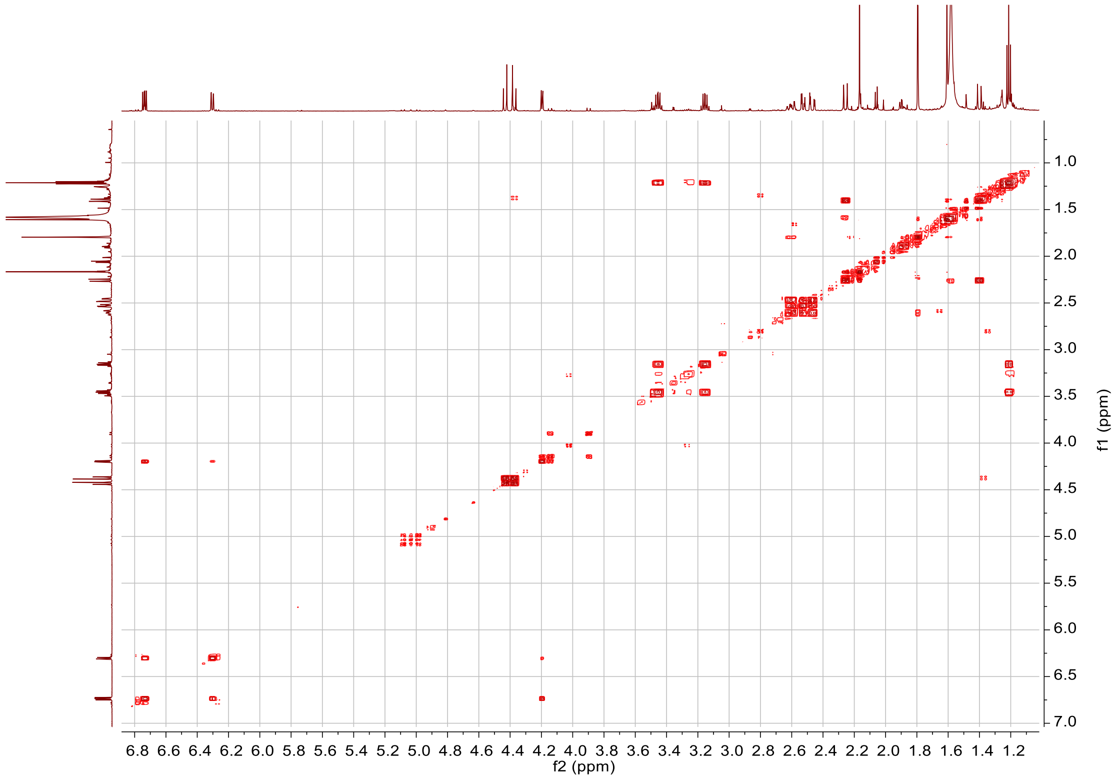

Figure S42 COSY spectrum of **5**

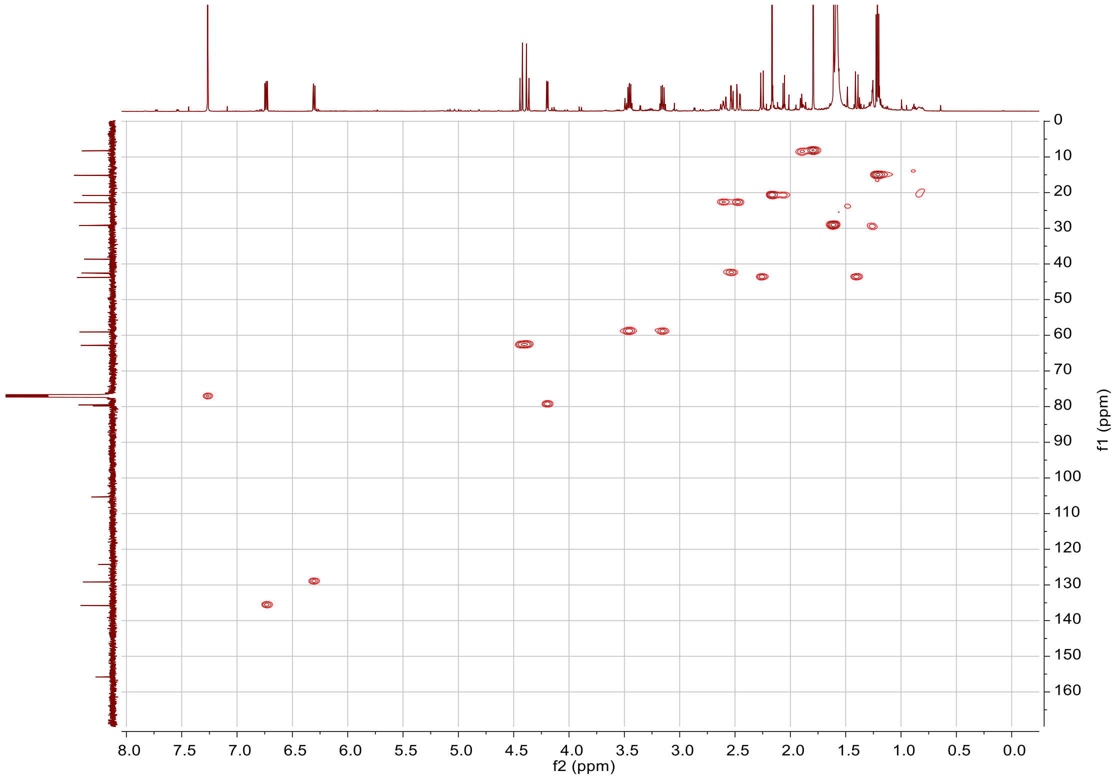

Figure S43 HSQC spectrum of **5**

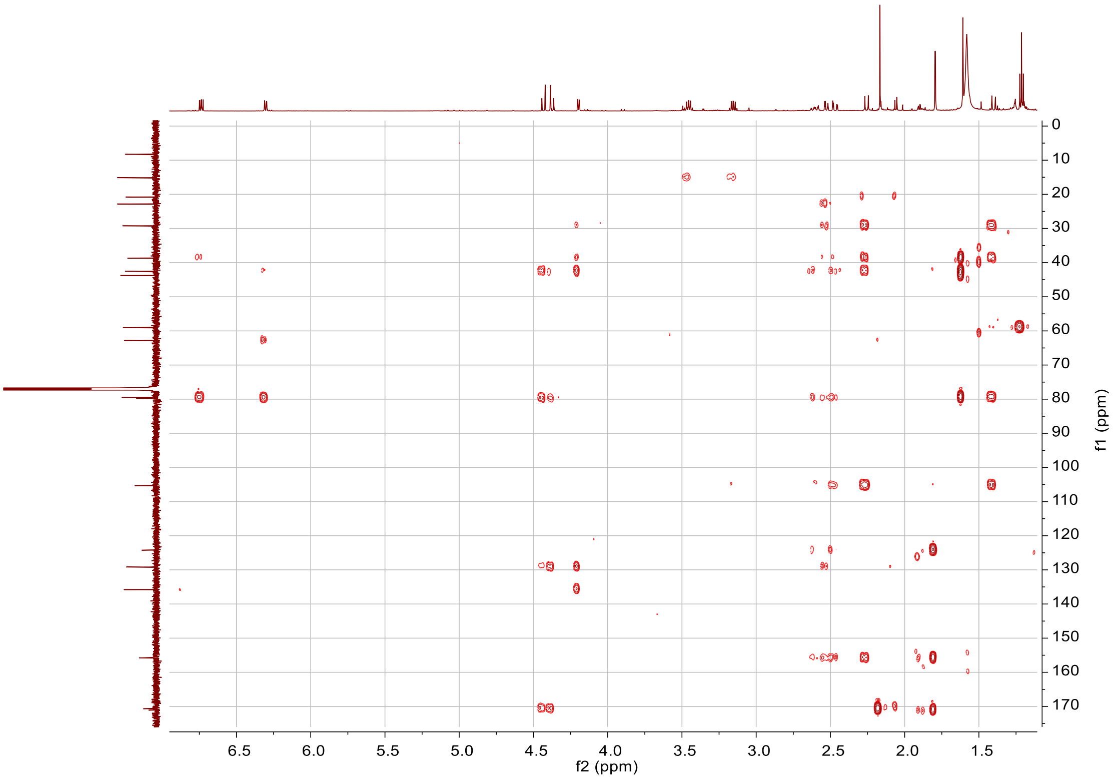

Figure S44 HMBC spectrum of **5**

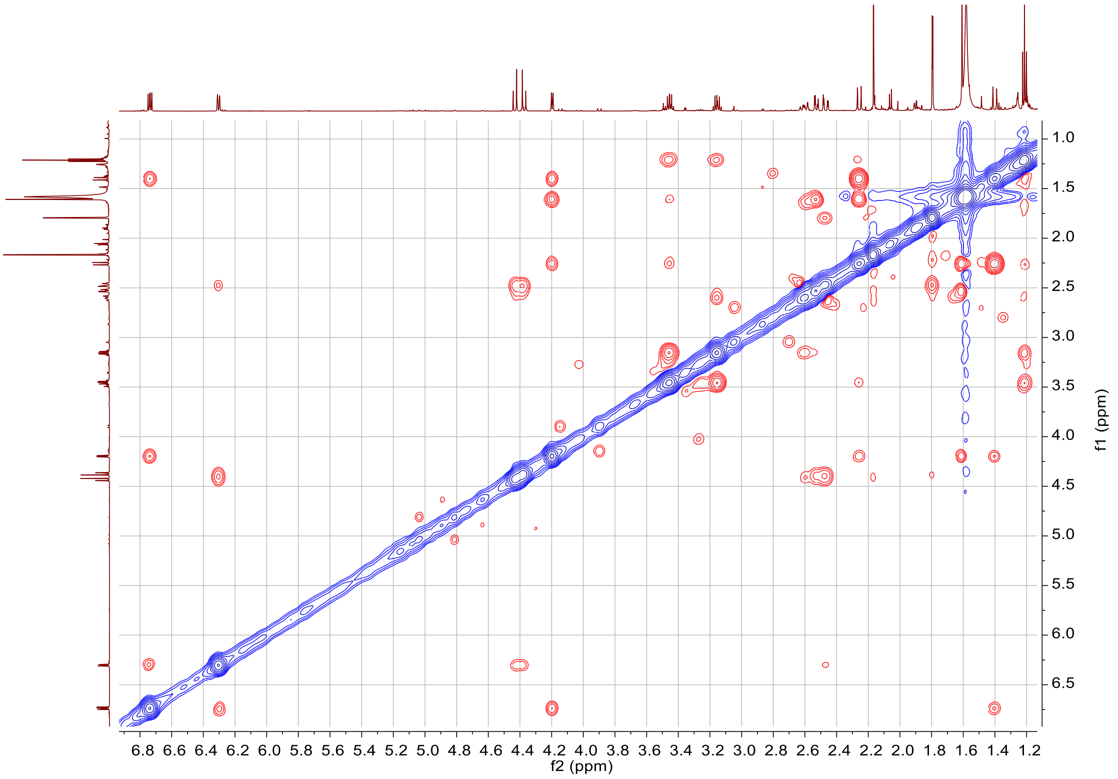

Figure S45 NOESY spectrum of **5**

Mass Spectrum SmartFormula Report

Analysis Info

Analysis Name D:\0819\CIMR.d
Method tune_wide_pos_20220422.m
Sample Name CI-M
Comment Positive

8/23/2022 11:57:34 AM
Operator: YU HSIAO-CHING
Instrument: BRUKER micrOTOF-Q

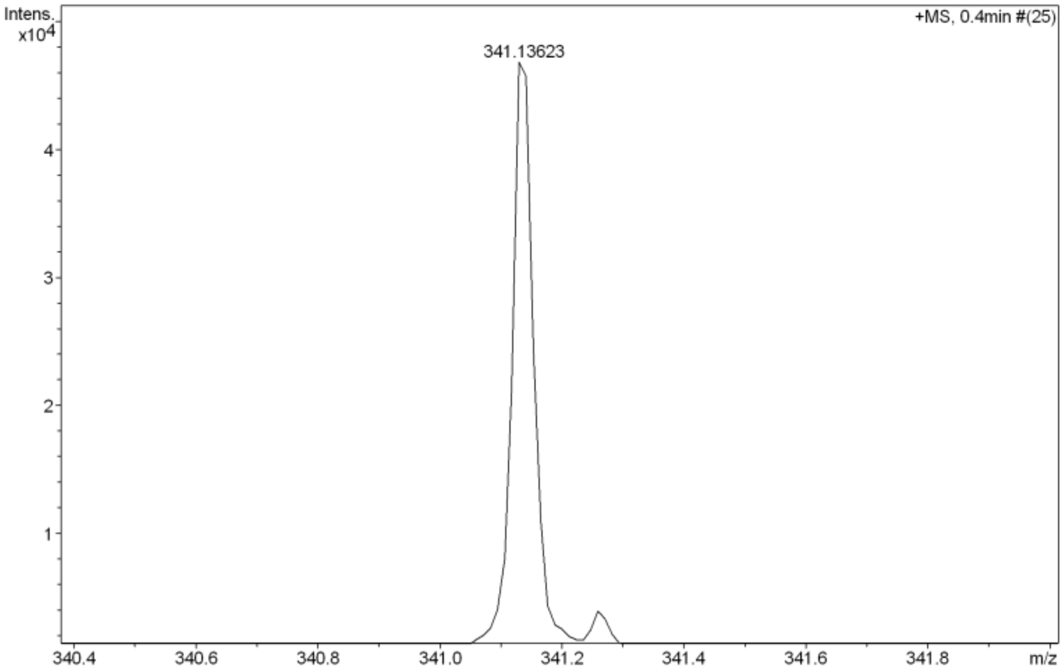

Meas. m/z	#	Formula	Score	m/z	err [mDa]	err [ppm]	mSigma	rdb	e⁻ Conf	N-Rule
341.13623	1	C 18 H 22 Na O 5	100.00	341.13594	-0.29	-0.84	31.7	7.5	even	ok

Figure S46 HRESIMS spectrum of **5**

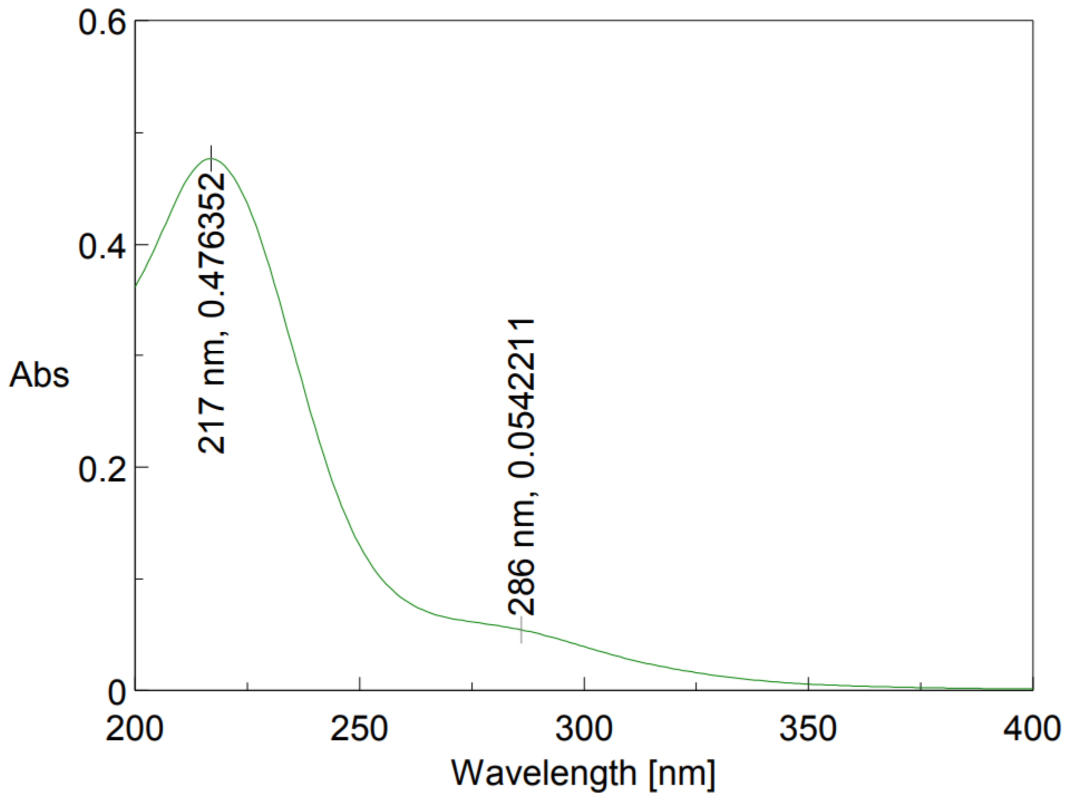

Figure S47 UV spectrum of **5**

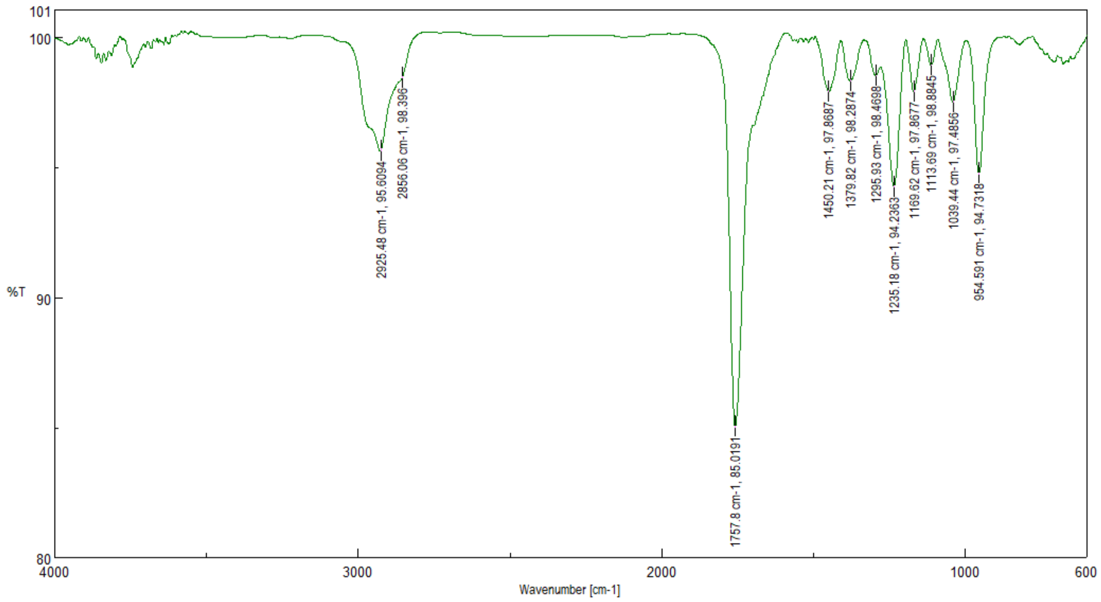

Figure S48 IR spectrum of **5**

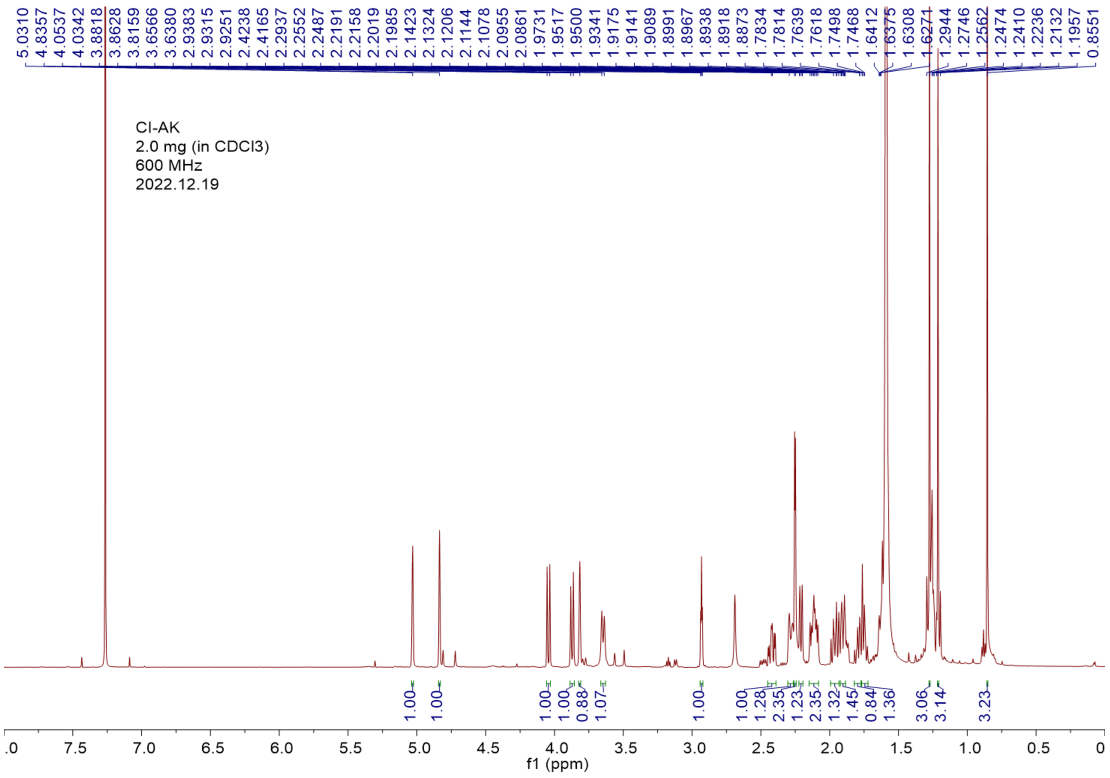

Figure S49 ^{1}H NMR spectrum of **6** (600 MHz, $CDCl_3$)

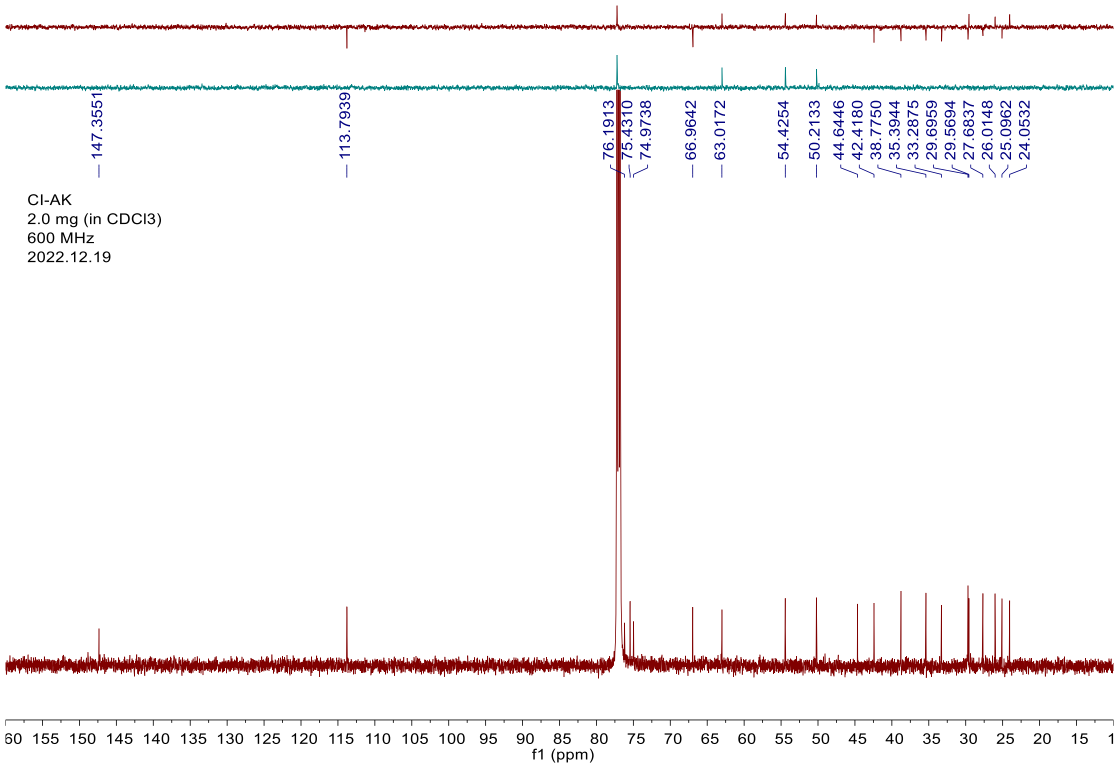

Figure S50 ^{13}C NMR spectrum of **6** (125 MHz, $CDCl_3$)

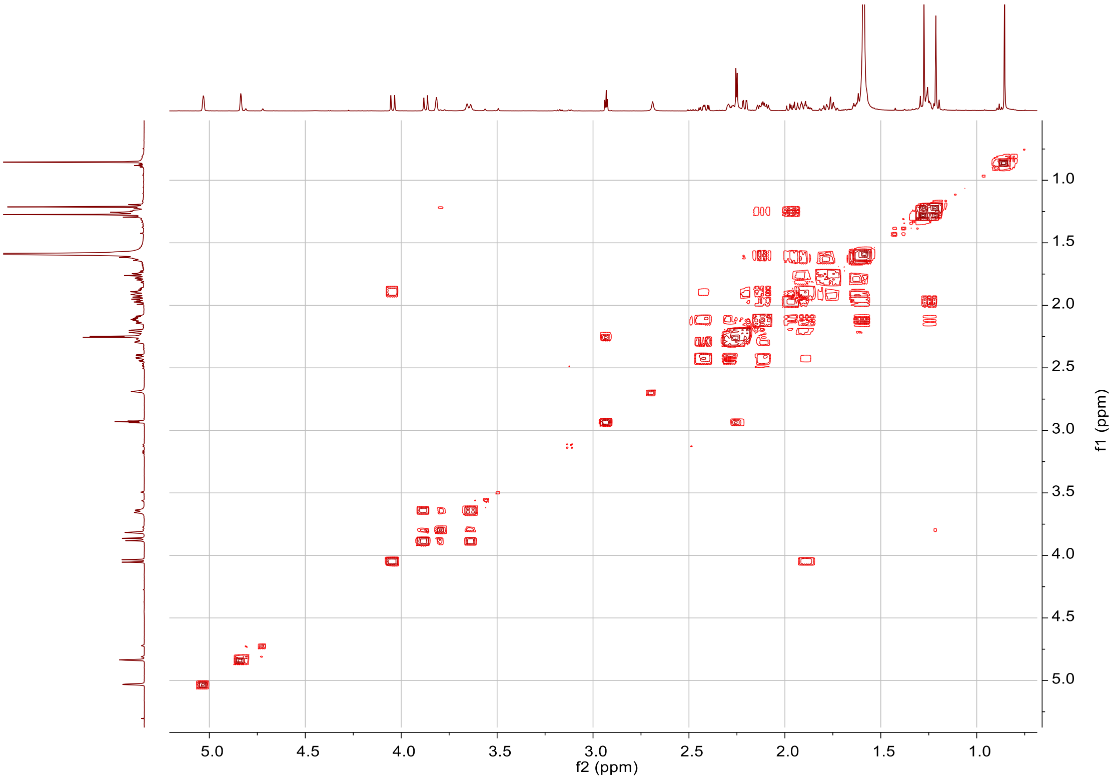

Figure S51 COSY spectrum of **6**

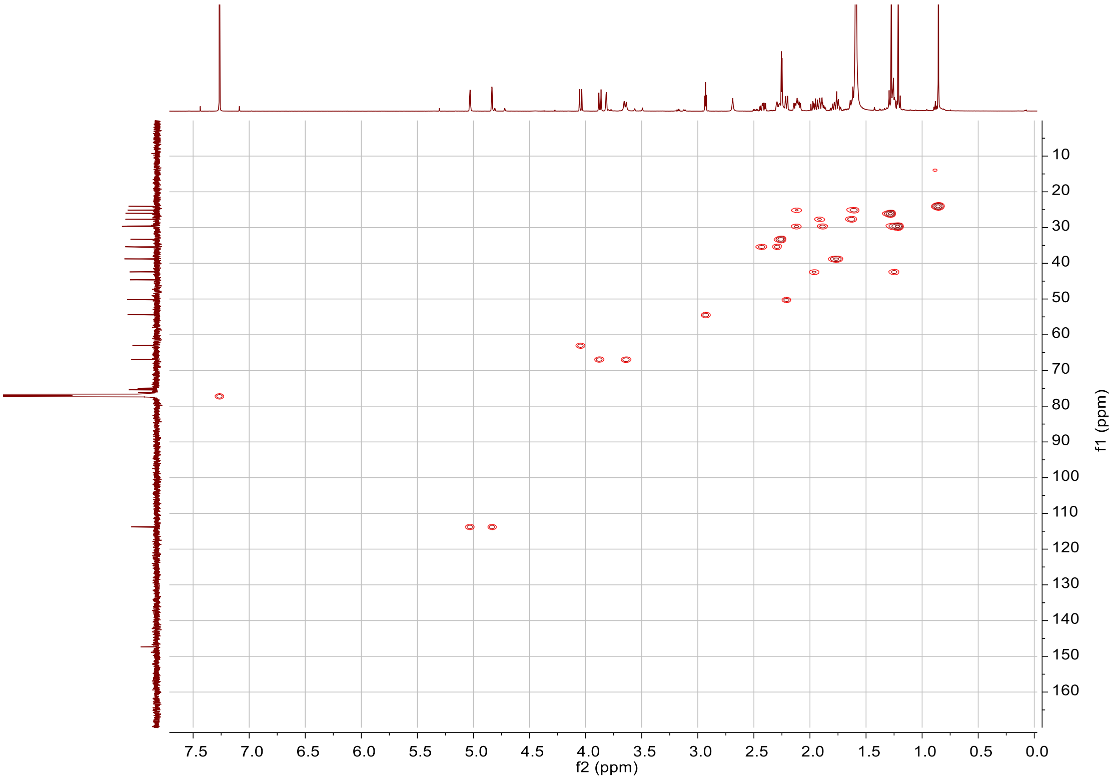

Figure S52 HSQC spectrum of **6**

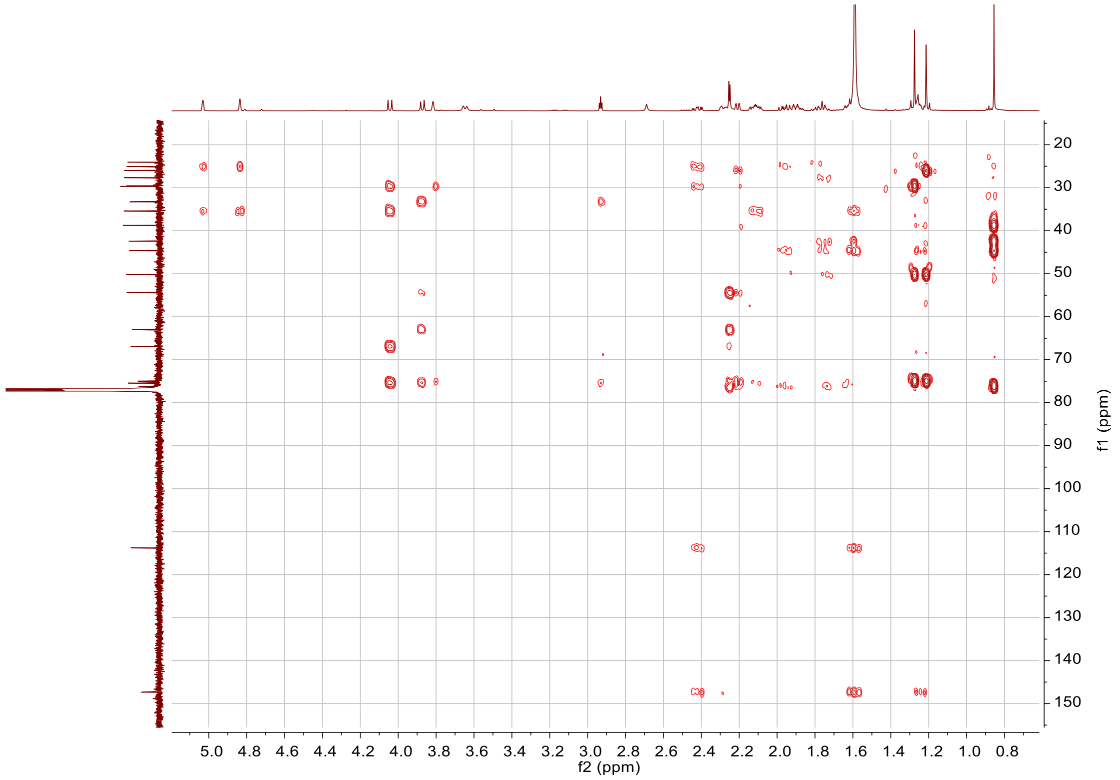

Figure S53 HMBC spectrum of **6**

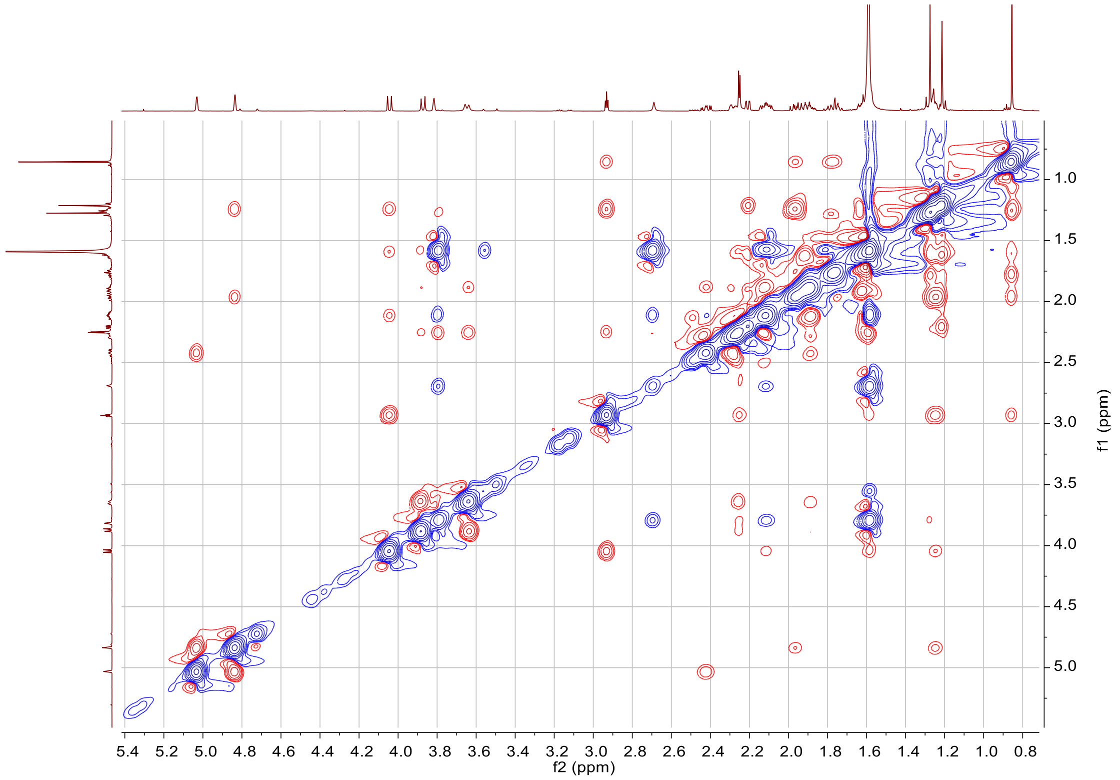

Figure S54 NOESY spectrum of **6**

Mass Spectrum SmartFormula Report

Analysis Info

Analysis Name	D:\1223\CIAKR.d	12/26/2022 11:30:15 AM
Method	tune_wide_pos_20220422.m	Operator: YU HSIAO-CHING
Sample Name	CI-AK	Instrument: BRUKER micrOTOF-Q
Comment	ESI Positive	

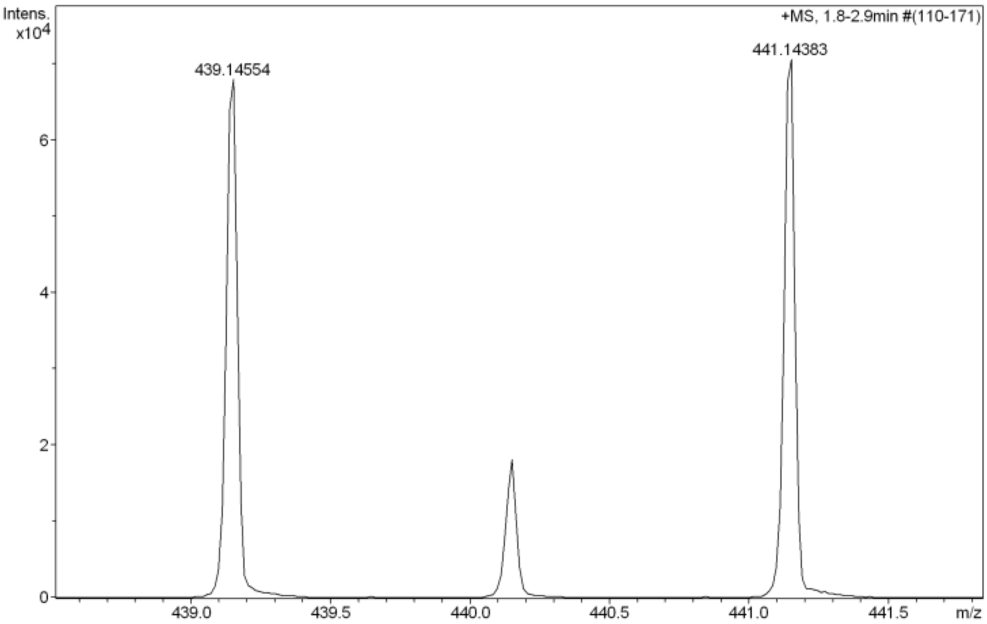

Meas. m/z	#	Formula	Score	m/z	err [mDa]	err [ppm]	mSigma	rdb	e⁻ Conf	N-Rule
439.14554	1	C 20 H 33 Br Na O 4	100.00	439.14544	-0.10	-0.22	23.8	3.5	even	ok

Figure S55 HRESIMS spectrum of **6**

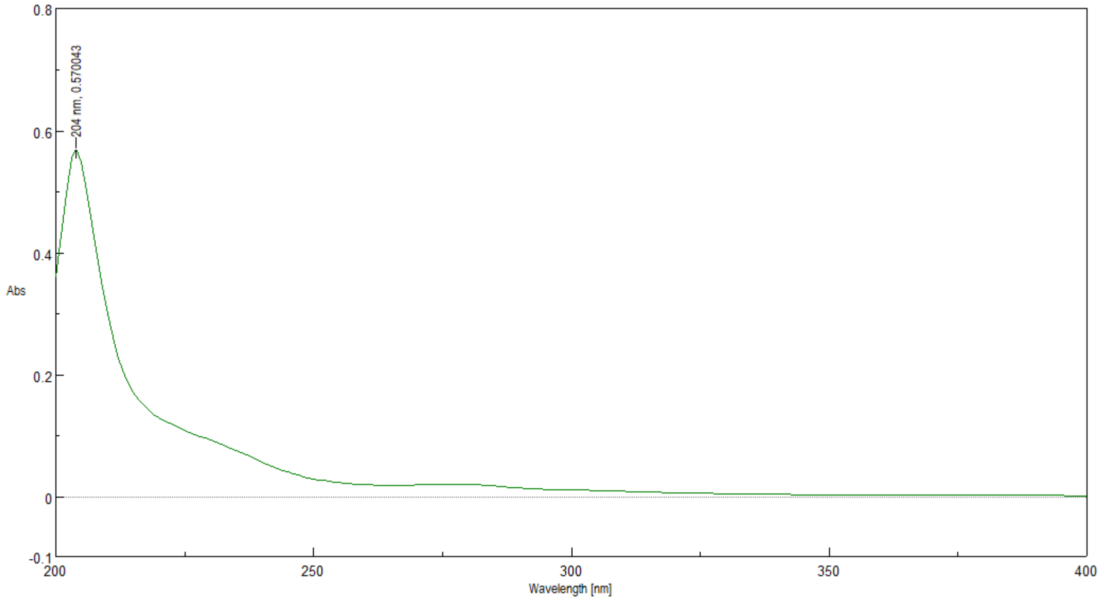

Figure S56 UV spectrum of **6**

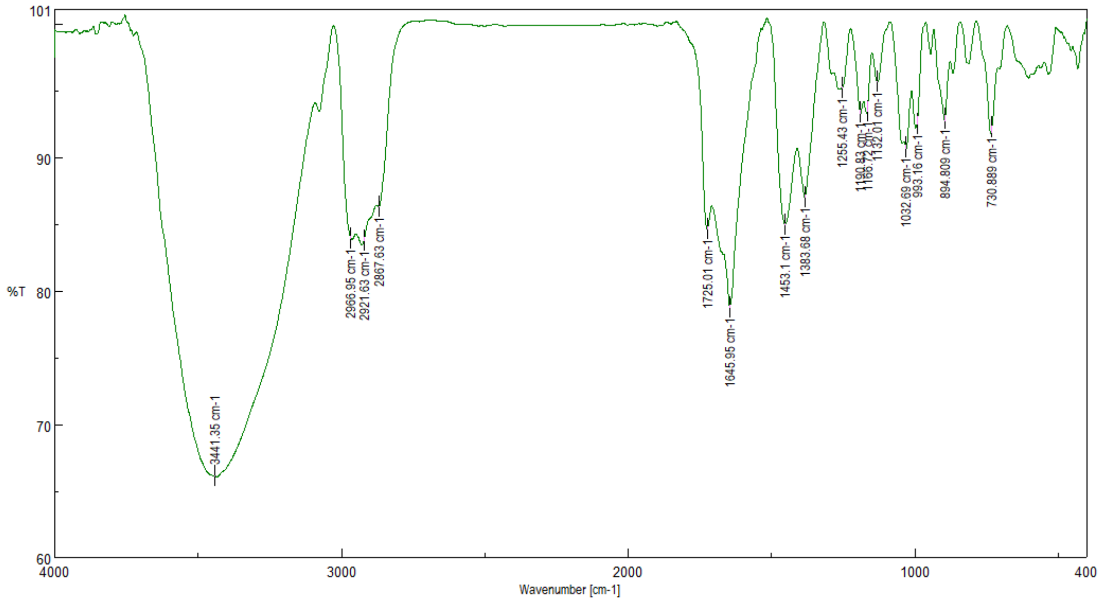

Figure S57 IR spectrum of **6**

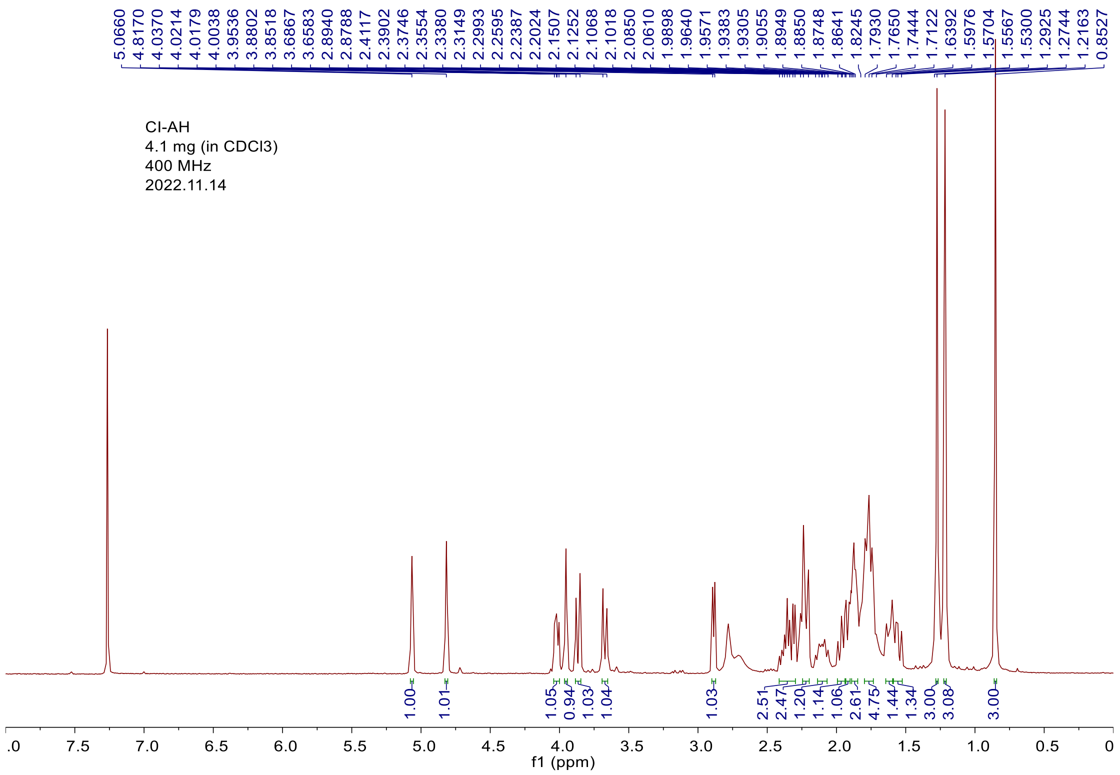

Figure S58 ^{1}H NMR spectrum of **7** (400 MHz, $CDCl_3$)

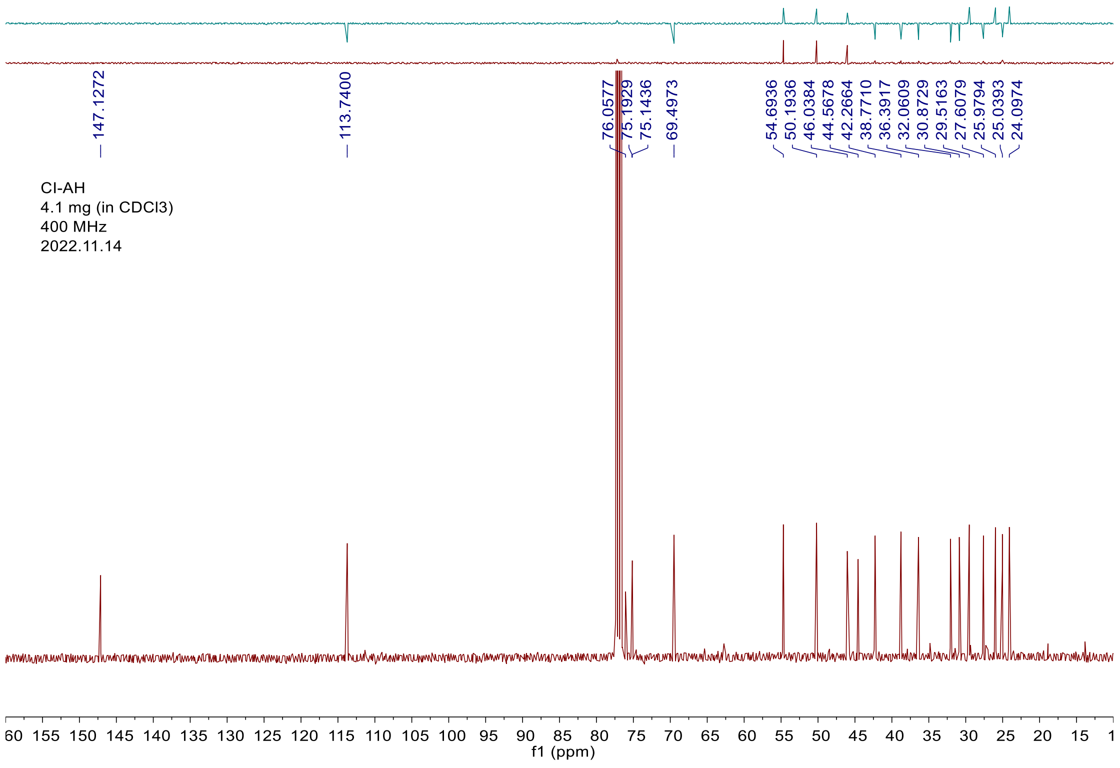

Figure S59 ^{13}C NMR spectrum of **7** (100 MHz, $CDCl_3$)

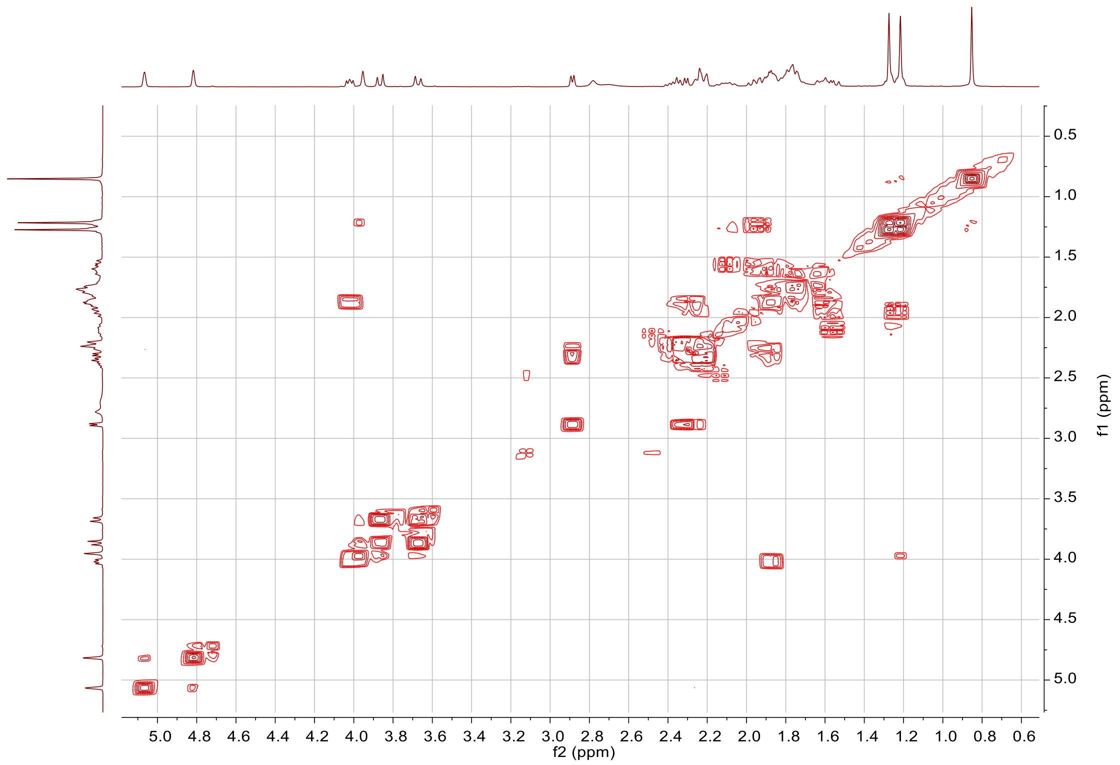

Figure S60 COSY spectrum of **7**

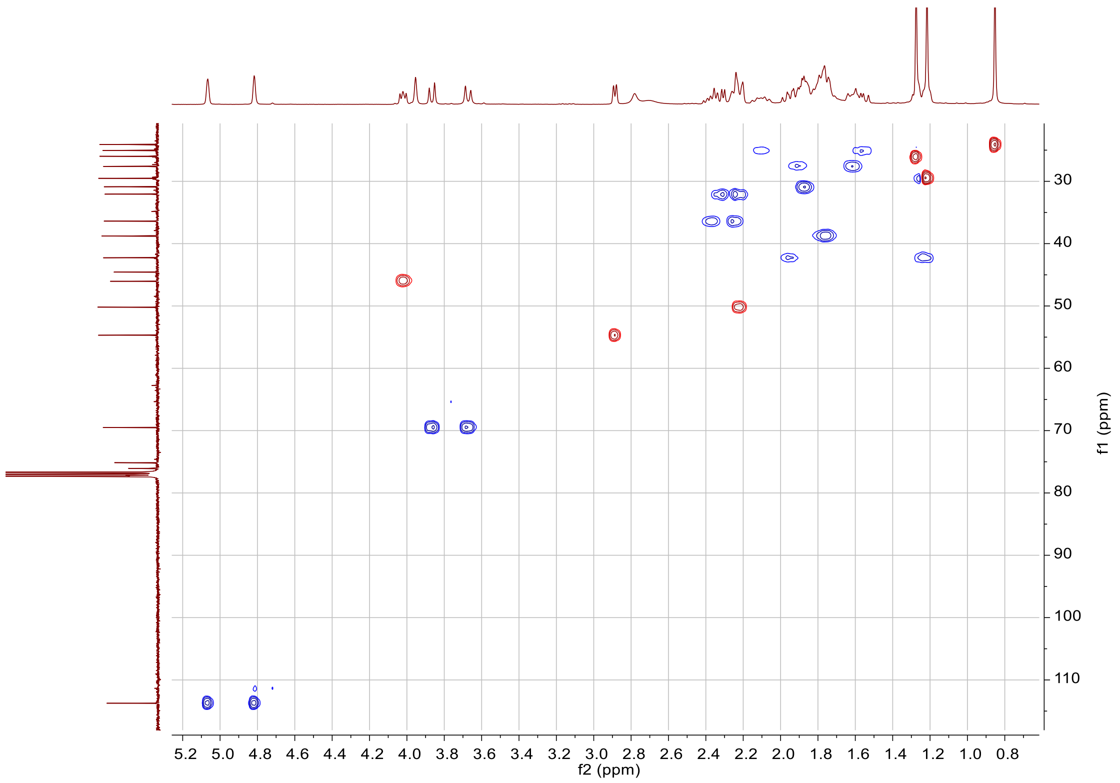

Figure S61 HSQC spectrum of **7**

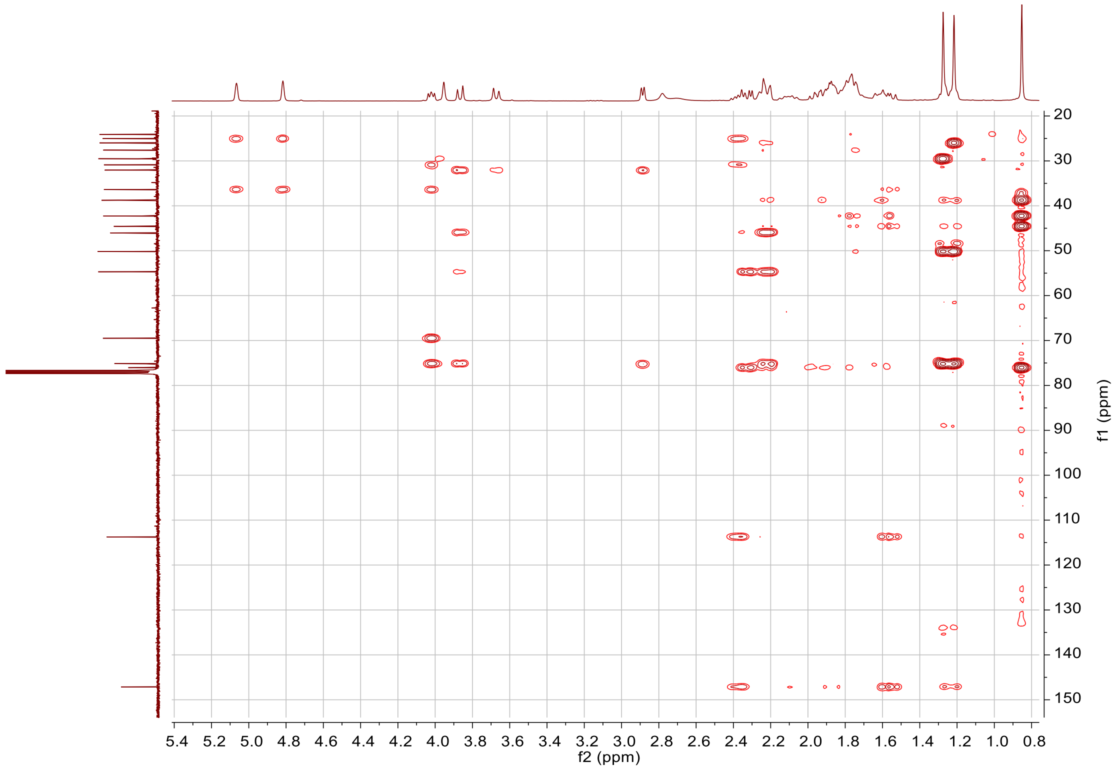

Figure S62 HMBC spectrum of **7**

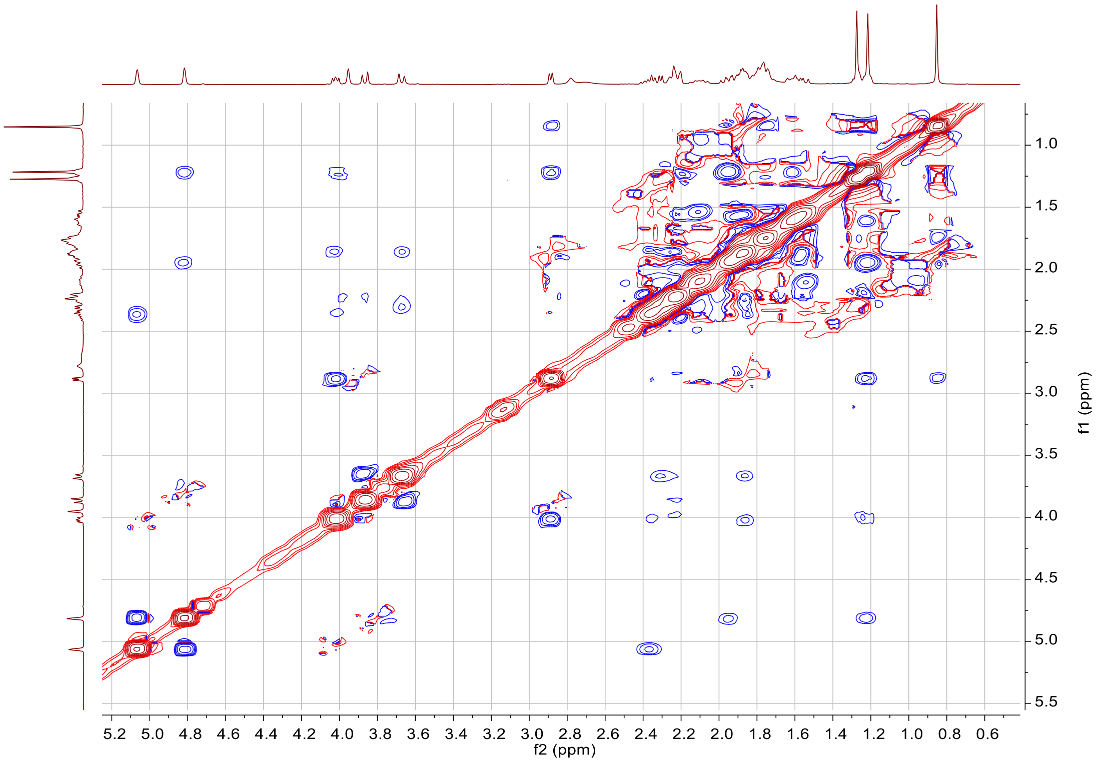

Figure S63 NOESY spectrum of **7**

Mass Spectrum SmartFormula Report

Analysis Info

Analysis Name	D:\1219\CIAHR2.d	12/20/2022 10:41:26 AM
Method	tune_wide_pos_20220422.m	Operator: YU HSIAO-CHING
Sample Name	CI-AH	Instrument: BRUKER micrOTOF-Q
Comment	ESI Positive	

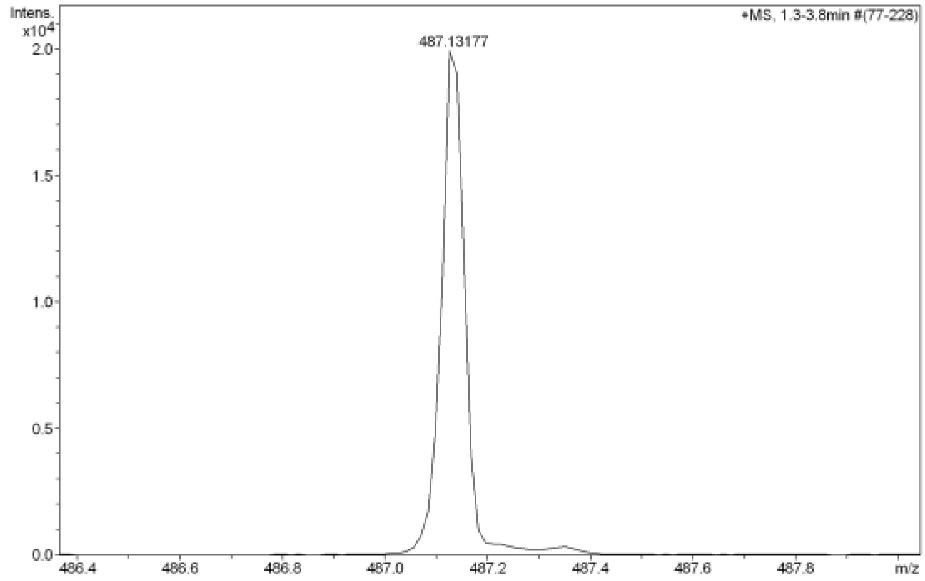

Meas. m/z	#	Formula	Score	m/z	err [mDa]	err [ppm]	mSigma	rdb	e⁻ Conf	N-Rule
487.13177	1	C 20 H 33 I Na O 4	100.00	487.13157	-0.20	-0.41	27.7	3.5	even	ok

Figure S64 HRESIMS spectrum of **7**

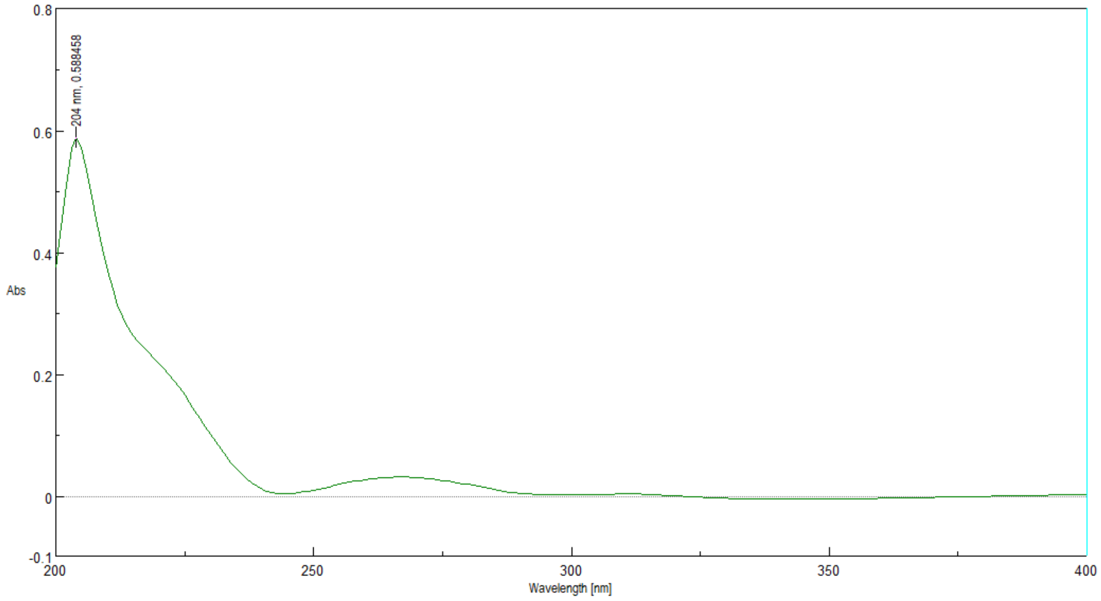

Figure S65 UV spectrum of **7**

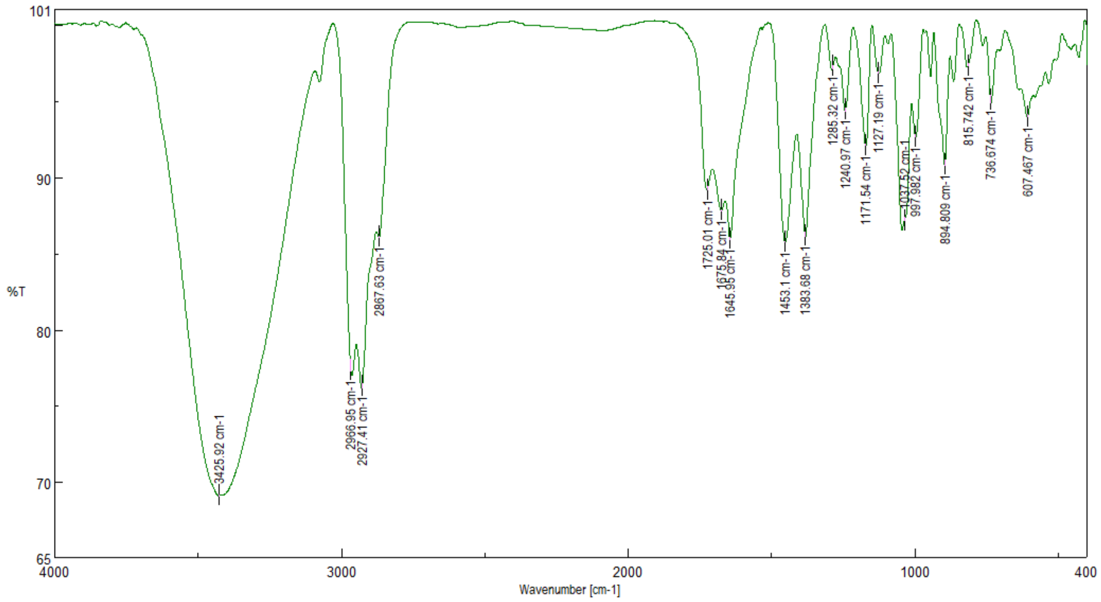

Figure S66 IR spectrum of **7**

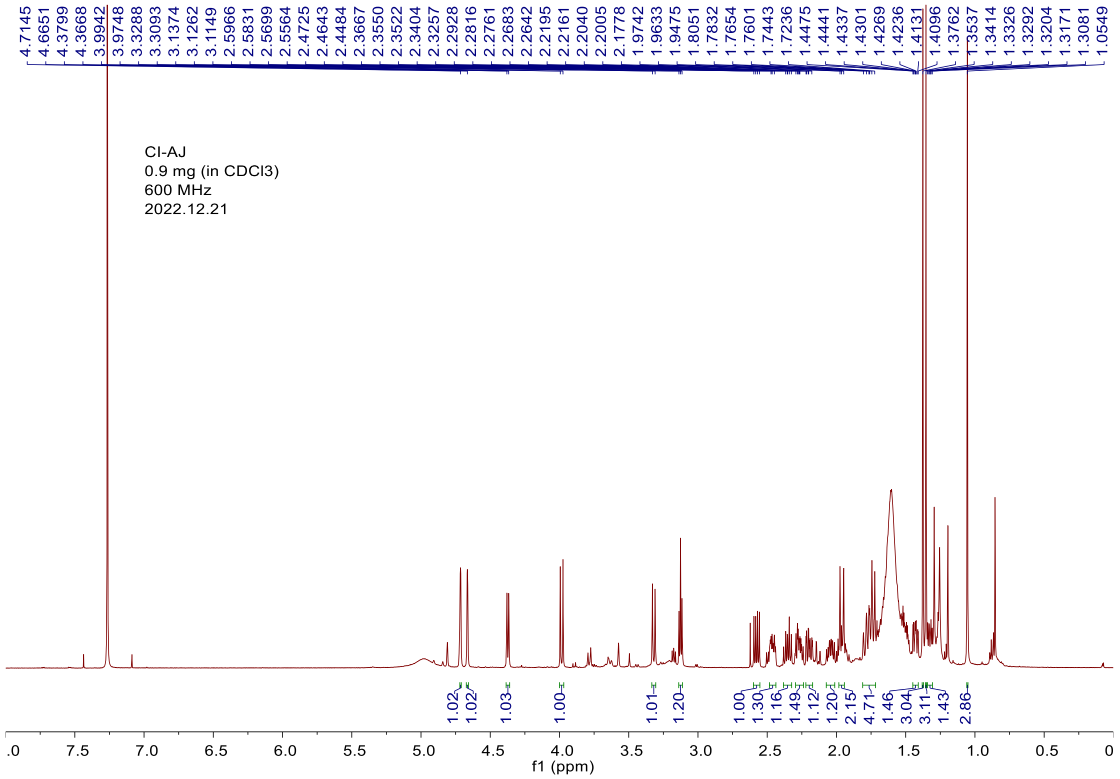

Figure S67 ^{1}H NMR spectrum of **8** (600 MHz, $CDCl_3$)

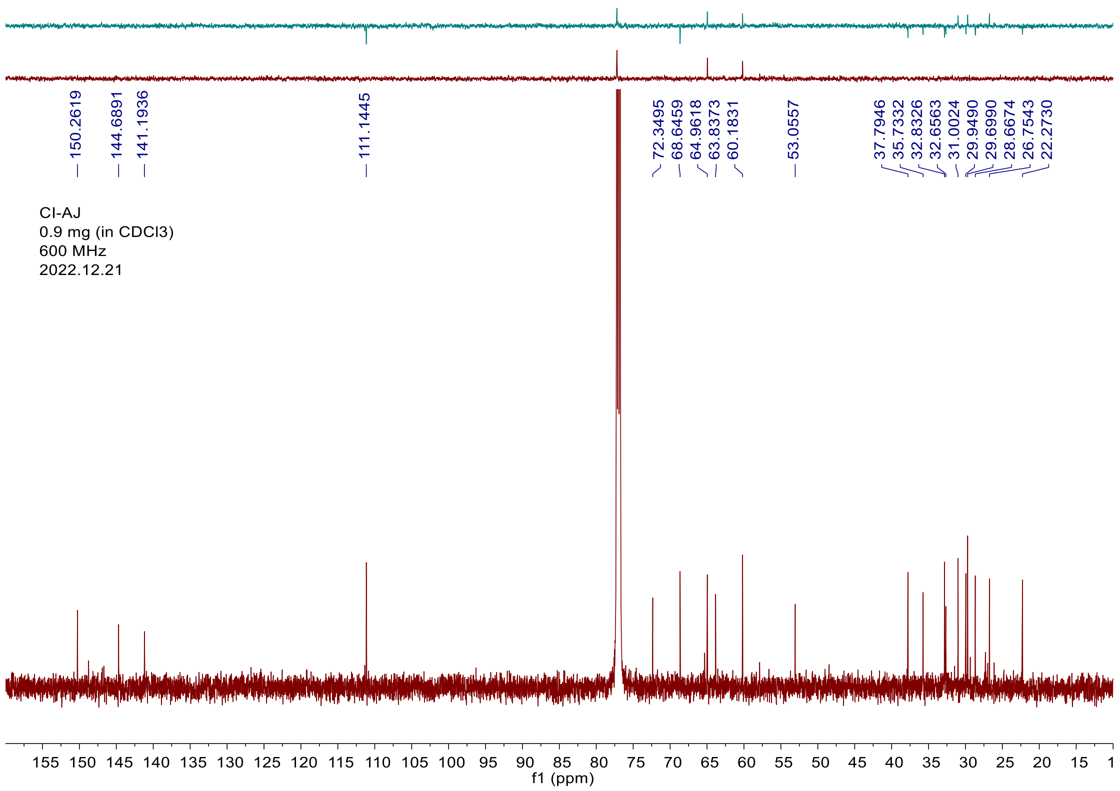

Figure S68 ^{13}C NMR spectrum of **8** (125 MHz, $CDCl_3$)

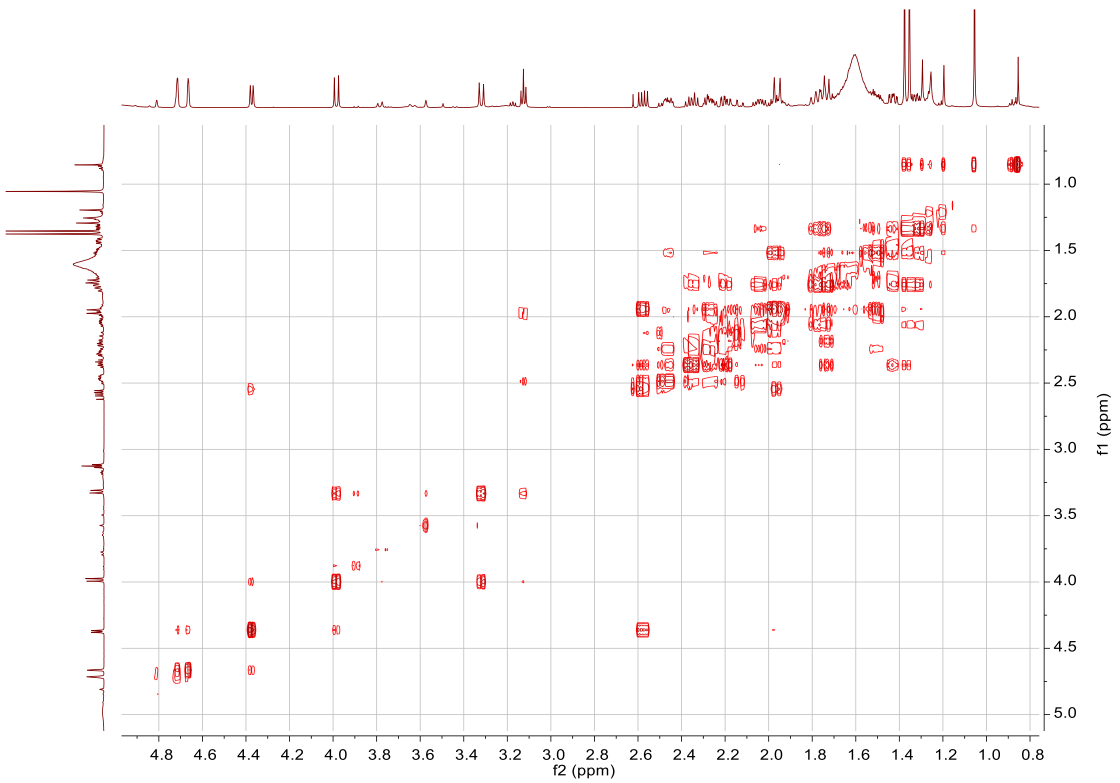

Figure S69 COSY spectrum of **8**

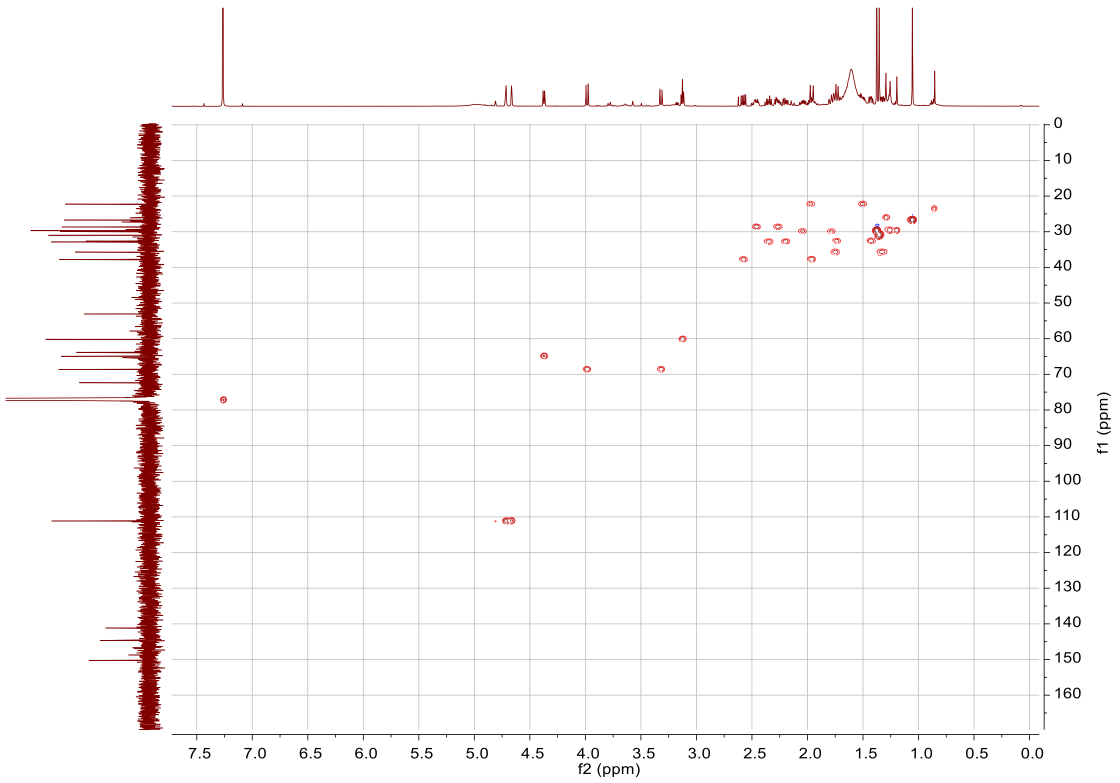

Figure S70 HSQC spectrum of **8**

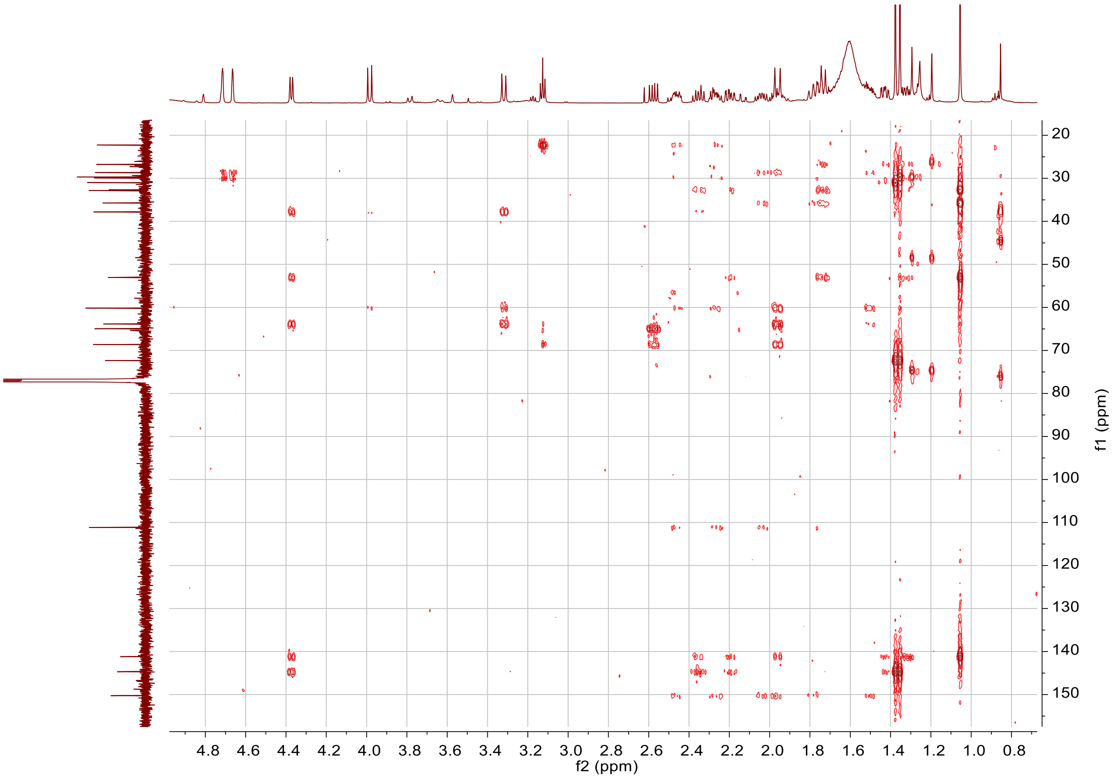

Figure S71 HMBC spectrum of **8**

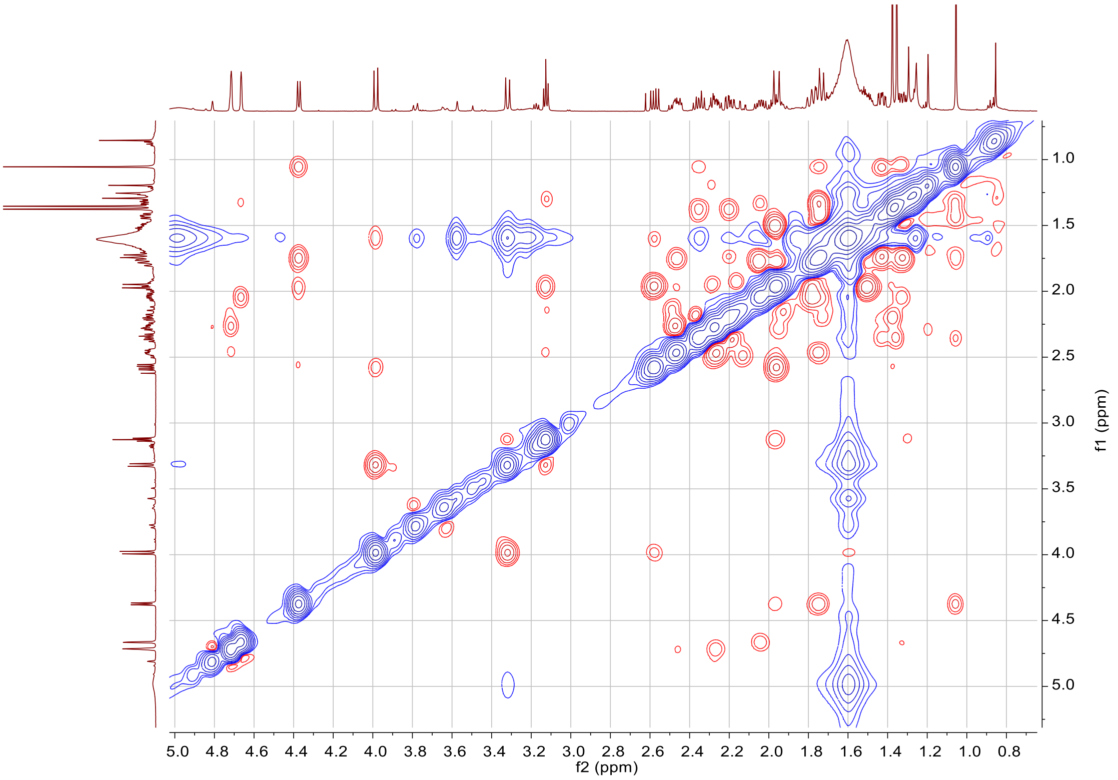

Figure S72 NOESY spectrum of **8**

D:\data\yu\ CI-AJ_20230203032124.raw 2/6/2023 2:29:04 PM

CI-AJ_20230203032124 #93 RT: 0.12 AV: 1 NL: 2.92E7

T: FTMS + p ESI Full ms [100.0000-1000.0000]

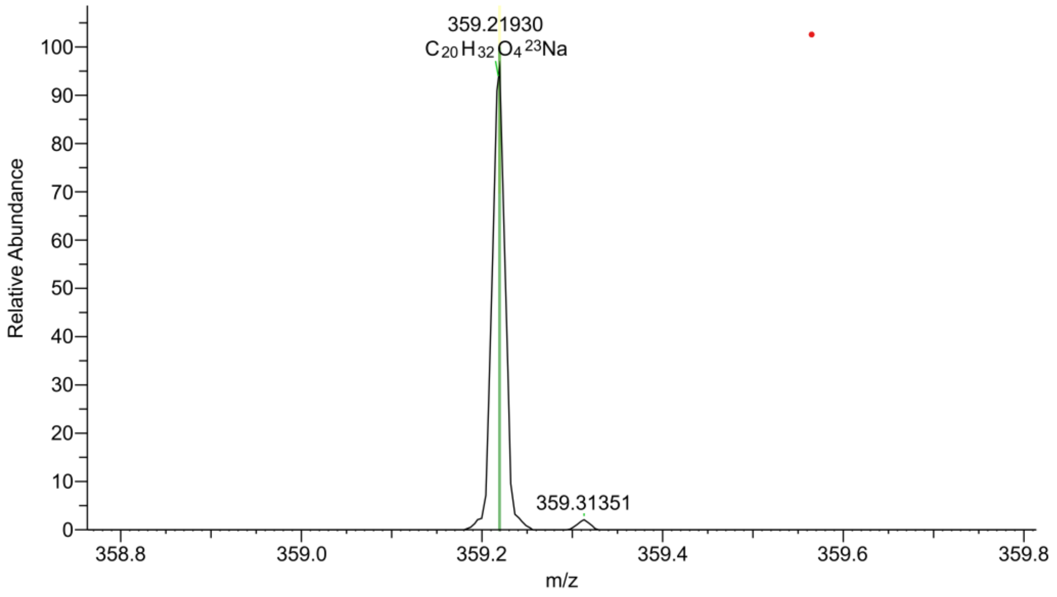

Peak Mass	Display F...	S Fit	RDB	Delta [pp...	Theo. ma...	Rank	Combine...	# Match...	# Missed...	MS Cov....	Pattern C...	MSMS M...
359.21930	$C_{20}H_{32}O_4$ ^{23}Na	28.0578229935457	4.50	0.05	359.21928	1	91.38	2	2	94.9	97.28	(Collection)

Figure S73 HRESIMS spectrum of **8**

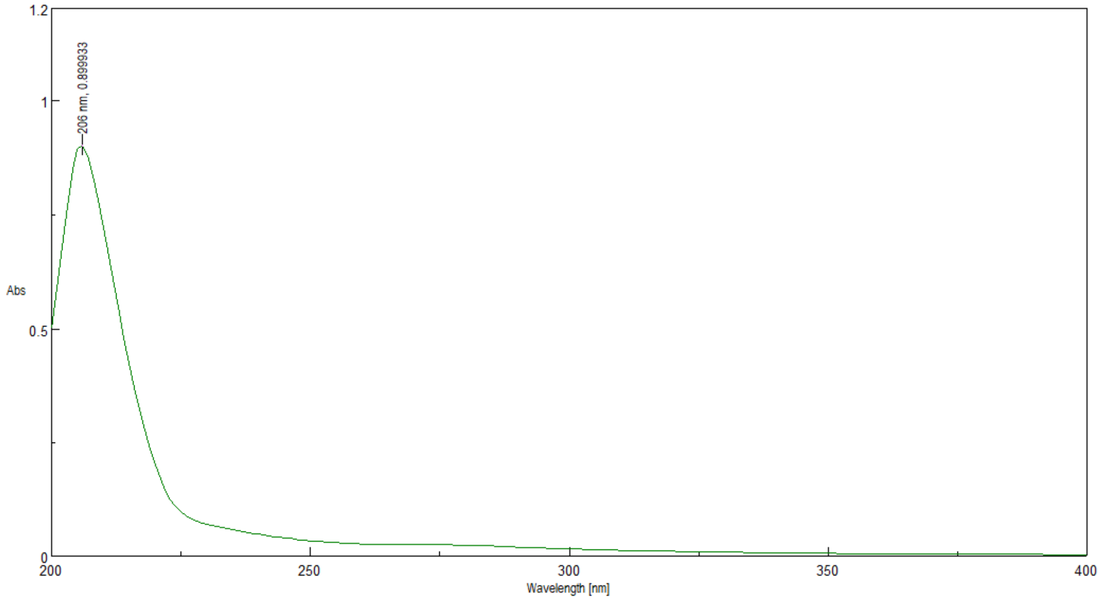

Figure S74 UV spectrum of **8**

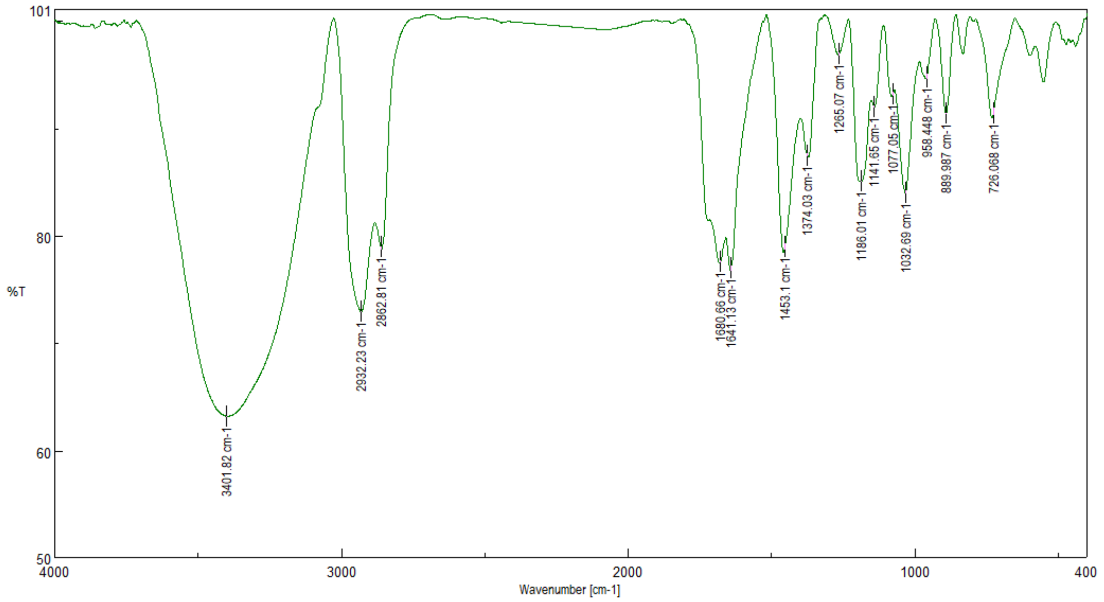

Figure S75 IR spectrum of **8**
